# Supplementary material for: Formal consensus to identify clinically important changes in management resulting from the use of cardiovascular magnetic resonance (CMR) in patients who activate the primary percutaneous coronary intervention (PPCI) pathway
Source: BMJ Open. 2017 Jun 22;7(6):e014627. doi: 10.1136/bmjopen-2016-014627 (PMC5541580; doi:10.1136/bmjopen-2016-014627)
Supplement: Supplementary Appendix 1 [file bmjopen-2016-014627supp001.pdf]

Appendix 1

Literature search

Medline (1950 to Jan 2014)

1 exp Myocardial Infarction/ (142973)
2 myocardial infarct$.tw. (134547)
3 heart attack$.tw. (3601)
4 MI.tw. (25881)
5 ami.tw. (12166)
6 stemi.tw. (3663)
7 heart infarct$.tw. (705)
8 Acute Coronary Syndrome/ (6784)
9 acute coronary syndrome$.tw. (15521)
10 ACS.tw. (9320)
11 or/1-10 (210783)
12 exp angioplasty/ (53568)
13 angioplasty.tw. (34003)
14 percutaneous coronary intervention$.tw. (14406)
15 exp Percutaneous Coronary Intervention/ (34668)
16 PCI.tw. (11157)
17 PPCI.tw. (366)
18 exp Stents/ (50784)
19 stent$.tw. (56427)
20 Myocardial Revascularization/ (9079)
21 revasculari$.tw. (37853)
22 reperfused.tw. (5688)
23 reperfusion.tw. (55775)
24 or/12-23 (191056)
25 exp Magnetic Resonance Imaging/ (295774)
26 Magnetic resonance imag$.tw. (122420)
27 Cardiovascular magnetic resonance.tw. (1765)
28 cardiac magnetic resonance.tw. (3179)
29 MRI.tw. (121901)
30 CMR.tw. (3035)
31 or/25-30 (344849)
32 24 and 31 (5713)
33 11 and 32 (1318)
34 exp animals/ not humans/ (3863199)
35 33 not 34 (1051)

Embase (1980 to Jan 2014)

1 exp heart infarction/ (261299)
2 myocardial infarct$.tw. (185732)
3 heart attack$.tw. (4991)
4 MI.tw. (45694)
5 ami.tw. (19697)
6 stemi.tw. (10463)
7 heart infarct$.tw. (1450)
8 exp Acute Coronary Syndrome/ (26244)
9 acute coronary syndrome$.tw. (28004)
10 ACS.tw. (17708)
11 or/1-10 (337561)

12 exp angioplasty/ (67762)
13 angioplasty.tw. (47179)
14 percutaneous coronary intervention$.tw. (27203)
15 exp Percutaneous Coronary Intervention/ (59160)
16 PCI.tw. (26664)
17 PPCI.tw. (1281)
18 Stent/ (64591)
19 exp cardiovascular stent/ (34380)
20 stent$.tw. (94492)
21 heart muscle revascularization/ (21848)
22 revasculari$.tw. (56445)
23 reperfused.tw. (7304)
24 reperfusion.tw. (77453)
25 or/12-24 (298341)
26 exp nuclear magnetic resonance imaging/ (524106)
27 Magnetic resonance imag$.tw. (164672)
28 Cardiovascular magnetic resonance.tw. (3414)
29 cardiac magnetic resonance.tw. (6341)
30 MRI.tw. (203793)
31 CMR.tw. (7673)
32 or/26-31 (563445)
33 25 and 32 (13408)
34 11 and 33 (3451)
35 (exp animal/ or nonhuman/) not exp humans/ (5173875)
36 34 not 35 (3048)
37 limit 36 to embase (2864)

The Cochrane Library

#1 MeSH descriptor: [Myocardial Infarction] explode all trees
#2 MeSH descriptor: [Acute Coronary Syndrome] explode all trees
#3 myocardial next infarct*
#4 heart next attack*
#5 MI
#6 AMI
#7 STEMI
#8 heart next infarct*
#9 "acute coronary syndrome*"
#10 #1 or #2 or #3 or #4 or #5 or #6 or #7 or #8 or #9
#11 MeSH descriptor: [Angioplasty] explode all trees
#12 MeSH descriptor: [Percutaneous Coronary Intervention] explode all trees
#13 MeSH descriptor: [Stents] explode all trees
#14 MeSH descriptor: [Myocardial Revascularization] this term only
#15 angioplasty
#16 "percutaneous coronary intervention*"
#17 PCI or PPCI
#18 stent*
#19 revasculari*
#20 reperfused
#21 reperfusion
#22 #11 or #12 or #13 or #14 or #15 or #16 or #17 or #18 or #19 or #20 or #21
#23 MeSH descriptor: [Magnetic Resonance Imaging] explode all trees
#24 "Magnetic resonance imag*"
#25 " Cardiovascular magnetic resonance"
#26 "cardiac magnetic resonance"

#27 MRI or CMR
#28 #23 or #24 or #25 or #26 or #27
#29 #11 and #22 and #28

ISI Web of Science

4 1,351 #3 AND #2 AND #1
3 218,389 TOPIC: (Angioplasty or “percutaneous coronary intervention*” or PCI or PPCI or stent* or revasulari* or reperfused or reperfusion)
2 257,465 TOPIC: (“Magnetic Resonance Imag*” or “Cardiovascular magnetic resonance” or “ cardiac magnetic resonance” or MRI or CMR)
1 273,481 TOPIC: (“myocardial infarct*” or “heart attack*” or “heart infarct*” or “acute coronary syndrome*” or MI or AMI or STEMI or ACS)

BIOSIS

6 873 #5 AND #4
5 TAXONOMIC DATA: (human not (animal* not human))
4 #3 AND #2 AND #1
3 TOPIC: (Angioplasty or “percutaneous coronary intervention*” or PCI or PPCI or stent* or revasulari* or reperfused or reperfusion)
2 TOPIC: (“Magnetic Resonance Imag*” or “Cardiovascular magnetic resonance” or “ cardiac magnetic resonance” or MRI or CMR)
1 TOPIC: (“myocardial infarct*” or “heart attack*” or “heart infarct*” or “acute coronary syndrome*” or MI or AMI or STEMI or ACS)
