# Supplementary material for: Formal consensus to identify clinically important changes in management resulting from the use of cardiovascular magnetic resonance (CMR) in patients who activate the primary percutaneous coronary intervention (PPCI) pathway
Source: BMJ Open. 2017 Jun 22;7(6):e014627. doi: 10.1136/bmjopen-2016-014627 (PMC5541580; doi:10.1136/bmjopen-2016-014627)
Supplement: Supplementary Appendix 2 [file bmjopen-2016-014627supp002.pdf]

Appendix 2

First survey (statements, supporting paragraphs and references)

Benefit to patients and the NHS of cardiac magnetic resonance imaging (CMR) after Primary percutaneous coronary Intervention (PPCI) Pathway Activation

Thank you for agreeing to complete this survey. We expect the survey to take about 20 minutes of your time. You are asked to read and rate 12 statements relating to the potential impact of cardiac magnetic resonance imaging (CMR) in changing the management of patients in a clinically important way. The patient population under consideration includes all patients who activate the primary percutaneous coronary intervention (PPCI) pathway. Please rate your agreement with each statement on the scale provided. Each statement has a supporting paragraph that provides background information and links to full text references. We believe we have used the best available evidence but we are aware that the evidence is of variable quality.

In order to progress through the survey, please use the following navigation buttons:

Click the Next button to go to the next page.

Click the Previous button to return to the previous page.

Click the Number buttons in the text to access the full references.

Click the Submit button to submit the survey at the end.

Statement 1. *Compared with echocardiography, CMR after PPCI allows patients with CMR markers that indicate* ***poor*** *prognosis (e.g. impaired left ventricular function, large infarct size, microvascular obstruction) to be followed up more appropriately and undergo more aggressive medical therapy for secondary prevention.*

Accurate assessment of infarct characteristics is important for risk stratification after PPCI. CMR can quantify in a single scan all cardiac markers relevant to PPCI outcomes, with high reproducibility and accuracy. CMR has high spatial and temporal resolution and is superior to echocardiography for measuring left ventricular volumes and ejection fraction. **[1]** Additionally, CMR markers such as infarct size, microvascular obstruction and myocardial salvage have been shown to have long term prognostic value. **[2-4]** Late gadolinium enhancement CMR has added prognostic value over echocardiography. **[5]**

Statement 2. *Compared with echocardiography, CMR after PPCI allows patients with CMR markers that indicate* ***good*** *prognosis (e.g. normal left ventricular function, high myocardial salvage, no microvascular obstruction, no residual ischemia) to be discharged earlier and followed up less frequently.*

CMR measures several markers (infarct size, myocardial salvage index, microvascular obstruction, myocardial oedema and haemorrhage) that cannot be measured by echocardiography. These markers can be used to predict cardiac remodelling and prognosis after myocardial infarction, **[2, 4]** determine the optimal therapeutic pathway for each patient and prevent over- and under-treatment.

Statement 3. *Compared with echocardiography, CMR better identifies the cause of out of hospital cardiac arrest [e.g. large myocardial infarction, arrhythmogenic right ventricular cardiomyopathy (ARVC), aberrant coronary arteries, hypertrophic cardiomyopathy (HCM)] to optimise further treatment for the patient (e.g. defibrillator for primary arrhythmia or percutaneous coronary intervention) or family members.*

Out-of-hospital cardiac arrest affects about 60,000 people in the UK each year. Currently, hospital survival is about 32%. Early identification of the cause is essential to improve survival. Causes of out-of-hospital cardiac arrest include myocardial infarction (40%-90%) and inherited cardiomyopathies such as ARVC and HCM. Unlike echocardiography, CMR allows in vivo tissue characterisation, which differentiates scarring resulting from myocardial infarction from other causes of focal fibrosis (e.g. observed in non-ischaemic cardiomyopathies such as ARVC and HCM). **[6]** Late gadolinium enhancement on CMR can predict serious cardiac complications in patients with HCM (all-cause death, cardiac death and death from heart failure), **[7]** and identifies patients that may need more aggressive medical and device therapy (e.g. renin-aldosterone system inhibition for prevention of heart failure or implantable cardioverter defibrillator placement for primary prevention of sudden cardiac death).

Statement 4. *CMR after PPCI identifies patients at high risk of having ventricular septal defect (VSD) or impending cardiac rupture, who may require ventricular patch or other urgent cardiac surgery, and guides the optimal management of these patients.*

VSD and left ventricular free wall rupture are rare complications of myocardial infarction, occurring in less than 1% of PPCI patients, usually within one week of the infarct. Mortality ranges between 50% and 80%. Diagnosis can be difficult to make and usually requires multi-modality imaging (echocardiography, ventriculography, computed tomography, CMR) before surgical repair. Because of its high spatial resolution, CMR can be used to clarify the detailed structure of these lesions. CMR accurately identifies the location, size and tissue margins of VSD and is useful for detecting apical defects, which are not easily identified by echocardiography. CMR measurements may also be used to determine the size of ventricular patch required to close the VSD, which avoids the inflation of a sizing balloon in friable infarcted tissue. **[8]** These features, together with other CMR markers of damage (e.g. infarct size, microvascular obstruction, left ventricular dysfunction) are useful for guiding optimal management of patients with post-infarct VSD. Similarly, CMR can differentiate between the different types of impending free wall rupture (acute, sub-acute and chronic), which may be helpful in planning surgical management. **[9]**

Statement 5. *In patients with a "normal" (unobstructed) coronary angiogram, CMR can differentiate patients who have had a myocardial infarction with spontaneous reperfusion or distal embolization from patients with a non-ischaemic diagnosis (e.g. myocarditis, Takotsubo cardiomyopathy, aortic dissection), resulting in a patient treatment plan appropriate for the definitive diagnosis.*

The incidence of unobstructed coronary angiogram in patients who activate the PPCI pathway is between 5% and 12%. In these patients, the lack of an accurate diagnosis may result in inappropriate or unnecessary treatment and/or follow-up and poorer prognosis. **[10]**

CMR facilitates differential diagnosis in the context of an unobstructed coronary angiogram, providing a definitive diagnosis (e.g. myocardial infarction, myocarditis, Takotsubo cardiomyopathy) in 65-90% of these patients. **[11, 12]** In patients with myocarditis, late gadolinium enhancement (LGE) on CMR may predict long term adverse outcomes. **[13]** In the context of myocardial infarction without an angiographic lesion, CMR can locate the culprit infarct-related artery in patients with spontaneous reperfusion or with distal embolization.

Statement 6. *Compared with echocardiography, CMR after PPCI better identifies patients at high risk of sudden cardiac death who* ***would*** *benefit most from an implantable cardiac device [e.g.* implantable cardioverter defibrillator (*ICD), cardiac resynchronisation therapy, (CRT)].*

About 6% of heart attack patients subsequently die suddenly from a presumed cardiac cause. Current guidelines recommend the use of ICD to prevent sudden cardiac death in patients who have a low left ventricular ejection fraction (EF) after a heart attack. EF is used in clinical practice to make decisions about ICD implantation, but it has a low predictive value and many patients with an ICD will never benefit from it. **[14]** EF is most commonly measured by echocardiography, but CMR is now considered the gold standard for EF measurement because it is more reproducible than echocardiography. **[1]** CMR has been shown to be better than echocardiography for selecting patients for ICD implantation when strict EF thresholds are used to guide implantation. **[15]** The extent of myocardial scar characterised by late gadolinium enhancement (LGE) CMR may also be used to predict whether ICD implantation is appropriate in this patient group. **[16]** Furthermore, LGE CMR can guide placement of the left ventricular lead away from scarred myocardium, which results in a better clinical outcome after CRT. **[17]**

Statement 7. *Compared with echocardiography, CMR after PPCI better identifies patients who* ***would not*** *benefit from cardiac resynchronisation therapy (CRT).*

CRT (or biventricular pacing) uses a specialised pacemaker to resynchronise the beating of the two ventricles by pacing both simultaneously, which improves the contraction of the left ventricle and the overall efficiency of the heart. It is used in patients with systolic ventricular dysfunction and heart failure. However, about 30% of patients who meet the inclusion criteria for CRT do not respond to it. **[18]** CMR assessment of mechanical dyssynchrony and myocardial scar provides additional value over echocardiography for identifying responsive patients. **[19, 20]** Myocardial scar is an important feature of non-response to CRT **[21, 22]** and late gadolinium enhancement (LGE) CMR accurately differentiates between transmural, mid-myocardial, epicardial, and subendocardial scar.

Statement 8. *Compared with stress echocardiography* or *single photon emission computed tomography (SPECT), CMR after PPCI in patients with multi-vessel disease better assesses ischaemia and viability of the myocardium to optimise the revascularisation strategy for the patient and avoid additional diagnostic tests.*

Between 40% and 65% of the patients who activate the PPCI pathway have multi-vessel disease. Adequate assessment of residual ischemia in non-culprit arteries post PPCI is important for effective management because this patient group has an adverse prognosis. Various techniques are currently used to assess the need for additional revascularisation, including stress echocardiography, SPECT and stress-perfusion CMR. Stress CMR has excellent prognostic value **[23]** and better diagnostic accuracy than SPECT **[24, 25]** or stress echocardiography **[26]** for detecting angiographically significant coronary artery disease.

Statement 9. *Compared with echocardiography, CMR after PPCI better identifies patients with left ventricular (LV) thrombus for treatment with anticoagulation therapy.*

LV thrombus is a serious complication of acute myocardial infarction. It increases risk of thromboembolic events, particularly stroke. LV thrombus develops in up to 10% of patients with anterior wall infarctions after PPCI. Although echocardiography is most commonly used to detect LV thrombus and to assess its shape and size, between 10-46% of echocardiograms are inconclusive. **[27]** Late gadolinium enhancement (LGE) CMR is considered the gold standard for detecting LV thrombus, because it detects thrombus based on tissue characteristics rather than anatomic appearance. Fewer thrombi are detected by contrast-echocardiography than by LGE CMR. **[28]** CMR has a higher sensitivity (88%) than contrast (61%) and noncontrast (<33%) echocardiography. **[28, 29]**

Statement 10. *Compared with other imaging modalities, in patients with multi-vessel disease, CMR after PPCI better identifies the artery that caused the myocardial infarction and guides the subsequent treatment plan in relation to additional revascularisation (percutaneous coronary intervention, or coronary artery bypass graft surgery).*

In patients with multi vessel disease presenting with acute MI, the culprit artery is not always easy to identify as more than one artery could be responsible. CMR has higher spatial resolution than single photon emission computed tomography (SPECT), allowing easy identification of the infarct-related artery and non-culprit territory stenosis in PPCI patients, **[30]** and hence appropriate risk-stratification of patients with multi-vessel disease for planning their future treatment. **[31]**

Statement 11. *Compared with echocardiography, CMR after PPCI better identifies incidental cardiac findings that may need further investigation and treatment.*

Compared with echocardiography, CMR provides versatile imaging planes, superior tissue contrast and advanced tissue characterisation, allowing a comprehensive assessment of cardiac anatomy, function and flow, and imaging of the great vessels (including venous return), the pericardium and suspected cardiac tumours. **[32]** As such, CMR can identify congenital coronary anomalies, cardiac masses, coronary artery aneurysms, valvular heart disease, thoracic aortic disease, etc. CMR can adequately differentiate benign from malignant tumours in the heart. **[33]**

Statement 12. *Compared with echocardiography, CMR after PPCI identifies significant incidental non-cardiac findings (e.g. oesophageal and lung tumours, pulmonary embolus, aortic aneurysm) that may need further investigation and treatment.*

Unlike echocardiography, which provides limited imaging of mediastinal and extra-cardiac structures, CMR images a substantial part of the thorax and abdomen in the field of view, which may potentially contain non-cardiac abnormalities. The prevalence of non-cardiac findings on CMR is up to 80% (with up to 30% of these representing potentially significant findings), depending on the characteristics of the population examined. **[34-36]**

References

1. Dorosz JL, Lezotte DC, Weitzenkamp DA, Allen LA, Salcedo EE: **Performance of 3-dimensional echocardiography in measuring left ventricular volumes and ejection fraction: a systematic review and meta-analysis**. *J Am Coll Cardiol* 2012, **59**(20):1799-1808.

2. Eitel I, Desch S, de WS, Fuernau G, Gutberlet M, Schuler G, Thiele H: **Long-term prognostic value of myocardial salvage assessed by cardiovascular magnetic resonance in acute reperfused myocardial infarction**. *Heart* 2011, **97**(24):2038-2045.
3. Eitel I, Desch S, Fuernau G, Hildebrand L, Gutberlet M, Schuler G, Thiele H: **Prognostic significance and determinants of myocardial salvage assessed by cardiovascular magnetic resonance in acute reperfused myocardial infarction**. *J Am Coll Cardiol* 2010, **55**(22):2470-2479.
4. Klug G, Mayr A, Schenk S, Esterhammer R, Schocke M, Nocker M, Jaschke W, Pachinger O, Metzler B: **Prognostic value at 5 years of microvascular obstruction after acute myocardial infarction assessed by cardiovascular magnetic resonance**. *Journal of Cardiovascular Magnetic Resonance* 2012, **14**:46.
5. Wong TC, Piehler K, Puntil KS, Moguillansky D, Meier CG, Lacomis JM, Kellman P, Cook SC, Schwartzman DS, Simon MA *et al*: **Effectiveness of late gadolinium enhancement to improve outcomes prediction in patients referred for cardiovascular magnetic resonance after echocardiography**. *Journal of cardiovascular magnetic resonance : official journal of the Society for Cardiovascular Magnetic Resonance* 2013, **15**:6.
6. Parsai C, O'Hanlon R, Prasad SK, Mohiaddin RH: **Diagnostic and prognostic value of cardiovascular magnetic resonance in non-ischaemic cardiomyopathies**. *Journal of cardiovascular magnetic resonance : official journal of the Society for Cardiovascular Magnetic Resonance* 2012, **14**:54.
7. Green JJ, Berger JS, Kramer CM, Salerno M: **Prognostic value of late gadolinium enhancement in clinical outcomes for hypertrophic cardiomyopathy**. *JACC Cardiovascular imaging* 2012, **5**(4):370-377.
8. Artis NJ, Thomson J, Plein S, Greenwood JP: **Percutaneous closure of postinfarction ventricular septal defect: cardiac magnetic resonance-guided case selection and postprocedure evaluation**. *The Canadian journal of cardiology* 2011, **27**(6):869 e863-865.
9. Galvin S, Chen V, Bunton R, Doyle T: **Sutureless pericardial patch augmentation for impending left ventricular free wall rupture**. *Interactive cardiovascular and thoracic surgery* 2010, **10**(2):341-343.
10. Dokainish H, Pillai M, Murphy SA, DiBattiste PM, Schweiger MJ, Lotfi A, Morrow DA, Cannon CP, Braunwald E, Lakkis N: **Prognostic implications of elevated troponin in patients with suspected acute coronary syndrome but no critical epicardial coronary disease: a TACTICS-TIMI-18 substudy**. *J Am Coll Cardiol* 2005, **45**(1):19-24.
11. Assomull RG, Lyne JC, Keenan N, Gulati A, Bunce NH, Davies SW, Pennell DJ, Prasad SK: **The role of cardiovascular magnetic resonance in patients presenting with chest pain, raised troponin, and unobstructed coronary arteries**. *Eur Heart J* 2007, **28**(10):1242-1249.
12. Leurent G, Langella B, Fougerou C, Lentz PA, Larralde A, Bedossa M, Boulmier D, Le Breton H: **Diagnostic contributions of cardiac magnetic resonance imaging in patients presenting with elevated troponin, acute chest pain syndrome and unobstructed coronary arteries**. *Arch Cardiovasc Dis* 2011, **104**(3):161-170.
13. Grun S, Schumm J, Greulich S, Wagner A, Schneider S, Bruder O, Kispert EM, Hill S, Ong P, Klingel K *et al*: **Long-term follow-up of biopsy-proven viral myocarditis: predictors of mortality and incomplete recovery**. *J Am Coll Cardiol* 2012, **59**(18):1604-1615.
14. Moss AJ, Greenberg H, Case RB, Zareba W, Hall WJ, Brown MW, Daubert JP, McNitt S, Andrews ML, Elkin AD *et al*: **Long-term clinical course of patients after termination of ventricular tachyarrhythmia by an implanted defibrillator**. *Circulation* 2004, **110**(25):3760-3765.
15. Joshi SB, Connelly KA, Jimenez-Juan L, Hansen M, Kirpalani A, Dorian P, Mangat I, Al-Hesayen A, Crean AM, Wright GA *et al*: **Potential clinical impact of cardiovascular magnetic resonance assessment of ejection fraction on eligibility for cardioverter defibrillator**

implantation. *Journal of cardiovascular magnetic resonance : official journal of the Society for Cardiovascular Magnetic Resonance* 2012, **14**:69.
16. Alexandre J, Saloux E, Dugue AE, Lebon A, Lemaitre A, Roule V, Labombarda F, Provost N, Gomes S, Scanu P *et al*: **Scar extent evaluated by late gadolinium enhancement CMR: a powerful predictor of long term appropriate ICD therapy in patients with coronary artery disease**. *Journal of cardiovascular magnetic resonance : official journal of the Society for Cardiovascular Magnetic Resonance* 2013, **15**:12.
17. Leyva F, Foley PW, Chalil S, Ratib K, Smith RE, Prinzen F, Auricchio A: **Cardiac resynchronization therapy guided by late gadolinium-enhancement cardiovascular magnetic resonance**. *Journal of cardiovascular magnetic resonance : official journal of the Society for Cardiovascular Magnetic Resonance* 2011, **13**:29.
18. Leclercq C, Kass DA: **Retiming the failing heart: principles and current clinical status of cardiac resynchronization**. *J Am Coll Cardiol* 2002, **39**(2):194-201.
19. Bilchick KC, Dimaano V, Wu KC, Helm RH, Weiss RG, Lima JA, Berger RD, Tomaselli GF, Bluemke DA, Halperin HR *et al*: **Cardiac magnetic resonance assessment of dyssynchrony and myocardial scar predicts function class improvement following cardiac resynchronization therapy**. *JACC Cardiovascular imaging* 2008, **1**(5):561-568.
20. Ellims AH, Pfluger H, Elsik M, Butler MJ, Hare JL, Taylor AJ: **Utility of cardiac magnetic resonance imaging, echocardiography and electrocardiography for the prediction of clinical response and long-term survival following cardiac resynchronisation therapy**. *Int J Cardiovasc Imaging* 2013, **29**(6):1303-1311.
21. Adelstein EC, Saba S: **Scar burden by myocardial perfusion imaging predicts echocardiographic response to cardiac resynchronization therapy in ischemic cardiomyopathy**. *Am Heart J* 2007, **153**(1):105-112.
22. White JA, Yee R, Yuan X, Krahn A, Skanes A, Parker M, Klein G, Drangova M: **Delayed enhancement magnetic resonance imaging predicts response to cardiac resynchronization therapy in patients with intraventricular dyssynchrony**. *J Am Coll Cardiol* 2006, **48**(10):1953-1960.
23. Lipinski MJ, McVey CM, Berger JS, Kramer CM, Salerno M: **Prognostic value of stress cardiac magnetic resonance imaging in patients with known or suspected coronary artery disease: a systematic review and meta-analysis**. *J Am Coll Cardiol* 2013, **62**(9):826-838.
24. Greenwood JP, Maredia N, Younger JF, Brown JM, Nixon J, Everett CC, Bijsterveld P, Ridgway JP, Radjenovic A, Dickinson CJ *et al*: **Cardiovascular magnetic resonance and single-photon emission computed tomography for diagnosis of coronary heart disease (CE-MARC): a prospective trial**. *Lancet* 2012, **379**(9814):453-460.
25. Schwitter J, Wacker CM, Wilke N, Al-Saadi N, Sauer E, Huettle K, Schonberg SO, Debl K, Strohm O, Ahlstrom H *et al*: **Superior diagnostic performance of perfusion-cardiovascular magnetic resonance versus SPECT to detect coronary artery disease: The secondary endpoints of the multicenter multivendor MR-IMPACT II (Magnetic Resonance Imaging for Myocardial Perfusion Assessment in Coronary Artery Disease Trial)**. *Journal of cardiovascular magnetic resonance : official journal of the Society for Cardiovascular Magnetic Resonance* 2012, **14**:61.
26. Nagel E, Lehmkuhl HB, Bocksch W, Klein C, Vogel U, Frantz E, Ellmer A, Dreysse S, Fleck E: **Noninvasive diagnosis of ischemia-induced wall motion abnormalities with the use of high-dose dobutamine stress MRI: comparison with dobutamine stress echocardiography**. *Circulation* 1999, **99**(6):763-770.
27. Delewi R, Zijlstra F, Piek JJ: **Left ventricular thrombus formation after acute myocardial infarction**. *Heart* 2012, **98**(23):1743-1749.
28. Weinsaft JW, Kim RJ, Ross M, Krauser D, Manoushagian S, LaBounty TM, Cham MD, Min JK, Healy K, Wang Y *et al*: **Contrast-enhanced anatomic imaging as compared to contrast-**

enhanced tissue characterization for detection of left ventricular thrombus. *JACC Cardiovascular imaging* 2009, **2**(8):969-979.
29. Srichai MB, Junor C, Rodriguez LL, Stillman AE, Grimm RA, Lieber ML, Weaver JA, Smedira NG, White RD: **Clinical, imaging, and pathological characteristics of left ventricular thrombus: a comparison of contrast-enhanced magnetic resonance imaging, transthoracic echocardiography, and transesophageal echocardiography with surgical or pathological validation**. *Am Heart J* 2006, **152**(1):75-84.
30. Wong DT, Leung MC, Das R, Liew GY, Williams K, Dundon BK, Molaee P, Teo KS, Meredith IT, Worthley MI *et al*: **Diagnostic accuracy of adenosine stress cardiovascular magnetic resonance following acute ST-segment elevation myocardial infarction post primary angioplasty**. *Journal of Cardiovascular Magnetic Resonance* 2011, **13**:62.
31. Doesch C, Seeger A, Doering J, Herdeg C, Burgstahler C, Claussen CD, Gawaz M, Miller S, May AE: **Risk stratification by adenosine stress cardiac magnetic resonance in patients with coronary artery stenoses of intermediate angiographic severity**. *Jacc: Cardiovascular Imaging* 2009, **2**(4):424-433.
32. American College of Cardiology Foundation Task Force on Expert Consensus D, Hundley WG, Bluemke DA, Finn JP, Flamm SD, Fogel MA, Friedrich MG, Ho VB, Jerosch-Herold M, Kramer CM *et al*: **ACCF/ACR/AHA/NASCI/SCMR 2010 expert consensus document on cardiovascular magnetic resonance: a report of the American College of Cardiology Foundation Task Force on Expert Consensus Documents**. *Circulation* 2010, **121**(22):2462-2508.
33. Hoffmann U, Globits S, Schima W, Loewe C, Puig S, Oberhuber G, Frank H: **Usefulness of magnetic resonance imaging of cardiac and paracardiac masses**. *The American journal of cardiology* 2003, **92**(7):890-895.
34. Chan PG, Smith MP, Hauser TH, Yeon SB, Appelbaum E, Rofsky NM, Manning WJ: **Noncardiac pathology on clinical cardiac magnetic resonance imaging**. *JACC Cardiovascular imaging* 2009, **2**(8):980-986.
35. McKenna DA, Laxpati M, Colletti PM: **The prevalence of incidental findings at cardiac MRI**. *The open cardiovascular medicine journal* 2008, **2**:20-25.
36. Wyttenbach R, Medioni N, Santini P, Vock P, Szucs-Farkas Z: **Extracardiac findings detected by cardiac magnetic resonance imaging**. *Eur Radiol* 2012, **22**(6):1295-1302.
