# Supplementary material for: Formal consensus to identify clinically important changes in management resulting from the use of cardiovascular magnetic resonance (CMR) in patients who activate the primary percutaneous coronary intervention (PPCI) pathway
Source: BMJ Open. 2017 Jun 22;7(6):e014627. doi: 10.1136/bmjopen-2016-014627 (PMC5541580; doi:10.1136/bmjopen-2016-014627)
Supplement: Supplementary Appendix 3 [file bmjopen-2016-014627supp003.pdf]

Appendix 3

Modified survey (statements, supporting paragraphs and references)

Accurate assessment of infarct characteristics is important for risk stratification after PPCI. CMR can quantify in a single scan all cardiac markers relevant to PPCI outcomes, with high reproducibility and accuracy. CMR has high spatial and temporal resolution and is superior to echocardiography for measuring left ventricular volumes and ejection fraction. **[1]** Additionally, CMR markers that cannot be measured by echocardiography, such as infarct size, microvascular obstruction and myocardial salvage have been shown to have long term prognostic value. **[2-4]** Late gadolinium enhancement CMR has added prognostic value over echocardiography. **[5]**

1. <u>The following statements relate to the ability of CMR to identify patients who have a **poor** prognosis after PPCI.</u>

   a. CMR markers (e.g. impaired left ventricular function, large infarct size, microvascular obstruction) better identify patients with a poor prognosis after PPCI than markers based on echocardiography.

Strongly disagree	Moderately disagree	Neither agree nor disagree	Moderately agree	Strongly agree

   b. Better identification of patients with a poor prognosis after PPCI allows these patients to be followed up more appropriately and treated more aggressively

Strongly disagree	Moderately disagree	Neither agree nor disagree	Moderately agree	Strongly agree

   c. More appropriate follow up and more aggressive treatment in these patients is expected to lead to a reduced risk of MACE in the long term.

Strongly disagree	Moderately disagree	Neither agree nor disagree	Moderately agree	Strongly agree

   d. More appropriate follow up and more aggressive treatment in these patients is expected to lead to less NHS resource use in the long term.

Strongly disagree	Moderately disagree	Neither agree nor disagree	Moderately agree	Strongly agree

   e. The anticipated benefit (reduced risk of MACE and/or less NHS resource use) is likely to be sufficiently large to make CMR cost effective among the patients in whom it would be indicated.

Strongly disagree	Moderately disagree	Neither agree nor disagree	Moderately agree	Strongly agree

2. <u>The following statements relate to the ability of CMR to identify patients who have a **good** prognosis after PPCI.</u>

   a. CMR markers (e.g. normal left ventricular function, high myocardial salvage, no microvascular obstruction, no residual ischemia) better identify patients with a good prognosis after PPCI than markers based on echocardiography.

Strongly disagree	Moderately disagree	Neither agree nor disagree	Moderately agree	Strongly agree

   b. Better identification of patients with a good prognosis after PPCI allows these patients to be followed up less frequently.

Strongly disagree	Moderately disagree	Neither agree nor disagree	Moderately agree	Strongly agree

   c. Less frequent follow up in these patients is expected to lead to less NHS resource use in the long term.

Strongly disagree	Moderately disagree	Neither agree nor disagree	Moderately agree	Strongly agree

   d. The anticipated benefit (less NHS resource use) is likely to be sufficiently large to make CMR cost effective among the patients in whom it would be indicated.

Strongly disagree	Moderately disagree	Neither agree nor disagree	Moderately agree	Strongly agree

Out-of-hospital cardiac arrest (OHCA) affects about 60,000 people in the UK each year. Currently, hospital survival among patients alive on arrival is about 32%. Early identification of the cause of OHCA is essential to improve survival. Causes of OHCA include myocardial infarction (40%-90%) and inherited cardiomyopathies such as arrhythmogenic right ventricular cardiomyopathy (ARVC) and hypertrophic cardiomyopathy (HCM). Unlike echocardiography, CMR allows *in vivo* tissue characterisation, which differentiates scarring due to myocardial infarction from other causes of focal fibrosis (e.g. observed in non-ischaemic cardiomyopathies such as ARVC and HCM).**[6]** Late gadolinium enhancement on CMR can predict serious cardiac complications in patients with HCM (all-cause death, cardiac death and death from heart failure),**[7]** and identifies patients that may need more aggressive medical and device therapy (e.g. renin-aldosterone system inhibition for prevention of heart failure or implantable cardioverter defibrillator placement for primary prevention of sudden cardiac death.

3. <u>The following statements relate to the ability of CMR to identify the causes of OHCA in patients who undergo an emergency angiogram.</u>

   a. CMR better identifies the cause of OHCA (e.g. large myocardial infarction, ARVC, aberrant coronary arteries, HCM) than echocardiography.

Strongly disagree	Moderately disagree	Neither agree nor disagree	Moderately agree	Strongly agree

   b. Better identification of the cause of out-of-hospital cardiac arrest allows treatment to be optimised for these patients (e.g. defibrillator for primary arrhythmia or percutaneous coronary intervention) or their family members (e.g. genetic screening and counselling, primary prevention).

Strongly disagree	Moderately disagree	Neither agree nor disagree	Moderately agree	Strongly agree

   c. The ability to optimise treatment for these patients or family members is expected to lead to a reduced risk of MACE in the long term.

Strongly disagree	Moderately disagree	Neither agree nor disagree	Moderately agree	Strongly agree

   d. The ability to optimise treatment for these patients or family members is expected to lead to less NHS resource use in the long term.

Strongly disagree	Moderately disagree	Neither agree nor disagree	Moderately agree	Strongly agree

   e. The anticipated benefit (reduced risk of MACE and/or less NHS resource use) is likely to be sufficiently large to make CMR cost effective among the patients in whom it would be indicated.

Strongly disagree	Moderately disagree	Neither agree nor disagree	Moderately agree	Strongly agree

Ventricular septal defect (VSD) and left ventricular free wall rupture are rare complications of myocardial infarction, occurring in less than 1% of PPCI patients, usually within one week of the infarct. Mortality ranges between 50% and 87%. Diagnosis usually requires multi-modality imaging (echocardiography, ventriculography, computed tomography, CMR) before surgical repair. Because of its high spatial resolution, CMR can be used to clarify the detailed structure of these lesions. CMR accurately identifies the location, size and tissue margins of VSD and is useful for detecting apical defects, which are not easily identified by echocardiography. CMR measurements may also be used to determine the size of ventricular patch required to close the VSD, which avoids the inflation of a sizing balloon in friable infarcted tissue. **[8]** These features, together with other CMR markers of damage (e.g. infarct size, microvascular obstruction, left ventricular dysfunction) are useful for guiding optimal management of patients with post-infarct VSD.

4. <u>The following statements relate to the ability of CMR to identify patients with VSD after myocardial infarction.</u>

   a. CMR identifies the location and characteristics of post-infarct VSD better than echocardiography.

Strongly disagree		Moderately disagree		Neither agree nor disagree		Moderately agree		Strongly agree
○	○	○	○	○	○	○	○	○

   b. Better identification of the location and characteristics of post-infarct VSD guides the optimal management of these patients.

Strongly disagree		Moderately disagree		Neither agree nor disagree		Moderately agree		Strongly agree
○	○	○	○	○	○	○	○	○

   c. Optimal management of patients with post-infarct VSD is expected to lead to a reduced risk of MACE in the long term.

Strongly disagree		Moderately disagree		Neither agree nor disagree		Moderately agree		Strongly agree
○	○	○	○	○	○	○	○	○

   d. Optimal management of patients with post-infarct VSD is expected to lead to less NHS resource use in the long term.

Strongly disagree		Moderately disagree		Neither agree nor disagree		Moderately agree		Strongly agree
○	○	○	○	○	○	○	○	○

   e. The anticipated benefit (reduced risk of MACE and/or less NHS resource use) is likely to be sufficiently large to make CMR cost effective among the patients in whom it would be indicated.

Strongly disagree		Moderately disagree		Neither agree nor disagree		Moderately agree		Strongly agree
○	○	○	○	○	○	○	○	○

Between 5% and 12% of patients who have an emergency angiogram are found to have unobstructed coronary arteries. In these patients, the lack of an accurate diagnosis may result in inappropriate or unnecessary treatment or follow-up and poorer prognosis. **[9]** CMR facilitates differential diagnosis in patients with unobstructed coronary arteries on angiography, providing a definitive diagnosis (e.g. myocardial infarction, myocarditis, Takotsubo cardiomyopathy) in 65-90% of these patients. **[10, 11]** In patients with myocarditis, late gadolinium enhancement (LGE) on CMR may predict long term adverse outcomes. **[12]** In the context of myocardial infarction without an angiographic lesion, CMR can locate the culprit infarct-related artery in patients with spontaneous reperfusion or with distal embolization.

5. <u>The following statements relate to the ability of CMR to differentiate myocaradial infarction from other diagnoses in patients found to have unobstructed coronary arteries on emergency angiography.</u>

   a. Unlike echocardiography, CMR can provide a definitive ischaemic diagnosis (e.g. myocardial infarction with spontaneous reperfusion or distal embolization) or a non-ischaemic diagnosis (e.g. myocarditis, Takotsubo cardiomyopathy, aortic dissection) in patients with unobstructed coronary arteries on angiography.

Strongly disagree		Moderately disagree		Neither agree nor disagree		Moderately agree		Strongly agree
○	○	○	○	○	○	○	○	○

   b. A definitive diagnosis results in a patient treatment plan appropriate for that diagnosis.

Strongly disagree		Moderately disagree		Neither agree nor disagree		Moderately agree		Strongly agree
○	○	○	○	○	○	○	○	○

   c. A treatment plan appropriate for the diagnosis is expected to lead to a reduced risk of MACE in the long term.

Strongly disagree		Moderately disagree		Neither agree nor disagree		Moderately agree		Strongly agree
○	○	○	○	○	○	○	○	○

   d. A treatment plan appropriate for the definitive diagnosis is expected to lead to less NHS resource use in the long term.

Strongly disagree		Moderately disagree		Neither agree nor disagree		Moderately agree		Strongly agree
○	○	○	○	○	○	○	○	○

   e. The anticipated benefit (reduced risk of MACE and/or less NHS resource use) is likely to be sufficiently large to make CMR cost effective among the patients in whom it would be indicated.

Strongly disagree		Moderately disagree		Neither agree nor disagree		Moderately agree		Strongly agree
○	○	○	○	○	○	○	○	○

About 6% of heart attack patients subsequently die suddenly from a presumed cardiac cause. Current guidelines recommend the use of an implantable cardioverter defibrillator (ICD) to prevent sudden cardiac death in patients who have a low left ventricular ejection fraction (EF) after a heart attack. EF is used in clinical practice to make decisions about ICD implantation, but it has a low predictive value and many patients with an ICD will never benefit from it. **[13]** EF is most commonly measured by echocardiography, but CMR is now considered the gold standard for EF measurement because it is more reproducible than echocardiography. **[1]** CMR has been shown to be better than echocardiography for selecting patients for ICD implantation when strict EF thresholds are used to guide implantation. **[14]** The extent of myocardial scar characterised by late gadolinium enhancement (LGE) CMR may also be used to predict whether ICD implantation is appropriate in this patient group. **[15]** Furthermore, LGE CMR can guide placement of the left ventricular lead away from scarred myocardium, which results in a better clinical outcome after cardiac resynchronisation therapy (CRT). **[16]**

6. <u>The following statements relate to the ability of CMR to identify patients at high risk of sudden cardiac death after PPCI who would benefit most from an implantable cardiac device.</u>

   a. CMR identifies PPCI patients who are at high risk of sudden cardiac death better than echocardiography.

Strongly disagree	Moderately disagree	Neither agree nor disagree	Moderately agree	Strongly agree

   b. Better identification of PPCI patients at high risk of sudden cardiac death allows optimal patient selection for an implantable cardiac device (ICD or CRT).

Strongly disagree	Moderately disagree	Neither agree nor disagree	Moderately agree	Strongly agree

   c. Optimal patient selection for an implantable cardiac device is expected to lead to a reduced risk of MACE in these patients in the long term.

Strongly disagree	Moderately disagree	Neither agree nor disagree	Moderately agree	Strongly agree

   d. Optimal patient selection for an implantable cardiac device is expected to lead to less NHS resource use in the long term.

Strongly disagree	Moderately disagree	Neither agree nor disagree	Moderately agree	Strongly agree

   e. The anticipated benefit (reduced risk of MACE and/or less NHS resource use) is likely to be sufficiently large to make CMR cost effective among the patients in whom it would be indicated.

Strongly disagree	Moderately disagree	Neither agree nor disagree	Moderately agree	Strongly agree

Cardiac resynchronisation therapy (CRT), or biventricular pacing, uses a specialised pacemaker to improve the contraction of the left ventricle and the overall efficiency of the heart. It is used in patients with systolic ventricular dysfunction and heart failure. However, about 30% of patients who meet the inclusion criteria for CRT do not respond to it. **[17]** CMR assessment of mechanical dyssynchrony and myocardial scar provides additional value over echocardiography for identifying non responsive patients. **[18, 19]** Myocardial scar is an important feature of non-response to CRT **[20, 21]** and late gadolinium enhancement (LGE) CMR accurately differentiates between transmural, mid-myocardial, epicardial, and subendocardial scar.

7. <u>The following statements relate to the ability of CMR to identify patients who **would not** benefit from CRT after PPCI.</u>

   a. CMR identifies patients who would not benefit from CRT better than echocardiography.

Strongly disagree	Moderately disagree	Neither agree nor disagree	Moderately agree	Strongly agree

   b. The ability to identify patients who would not benefit from CRT would reduce CRT use in patients who do not need it.

Strongly disagree	Moderately disagree	Neither agree nor disagree	Moderately agree	Strongly agree

   c. Reducing CRT use in patients who do not need it is expected to lead to reduced risk of MACE in these patients in the long term.

Strongly disagree	Moderately disagree	Neither agree nor disagree	Moderately agree	Strongly agree

   d. Reducing CRT use in patients who do not need it is expected to lead to less NHS resource use in the long term.

Strongly disagree	Moderately disagree	Neither agree nor disagree	Moderately agree	Strongly agree

   e. The anticipated benefit (reduced risk of MACE and/or less NHS resource use) is likely to be sufficiently large to make CMR cost effective among the patients in whom it would be indicated.

Strongly disagree	Moderately disagree	Neither agree nor disagree	Moderately agree	Strongly agree

Between 40% and 65% of the patients who activate the PPCI pathway have multi-vessel disease. Adequate assessment of residual ischemia in non-culprit arteries post PPCI is important for effective management because this patient group has an adverse prognosis. Various techniques are currently used to assess the need for additional revascularisation, including stress echocardiography, single photon emission computed tomography (SPECT) and stress-perfusion CMR. Stress CMR has excellent prognostic value **[22]** and better diagnostic accuracy than SPECT **[23, 24]** or stress echocardiography **[25]** for detecting coronary artery disease causing significant regional myocardial ischaemia.

8. <u>The following statements relate to the ability of CMR to assess ischemia and viability in patients with multi-vessel disease.</u>

   a. CMR assesses ischemia and viability of the myocardium better than echocardiography.

Strongly disagree	Moderately disagree	Neither agree nor disagree	Moderately agree	Strongly agree

   b. Better assessment of ischemia and viability of the myocardium optimises the revascularisation strategy for patients with multi-vessel disease and avoids additional diagnostic tests.

Strongly disagree	Moderately disagree	Neither agree nor disagree	Moderately agree	Strongly agree

   c. The ability to optimise the revascularisation strategy for patients with multi-vessel disease is expected to lead to a reduced risk of MACE in the long term.

Strongly disagree	Moderately disagree	Neither agree nor disagree	Moderately agree	Strongly agree

   f. The ability to optimise the revascularisation strategy and avoid additional diagnostic tests in patients with multi-vessel disease is expected to lead to less NHS resource use in the long term.

Strongly disagree	Moderately disagree	Neither agree nor disagree	Moderately agree	Strongly agree

   d. The anticipated benefit (reduced risk of MACE and/or less NHS resource use) is likely to be sufficiently large to make CMR cost effective among the patients in whom it would be indicated.

Strongly disagree	Moderately disagree	Neither agree nor disagree	Moderately agree	Strongly agree

Left ventricular (LV) thrombus is a serious complication of acute myocardial infarction. It increases risk of thromboembolic events, particularly stroke. LV thrombus develops in up to 10% of patients with anterior wall infarction after PPCI. Although echocardiography is most commonly used to detect LV thrombus and to assess its shape and size, between 10-46% of echocardiograms are inconclusive. **[26]** Late gadolinium enhancement (LGE) CMR is considered the gold standard for detecting LV thrombus, because it detects thrombus based on tissue characteristics rather than anatomic appearance. Fewer thrombi are detected by contrast-echocardiography than by LGE CMR. **[27]** CMR has a higher sensitivity (88%) than contrast (61%) and noncontrast (<33%) echocardiography. **[27, 28]**

9. <u>The following statements relate to the ability of CMR to identify patients with post-infarct LV thrombus.</u>

    a. CMR identifies post-infarct LV thrombus better than echocardiography.

Strongly disagree	Moderately disagree	Neither agree nor disagree	Moderately agree	Strongly agree

    b. Better detection of post-infarct LV thrombus in PPCI patients allows more affected patients to be treated with anticoagulation therapy.

Strongly disagree	Moderately disagree	Neither agree nor disagree	Moderately agree	Strongly agree

    c. Treatment with anticoagulation therapy in patients with post-infarct LV thrombus is expected to lead to a reduced risk of MACE in the long term.

Strongly disagree	Moderately disagree	Neither agree nor disagree	Moderately agree	Strongly agree

    d. Treatment with anticoagulation therapy in patients with post-infarct LV thrombus is expected to lead to less NHS resource use in the long term.

Strongly disagree	Moderately disagree	Neither agree nor disagree	Moderately agree	Strongly agree

    e. The anticipated benefit (reduced risk of MACE and/or less NHS resource use) is likely to be sufficiently large to make CMR cost effective among the patients in whom it would be indicated.

Strongly disagree	Moderately disagree	Neither agree nor disagree	Moderately agree	Strongly agree

Compared with echocardiography, CMR provides versatile imaging planes, superior tissue contrast and advanced tissue characterisation, allowing a comprehensive assessment of cardiac anatomy, function and flow, and imaging of the great vessels (including venous return), the pericardium and suspected cardiac tumours. **[29]** As such, CMR can identify congenital coronary anomalies, cardiac masses, coronary artery aneurysms, valvular heart disease, thoracic aortic disease, etc. CMR can adequately differentiate benign from malignant tumours in the heart. **[30]** Unlike echocardiography, which provides limited imaging of mediastinal and extra-cardiac structures, CMR also images a substantial part of the thorax and abdomen in the field of view, which may potentially contain non-cardiac abnormalities. The prevalence of non-cardiac findings on CMR is up to 80% (with up to 30% of these representing potentially significant findings), depending on the characteristics of the population examined. **[31-33]**

10. The following statements relate to the ability of CMR to detect incidental cardiac and non-cardiac findings if offered routinely to patients who undergo an emergency angiogram.

    a. CMR identifies more incidental cardiac / non-cardiac findings than echocardiography.

Strongly disagree	Moderately disagree	Neither agree nor disagree	Moderately agree	Strongly agree

    b. Improved detection of potentially significant incidental findings allows affected patients to be investigated further and / or treated.

Strongly disagree	Moderately disagree	Neither agree nor disagree	Moderately agree	Strongly agree

    c. Further investigation and treatment is expected to reduce the risk of MACE / increase overall survival in affected patients in the long term.

Strongly disagree	Moderately disagree	Neither agree nor disagree	Moderately agree	Strongly agree

    d. Further investigation and treatment is expected to lead to less NHS resource use in the long term.

Strongly disagree	Moderately disagree	Neither agree nor disagree	Moderately agree	Strongly agree

    e. The anticipated benefit (reduced risk of MACE and/or less NHS resource use) is likely to be sufficiently large to make CMR cost effective among the patients who undergo an emergency angiogram.

Strongly disagree	Moderately disagree	Neither agree nor disagree	Moderately agree	Strongly agree

References

1. Dorosz JL, Lezotte DC, Weitzenkamp DA, Allen LA, Salcedo EE: **Performance of 3-dimensional echocardiography in measuring left ventricular volumes and ejection fraction: a systematic review and meta-analysis**. *J Am Coll Cardiol* 2012, **59**(20):1799-1808.
2. Eitel I, Desch S, de WS, Fuernau G, Gutberlet M, Schuler G, Thiele H: **Long-term prognostic value of myocardial salvage assessed by cardiovascular magnetic resonance in acute reperfused myocardial infarction**. *Heart* 2011, **97**(24):2038-2045.
3. Eitel I, Desch S, Fuernau G, Hildebrand L, Gutberlet M, Schuler G, Thiele H: **Prognostic significance and determinants of myocardial salvage assessed by cardiovascular magnetic resonance in acute reperfused myocardial infarction**. *J Am Coll Cardiol* 2010, **55**(22):2470-2479.
4. Klug G, Mayr A, Schenk S, Esterhammer R, Schocke M, Nocker M, Jaschke W, Pachinger O, Metzler B: **Prognostic value at 5 years of microvascular obstruction after acute myocardial infarction assessed by cardiovascular magnetic resonance**. *Journal of Cardiovascular Magnetic Resonance* 2012, **14**:46.
5. Wong TC, Piehler K, Puntil KS, Moguillansky D, Meier CG, Lacomis JM, Kellman P, Cook SC, Schwartzman DS, Simon MA *et al*: **Effectiveness of late gadolinium enhancement to improve outcomes prediction in patients referred for cardiovascular magnetic resonance after echocardiography**. *Journal of cardiovascular magnetic resonance : official journal of the Society for Cardiovascular Magnetic Resonance* 2013, **15**:6.
6. Parsai C, O'Hanlon R, Prasad SK, Mohiaddin RH: **Diagnostic and prognostic value of cardiovascular magnetic resonance in non-ischaemic cardiomyopathies**. *Journal of cardiovascular magnetic resonance : official journal of the Society for Cardiovascular Magnetic Resonance* 2012, **14**:54.
7. Green JJ, Berger JS, Kramer CM, Salerno M: **Prognostic value of late gadolinium enhancement in clinical outcomes for hypertrophic cardiomyopathy**. *JACC Cardiovascular imaging* 2012, **5**(4):370-377.
8. Artis NJ, Thomson J, Plein S, Greenwood JP: **Percutaneous closure of postinfarction ventricular septal defect: cardiac magnetic resonance-guided case selection and postprocedure evaluation**. *The Canadian journal of cardiology* 2011, **27**(6):869 e863-865.
9. Dokainish H, Pillai M, Murphy SA, DiBattiste PM, Schweiger MJ, Lotfi A, Morrow DA, Cannon CP, Braunwald E, Lakkis N: **Prognostic implications of elevated troponin in patients with suspected acute coronary syndrome but no critical epicardial coronary disease: a TACTICS-TIMI-18 substudy**. *J Am Coll Cardiol* 2005, **45**(1):19-24.
10. Assomull RG, Lyne JC, Keenan N, Gulati A, Bunce NH, Davies SW, Pennell DJ, Prasad SK: **The role of cardiovascular magnetic resonance in patients presenting with chest pain, raised troponin, and unobstructed coronary arteries**. *Eur Heart J* 2007, **28**(10):1242-1249.
11. Leurent G, Langella B, Fougerou C, Lentz PA, Larralde A, Bedossa M, Boulmier D, Le Breton H: **Diagnostic contributions of cardiac magnetic resonance imaging in patients presenting with elevated troponin, acute chest pain syndrome and unobstructed coronary arteries**. *Arch Cardiovasc Dis* 2011, **104**(3):161-170.
12. Grun S, Schumm J, Greulich S, Wagner A, Schneider S, Bruder O, Kispert EM, Hill S, Ong P, Klingel K *et al*: **Long-term follow-up of biopsy-proven viral myocarditis: predictors of mortality and incomplete recovery**. *J Am Coll Cardiol* 2012, **59**(18):1604-1615.
13. Moss AJ, Greenberg H, Case RB, Zareba W, Hall WJ, Brown MW, Daubert JP, McNitt S, Andrews ML, Elkin AD *et al*: **Long-term clinical course of patients after termination of ventricular tachyarrhythmia by an implanted defibrillator**. *Circulation* 2004, **110**(25):3760-3765.
14. Joshi SB, Connelly KA, Jimenez-Juan L, Hansen M, Kirpalani A, Dorian P, Mangat I, Al-Hesayen A, Crean AM, Wright GA *et al*: **Potential clinical impact of cardiovascular magnetic

resonance assessment of ejection fraction on eligibility for cardioverter defibrillator implantation. *Journal of cardiovascular magnetic resonance : official journal of the Society for Cardiovascular Magnetic Resonance* 2012, **14**:69.

15. Alexandre J, Saloux E, Dugue AE, Lebon A, Lemaitre A, Roule V, Labombarda F, Provost N, Gomes S, Scanu P *et al*: **Scar extent evaluated by late gadolinium enhancement CMR: a powerful predictor of long term appropriate ICD therapy in patients with coronary artery disease**. *Journal of cardiovascular magnetic resonance : official journal of the Society for Cardiovascular Magnetic Resonance* 2013, **15**:12.
16. Leyva F, Foley PW, Chalil S, Ratib K, Smith RE, Prinzen F, Auricchio A: **Cardiac resynchronization therapy guided by late gadolinium-enhancement cardiovascular magnetic resonance**. *Journal of cardiovascular magnetic resonance : official journal of the Society for Cardiovascular Magnetic Resonance* 2011, **13**:29.
17. Leclercq C, Kass DA: **Retiming the failing heart: principles and current clinical status of cardiac resynchronization**. *J Am Coll Cardiol* 2002, **39**(2):194-201.
18. Bilchick KC, Dimaano V, Wu KC, Helm RH, Weiss RG, Lima JA, Berger RD, Tomaselli GF, Bluemke DA, Halperin HR *et al*: **Cardiac magnetic resonance assessment of dyssynchrony and myocardial scar predicts function class improvement following cardiac resynchronization therapy**. *JACC Cardiovascular imaging* 2008, **1**(5):561-568.
19. Ellims AH, Pfluger H, Elsik M, Butler MJ, Hare JL, Taylor AJ: **Utility of cardiac magnetic resonance imaging, echocardiography and electrocardiography for the prediction of clinical response and long-term survival following cardiac resynchronisation therapy**. *Int J Cardiovasc Imaging* 2013, **29**(6):1303-1311.
20. Adelstein EC, Saba S: **Scar burden by myocardial perfusion imaging predicts echocardiographic response to cardiac resynchronization therapy in ischemic cardiomyopathy**. *Am Heart J* 2007, **153**(1):105-112.
21. White JA, Yee R, Yuan X, Krahn A, Skanes A, Parker M, Klein G, Drangova M: **Delayed enhancement magnetic resonance imaging predicts response to cardiac resynchronization therapy in patients with intraventricular dyssynchrony**. *J Am Coll Cardiol* 2006, **48**(10):1953-1960.
22. Lipinski MJ, McVey CM, Berger JS, Kramer CM, Salerno M: **Prognostic value of stress cardiac magnetic resonance imaging in patients with known or suspected coronary artery disease: a systematic review and meta-analysis**. *J Am Coll Cardiol* 2013, **62**(9):826-838.
23. Greenwood JP, Maredia N, Younger JF, Brown JM, Nixon J, Everett CC, Bijsterveld P, Ridgway JP, Radjenovic A, Dickinson CJ *et al*: **Cardiovascular magnetic resonance and single-photon emission computed tomography for diagnosis of coronary heart disease (CE-MARC): a prospective trial**. *Lancet* 2012, **379**(9814):453-460.
24. Schwitter J, Wacker CM, Wilke N, Al-Saadi N, Sauer E, Huettle K, Schonberg SO, Debl K, Strohm O, Ahlstrom H *et al*: **Superior diagnostic performance of perfusion-cardiovascular magnetic resonance versus SPECT to detect coronary artery disease: The secondary endpoints of the multicenter multivendor MR-IMPACT II (Magnetic Resonance Imaging for Myocardial Perfusion Assessment in Coronary Artery Disease Trial)**. *Journal of cardiovascular magnetic resonance : official journal of the Society for Cardiovascular Magnetic Resonance* 2012, **14**:61.
25. Nagel E, Lehmkuhl HB, Bocksch W, Klein C, Vogel U, Frantz E, Ellmer A, Dreysse S, Fleck E: **Noninvasive diagnosis of ischemia-induced wall motion abnormalities with the use of high-dose dobutamine stress MRI: comparison with dobutamine stress echocardiography**. *Circulation* 1999, **99**(6):763-770.
26. Delewi R, Zijlstra F, Piek JJ: **Left ventricular thrombus formation after acute myocardial infarction**. *Heart* 2012, **98**(23):1743-1749.
27. Weinsaft JW, Kim RJ, Ross M, Krauser D, Manoushagian S, LaBounty TM, Cham MD, Min JK, Healy K, Wang Y *et al*: **Contrast-enhanced anatomic imaging as compared to contrast-**

enhanced tissue characterization for detection of left ventricular thrombus. *JACC Cardiovascular imaging* 2009, **2**(8):969-979.

28. Srichai MB, Junor C, Rodriguez LL, Stillman AE, Grimm RA, Lieber ML, Weaver JA, Smedira NG, White RD: **Clinical, imaging, and pathological characteristics of left ventricular thrombus: a comparison of contrast-enhanced magnetic resonance imaging, transthoracic echocardiography, and transesophageal echocardiography with surgical or pathological validation**. *Am Heart J* 2006, **152**(1):75-84.
29. American College of Cardiology Foundation Task Force on Expert Consensus D, Hundley WG, Bluemke DA, Finn JP, Flamm SD, Fogel MA, Friedrich MG, Ho VB, Jerosch-Herold M, Kramer CM *et al*: **ACCF/ACR/AHA/NASCI/SCMR 2010 expert consensus document on cardiovascular magnetic resonance: a report of the American College of Cardiology Foundation Task Force on Expert Consensus Documents**. *Circulation* 2010, **121**(22):2462-2508.
30. Hoffmann U, Globits S, Schima W, Loewe C, Puig S, Oberhuber G, Frank H: **Usefulness of magnetic resonance imaging of cardiac and paracardiac masses**. *The American journal of cardiology* 2003, **92**(7):890-895.
31. Chan PG, Smith MP, Hauser TH, Yeon SB, Appelbaum E, Rofsky NM, Manning WJ: **Noncardiac pathology on clinical cardiac magnetic resonance imaging**. *JACC Cardiovascular imaging* 2009, **2**(8):980-986.
32. McKenna DA, Laxpati M, Colletti PM: **The prevalence of incidental findings at cardiac MRI**. *The open cardiovascular medicine journal* 2008, **2**:20-25.
33. Wyttenbach R, Medioni N, Santini P, Vock P, Szucs-Farkas Z: **Extracardiac findings detected by cardiac magnetic resonance imaging**. *Eur Radiol* 2012, **22**(6):1295-1302.
