# Supplementary material for: Formal consensus to identify clinically important changes in management resulting from the use of cardiovascular magnetic resonance (CMR) in patients who activate the primary percutaneous coronary intervention (PPCI) pathway
Source: BMJ Open. 2017 Jun 22;7(6):e014627. doi: 10.1136/bmjopen-2016-014627 (PMC5541580; doi:10.1136/bmjopen-2016-014627)

Appendix 4

Median and interquartile (IQ) range for the 12 statements in the first survey (n=7). Boxes represent the median and IQ range and the whiskers represent the range. Dots represent extreme values. Statement 3, 5 and 9 were considered to be in consensus (median score of ≥7and IQ range of 6-9).

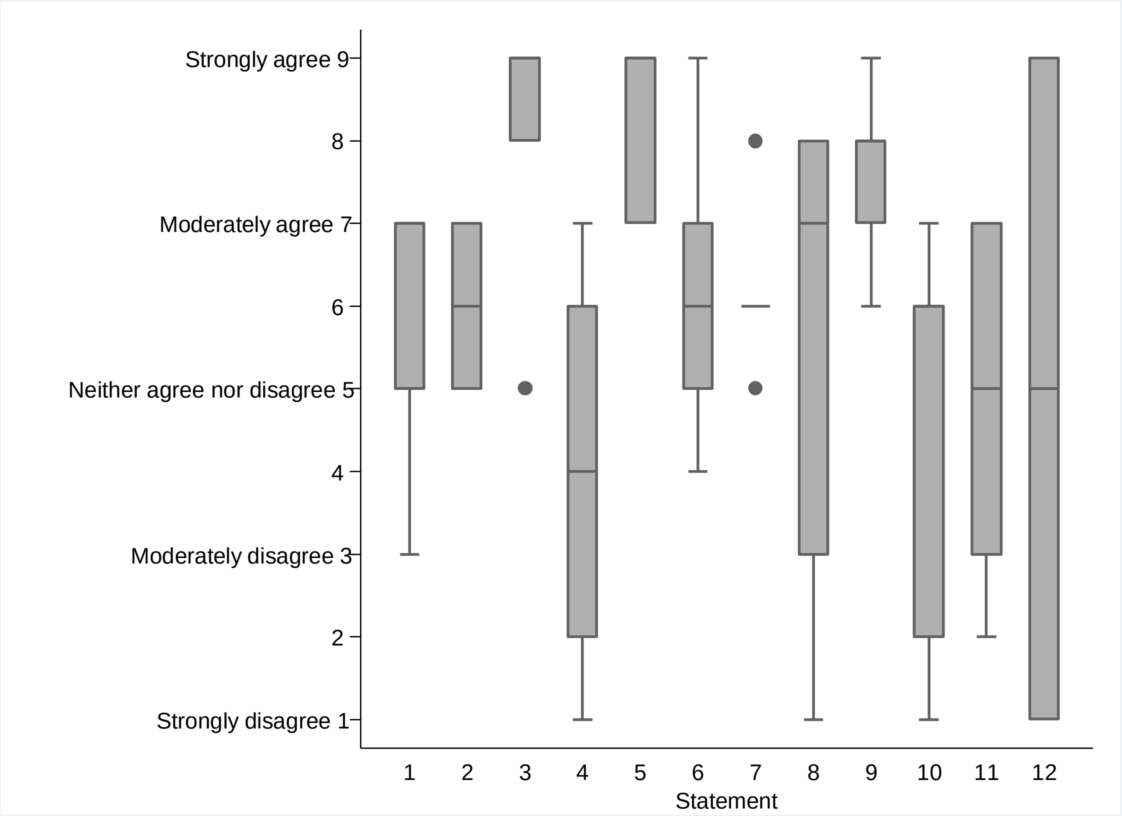

Supplement: Supplementary Appendix 4 [file bmjopen-2016-014627supp004.pdf]
