# Supplementary material for: Formal consensus to identify clinically important changes in management resulting from the use of cardiovascular magnetic resonance (CMR) in patients who activate the primary percutaneous coronary intervention (PPCI) pathway
Source: BMJ Open. 2017 Jun 22;7(6):e014627. doi: 10.1136/bmjopen-2016-014627 (PMC5541580; doi:10.1136/bmjopen-2016-014627)

BMJ Open

Formal consensus to identify clinically important changes in management resulting from cardiovascular magnetic resonance imaging (CMR) patients who activate the primary percutaneous coronary intervention (PPCI) pathway

For peer review only

Journal:	*BMJ Open*
Manuscript ID	bmjopen-2016-014627
Article Type:	Research
Date Submitted by the Author:	12-Oct-2016
Complete List of Authors:	Pufulete, Maria; University of Bristol,<br>Brierley, Rachel; University of Bristol<br>Bucciarelli Ducci, Chiara; University of Bristol<br>Greenwood, John; Leeds General Infirmary, Cardiology<br>Dorman, Stephen; University Hospitals Bristol NHS Foundation Trust<br>Anderson, Richard; University Hospital of Wales, Cardiology<br>Harris, Jessica; University of Bristol School of Clinical Science<br>MacAlindon, Elisa; New Cross Hospital<br>rogers, chris; university of bristol, Clinical Science South Bristol<br>Reeves, Barnaby; University of Bristol, Bristol Heart Institute
**Primary Subject Heading**:	Cardiovascular medicine
Secondary Subject Heading:	Diagnostics, Health services research, Medical management, Radiology and imaging, Research methods
Keywords:	primary percutaneous coronary intervention, systematic review, formal consensus, Cardiovascular magnetic resonance imaging

SCHOLARONE™
Manuscripts

Formal consensus to identify clinically important changes in management resulting from cardiovascular magnetic resonance imaging (CMR) patients who activate the primary percutaneous coronary intervention (PPCI) pathway

Maria Pufulete[1], Rachel Brierley[1], Chiara Bucciarelli-Ducci[2], John P. Greenwood[3], Stephen Dorman[2], Richard A Anderson[4], Jessica Harris[1], Elisa McAlindon[5], Chris A Rogers[1], Barnaby C Reeves[1]*

[1] Clinical Trials and Evaluation Unit, University of Bristol, Level 7, Bristol Royal Infirmary, Queen's Building, Bristol, United Kingdom.

[2] NIHR Bristol Cardiovascular Research Unit, Bristol Heart Institute, University Hospitals Bristol NHS Foundation Trust, United Kingdom.

[3] Multidisciplinary Cardiovascular Research Centre and Leeds Institute of Cardiovascular and Metabolic Medicine, University of Leeds, United Kingdom.

[4] University Hospitals of Wales, Heath Park, Cardiff, United Kingdom.

[5] Department of Cardiology, New Cross Hospital, Wolverhampton, United Kingdom.

[*] Correspondence to: Maria Pufulete, Clinical Trials and Evaluation Unit, University of Bristol, Level 7, Bristol Royal Infirmary, Queen's Building, Bristol, United Kingdom. Tel. +44 117 3422526. Fax. +44 117 3423288. Email: maria.pufulete@bristol.ac.uk

Abbreviations

ACS, acute coronary syndrome; CI, confidence interval; CMR, cardiovascular magnetic resonance imaging; IQ, interquartile range; IS, infarct size; LV, left ventricular; MACE, major adverse cardiovascular events; MI, myocardial infarction; MVD, multi-vessel disease; MVO, microvascular obstruction; NSTEMI, non ST-elevation myocardial infarction; OHCA, out of hospital cardiac arrest; PPCI, primary percutaneous coronary intervention; SPECT, single photon emission computed tomography; STEMI, ST-elevation myocardial infarction.

For peer review only - http://bmjopen.bmj.com/site/about/guidelines.xhtml

Key words: Cardiovascular magnetic resonance imaging, primary percutaneous coronary intervention, systematic review, formal consensus.

Word count: approx. 3142 (not including abstract, strengths and limitations, contributions, figs and tables or titles)

Number of tables: 3

Number of figures: 2

For peer review only

Abstract

Objective

To define important changes in management arising from the use of cardiovascular magnetic resonance (CMR) imaging in patients who activate the primary percutaneous coronary intervention (PPCI) pathway.

Design

Formal consensus study using literature review and cardiologist expert opinion to formulate consensus statements and setting up a consensus panel to review the statements (by completing a web-based survey, attending a face-to-face meeting to discuss survey results and modify the survey to reflect group discussion, and completing the modified survey to determine which statements were in consensus).

Participants

Formulation of consensus statements: four cardiologists (two CMR, two interventional) and six non-clinical researchers. Formal consensus: seven cardiologists (two CMR, three interventional, one echocardiography and one heart failure). Forty nine additional cardiologists completed the modified survey.

Results

Thirty seven draft statements describing changes in management following CMR were generated; these were condensed into 12 statements and reviewed through the formal consensus process. Three of 12 statements were classified in consensus in the first survey; these related to the role of CMR in: identifying the cause of out-of-hospital cardiac arrest; providing a definitive diagnosis in patients found to have unobstructed arteries on angiography; and identifying patients with left ventricular (LV) thrombus. Two additional statements were in consensus in the modified survey, relating to the ability of CMR to: identify patients who have a poor prognosis after PPCI; and assess ischemia and viability in patients with multi-vessel disease.

Conclusion

For peer review only - http://bmjopen.bmj.com/site/about/guidelines.xhtml

There was consensus that CMR leads to clinically important changes in management in five subgroups of patients who activate the PPCI pathway.

For peer review only

For peer review only - http://bmjopen.bmj.com/site/about/guidelines.xhtml

Strengths and limitations of this study

- We used formal consensus methods to identify potentially important changes in management arising from the use of cardiac magnetic resonance imaging (CMR) in patients who activate the PPCI pathway.
- We identified five patient subgroups in this population in which CMR leads to clinically important changes in management.
- We did not include different stakeholder groups in the consensus panel as the technical wording of the statements would not have been easily understood by non-cardiologists.
- The study did not consider other patient groups that may benefit from CMR (e.g. those with non ST-elevation myocardial infarction (NSTEMI) or stable coronary artery disease.

For peer review only

Introduction

There has been rapid uptake of cardiovascular magnetic resonance (CMR) imaging in the UK, with a 44% increase in the number of scans per centre (from 557 to 802) between 2008 and 2010 [1]. Despite the rapid increase in the number of scans undertaken there is limited evidence about the impact of CMR on long term prognosis or treatment decisions. CMR is increasingly being used for patients with acute coronary syndrome (ACS) who activate the primary percutaneous coronary intervention (PPCI) pathway, but it is not clear how CMR influences clinical management in this population.

We conducted a study to determine the feasibility of setting up a prospective registry using routine data linkage to assess the clinical and cost-effectiveness of CMR in patients who activate the PPCI pathway [2]. A long term objective of the registry is to compare the incidence of major adverse cardiovascular events (MACE) in patients who do or do not have CMR after the index event. However, given the rare occurrence of such events in these patients, the study would have to accrue over 37,000 subjects to detect a clinically important reduction in the incidence of MACE with adequate power. Therefore, a key objective of the feasibility study was to define a primary composite outcome, acceptable to cardiologists and other stakeholders (e.g. clinical commissioners) as representing a clinically important change in management as a result of an eligible patient having had CMR (e.g. expected to prevent future MACE), that could be used for the registry in the medium term.

We used a formal consensus method based on the modified nominal group technique [3] to identify important changes in management (and the specific patient subgroups these changes in management relate to) that can be used to define the composite outcome. Formal consensus is a method that combines both research evidence and expert opinion. We used this approach because we knew from a preliminary scoping of the literature that there were few studies that reported the impact of CMR on patient management. There were three components to the consensus process: (i) a literature review to identify evidence about the use of CMR in patients who activate the PPCI pathway; (ii) formulation of statements about how CMR changes patient management using research evidence and

For peer review only - http://bmjopen.bmj.com/site/about/guidelines.xhtml

expert opinion; and (iii) the formal consensus involving an independent panel of cardiologists using surveys and discussion to reach consensus.

Methods

Literature review and formulation of the consensus statements

An initial working group was convened including two cardiologists with CMR expertise, two interventional cardiologists from sites participating in the feasibility study, one cardiac network director, two methodologists, two statisticians, one health economist and the study manager. We conducted a literature review to search for studies reporting the impact of CMR on prognosis, patient management, and risk stratification in the population of patients who activate the PPCI pathway. We searched without restriction by study design or search terms related to outcomes, so that we could determine the full extent of the literature in this area and identify all studies that used CMR in our population. The search was conducted on Medline, EMBASE, The Cochrane Library, ISI Web of Science (Citations Index and Proceedings) and BIOSIS. The following search terms were used, both as free text and Medical Subject Headings where possible: ‘acute coronary syndrome’, ‘myocardial infarction’, ‘angioplasty’, ‘percutaneous coronary intervention’, ‘cardiovascular magnetic resonance imaging’. We applied no restriction on publication date or language.

Draft statements were generated independently by three of the cardiologists (based on clinical expertise) and by one methodologist (based on evidence from the systematic literature search). Three members of the working group (study manager, systematic reviewer and cardiologist with CMR expertise) collated the statements, organised them according to patient subgroup, and standardised the wording of each statement. A one day meeting was organised for all members of the working group to consider the relevance, format and wording of each statement.

Survey design

For peer review only - http://bmjopen.bmj.com/site/about/guidelines.xhtml

For peer review only

Statements and supporting paragraphs were worded in a consistent manner and collated in the form of a web-based survey (SurveyMonkey, Palo Alto, California, USA; **Appendix 1**). The survey included an introductory page explaining the purpose and layout of the survey and instructions about how to complete it. Each statement followed with its supporting paragraph containing links to PDF references. Each statement was accompanied by a 9 point Likert scale asking the respondent to indicate whether he/she agreed with the statement or not (with 1 indicating "completely disagree" and 9 "completely agree"). There was also a free text box for each statement for respondents to comment and justify their score.

Establishing the expert panel

Clinicians in the working group identified consultant cardiologists with CMR, interventional, echocardiography, electrophysiology and heart failure expertise from across the UK. The cardiologists were invited by email to form an expert consensus panel.

Expert panel: completion of first survey

The expert panel completed the survey independently. Responses were collated and analysed by members of the working group.

Expert panel: face-to-face meeting

The expert panel attended a face-to-face meeting, chaired by a non-cardiology clinician experienced in facilitating formal consensus panels. The meeting was also attended by non-clinical members of the working group, whose role was to introduce the study, describe the structure of the formal consensus process, provide study related information and take minutes of the meeting. The expert panel discussed each statement and anonymised responses to the first survey in turn and agreed on modifications to the survey.

For peer review only - http://bmjopen.bmj.com/site/about/guidelines.xhtml

Expert panel: completion of the modified survey

The survey was modified by members of the working group as agreed in the face-to-face meeting (**Appendix 2**). The expert panel completed the modified survey independently and rated the statements a second time.

Extension of survey to other UK cardiologists

The survey was extended to other UK cardiologists through the British Cardiovascular Society, which advertised the survey in their monthly newsletter to members (over two consecutive months).

Criteria for consensus

Data for each statement are shown as median and interquartile (IQ) range. A median score of ≥and IQ range 6-9 was considered to be in agreement, or consensus, that the change in management described by the statement was clinically important. These statements were used to identify the patient subgroups perceived to benefit from CMR and define the treatment / process outcome that constitutes a definitive management change as a result of having CMR. A median score of ≤3 and IQ range 1-3 was considered to be in consensus that the statement did not constitute a clinically important change in management. Data were analysed using Stata/IC (V14, StataCorp LP, Texas, USA).

Results

Literature review and formulation of consensus statements

Thirty seven draft statements were generated by the three cardiologists and one methodologist. The literature search identified a total of 171 studies reporting the use of CMR in patients with acute myocardial infarction (MI) who activated the PPCI pathway (**Appendix 3**). There were no studies that directly compared groups of patients having CMR or not with respect to patient management or clinical outcomes in this population. Statements relating to the same patient subgroup/condition (e.g. patients at risk of

For peer review only - http://bmjopen.bmj.com/site/about/guidelines.xhtml

complications after PPCI who develop left ventricular thrombus) were condensed into one statement. Participants at the working group combined some statements to avoid repetition and clarified the care pathway without magnetic resonance imaging compared to which the additional benefit of CMR was anticipated, e.g. alternative imaging modalities such as echocardiography or single-photon emission computed tomography (SPECT)). This process resulted in twelve statements describing changes in management relating to six patient subgroups (Table 1):

i. Patients with poor prognosis or at risk of complications after PPCI (statements 1, 4, 6, 7 and 9);
ii. Patients with good prognosis after PPCI who could be discharged earlier and followed up less often (statement 2);
iii. Patients with multi-vessel disease (MVD) (statement 8 and 10);
iv. Patients with unobstructed arteries on angiography (statement 5);
v. Patients with out-of-hospital-cardiac arrest (OHCA) (statement 3);
vi. Patients with incidental cardiac and extra-cardiac findings (statements 11 and 12).

Table 1 Consensus statements (first survey).

Statement		Patient subgroup
1	Compared with echocardiography, CMR after PPCI allows patients with CMR markers that indicate poor prognosis (e.g. impaired left ventricular function, large infarct size, microvascular obstruction) to be followed up more appropriately and undergo more aggressive medical therapy for secondary prevention.	Poor prognosis / risk of complications after PPCI
2	Compared with echocardiography, CMR after PPCI allows patients with CMR markers that indicate good prognosis (e.g. normal left ventricular function, high myocardial salvage, no microvascular obstruction, no residual ischemia) to be discharged earlier and followed up less frequently.	Good prognosis after PPCI
3	Compared with echocardiography, CMR better identifies the cause of out of hospital cardiac arrest (e.g. large myocardial infarction, arrhythmogenic right ventricular cardiomyopathy, aberrant coronary	Out of hospital cardiac arrest

For peer review only - http://bmjopen.bmj.com/site/about/guidelines.xhtml

Statement		Patient subgroup
	arteries, hypertrophic cardiomyopathy) to optimise further treatment for the patient (e.g. defibrillator for primary arrhythmia or percutaneous coronary intervention) or family members.	
4	CMR after PPCI identifies patients at high risk of having ventricular septal defect or impending cardiac rupture, who may require ventricular patch or other urgent cardiac surgery, and guides the optimal management of these patients.	Poor prognosis / risk of complications after PPCI
5	In patients with a “normal” (unobstructed) coronary angiogram, CMR can differentiate patients who have had a myocardial infarction with spontaneous reperfusion or distal embolization from patients with a non-ischaemic diagnosis (e.g. myocarditis, Takotsubo cardiomyopathy, aortic dissection), resulting in a patient treatment plan appropriate for the definitive diagnosis.	Unobstructed arteries on angiography
6	Compared with echocardiography, CMR after PPCI better identifies patients at high risk of sudden cardiac death who would benefit most from an implantable cardiac device (e.g. implantable cardioverter defibrillator or cardiac resynchronisation therapy).	Poor prognosis / risk of complications after PPCI
7	Compared with echocardiography, CMR after PPCI better identifies patients who would not benefit from cardiac resynchronisation therapy.	Poor prognosis / risk of complications after PPCI
8	Compared with stress echocardiography or single photon emission computed tomography (SPECT), CMR after PPCI in patients with multi-vessel disease better assesses ischaemia and viability of the myocardium to optimise the revascularisation strategy for the patient and avoid additional diagnostic tests.	Multi-vessel disease
9	Compared with echocardiography, CMR after PPCI better identifies patients with left ventricular (LV) thrombus for treatment with anticoagulation therapy.	Poor prognosis / risk of complications after PPCI
10	Compared with other imaging modalities, in patients with multi-vessel disease, CMR after PPCI better identifies the artery that caused the myocardial infarction and guides the subsequent treatment plan in relation to additional revascularisation (percutaneous coronary intervention, or coronary artery bypass graft surgery).	Multi-vessel disease
11	Compared with echocardiography, CMR after PPCI better identifies incidental cardiac findings that may need further investigation and	Incidental cardiac

For peer review only

Statement		Patient subgroup
	treatment.	findings
12	Compared with echocardiography, CMR after PPCI identifies significant incidental non-cardiac findings (e.g. oesophageal and lung tumours, pulmonary embolus, aortic aneurysm) that may need further investigation and treatment.	Incidental non-cardiac findings

The expert panel

Nineteen consultant cardiologists were invited to participate in the consensus process. Seven cardiologists (37% of those invited) agreed to participate. Of these, two had CMR, three had interventional, one had echocardiography and one had heart failure expertise. All seven cardiologists completed the first survey independently.

First survey

For peer review only

Figure shows the distributions of responses for the first survey. There was consensus for three of the 12 statements (25%) that the change in management was clinically important: statement 3, relating to the ability of CMR to identify the cause of OHCA and therefore optimise treatment for the patient; statement 5, relating to the ability of CMR to provide a definitive diagnosis in patients found to have unobstructed arteries on angiography; and statement 9, relating to the ability of CMR to identify patients with left ventricular (LV) thrombus and initiate treatment with anticoagulation therapy. There was no consensus for any statement that the change in management was not clinically important.

For peer review only

For peer review only - http://bmjopen.bmj.com/site/about/guidelines.xhtml

Figure 1 Median and interquartile (IQ) range for the 12 statements in the first survey (n=7). Boxes represent the median and IQ range and the whiskers represent the range. Dots represent extreme values. Statement 3, 5 and 9 were considered to be in consensus (median score of ≥7and IQ range of 6-9).

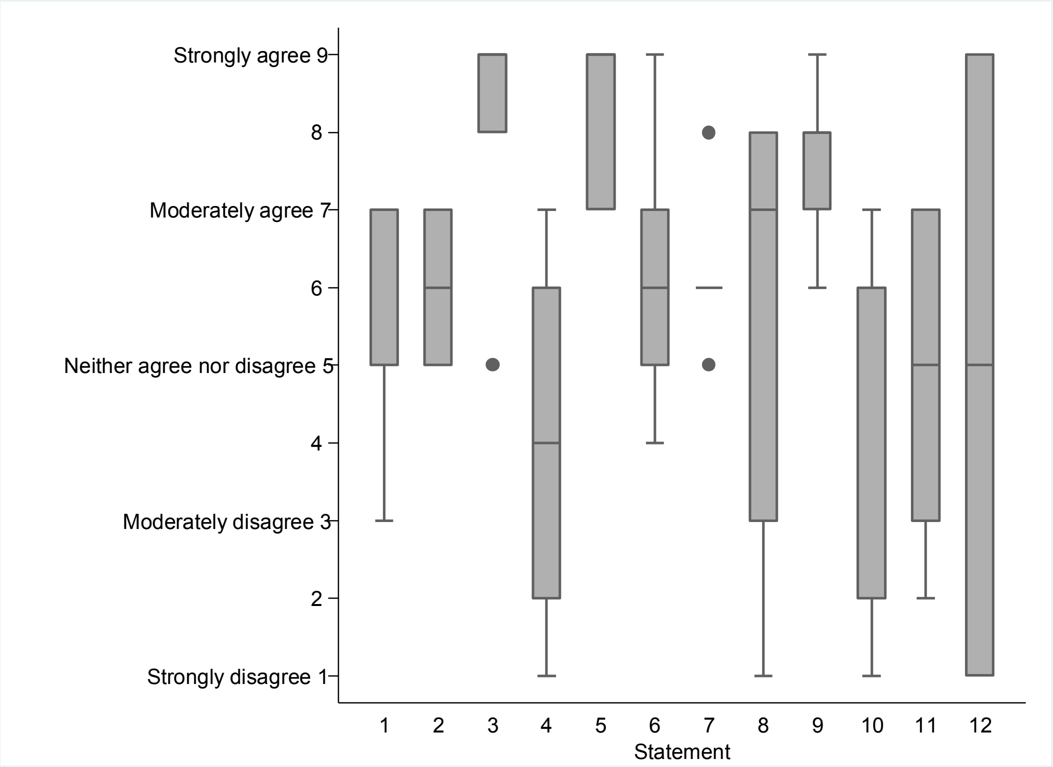

Examples of respondents' comments to the first survey regarding individual statements are shown in

view only

Table 2. Consensus was sought on the importance of the change in management and most respondents commented on this issue. However, some respondents also considered other factors when rating statements, for example, the quality of the supporting evidence, the proportion of patients likely to benefit, the ability of the NHS to provide a service in line with the statement, and whether the cost of CMR justified the perceived benefit.

For peer review only

For peer review only - http://bmjopen.bmj.com/site/about/guidelines.xhtml

Table 2 Examples of respondents' comments for three statements in consensus (3, 5 and 9) and three statements not in consensus (1, 2 and 8).

Statement 3	**Importance of management change**<br>*"CMR differentiates causes of cardiomyopathy (IHD vs not). We use it regularly in patients post out-of-hospital cardiac arrest."*<br>*"I have seen many cases of out-of-hospital cardiac arrest in which the use of CMR has made a diagnosis or significantly altered the diagnosis. Diagnostic refinement in the light of CMR findings also frequently results in changes in drug or device treatment, and often highlights the need for family screening. The improved anatomical, morphological, and tissue characterisation that CMR allows drives the benefit of CMR over echo and allows better identification of the cause of out-of-hospital arrest."*<br>**Quality of supporting evidence**<br>*"I don't think it has been proved that clinical outcomes are better with a CMR strategy."*<br>*"CMR is unlikely to alter the immediate management of these patients. However if the cause is unclear after echo and enzymes then CMR is helpful but I am not aware of any studies specifically assessing this."*
Statement 5	**Importance of management change**<br>*"Agree. Unobstructed coronaries with elevated troponin need diagnostic resolution and CMR is helpful."*<br>*"These patients are difficult to manage and often given incorrect diagnosis and especially different theories by different doctors during same admission."*<br>*"I have direct experience of the benefit of CMR in this area. Therefore, I strongly agree with statement 5. CMR with LGE is especially useful and I have seen many instances when CMR after acute MI with "normal" coronary arteries has helped diagnosis, differentiating between distal vessel occlusion, LV clot, myocarditis and Takotsubo cardiomyopathy. The results of CMR in this patient group have also directly affected my management of this group of patients including drugs used and length of stay."*<br>*"MRI is clinically useful in this situation in my experience and occasionally makes a very useful change to management."*<br>**Quality of supporting evidence**<br>*"Unfortunately again no RCT to indicate benefit of CMR."*
Statement 9	**Importance of management change**<br>*"Thrombus is poorly assessed by echo with many inconclusive reports in my experience locally."*<br>*"I strongly agree with this statement as I have experience of many patients in which echo demonstrated no LV thrombus that was subsequently found on CMR. This personal experience correlates with the studies quoted in the text of the statement."*<br>**Quality of supporting evidence**<br>*"This statement is true but whether this leads to better patient outcomes is uncertain."*
Statement 1	**Importance of management change**<br>*"All patients who have suffered an acute MI and subsequently undergone PPCI should receive aggressive secondary prevention. Thus, even though CMR can help refine*

For peer review only - http://bmjopen.bmj.com/site/about/guidelines.xhtml

	prognosis I do not feel that this additional information would lead to any significant changes to prescribing for secondary prevention. Enhanced confidence in CMR findings (compared to echo) and refinement of prognosis with respect to infarct size, MVO and MSI may help physicians discharge/follow up patients more appropriately."<br>*"All patients should have aggressive secondary prevention."*<br>**Quality of supporting evidence**<br>*"Whilst I agree with the evidence presented, there is no evidence to support the assertion the CMR findings lead to better outcomes for patients as there have been no trials assessing this."*<br>*"I don't think it has been proved that CMR compared with echo leads to improved patient outcomes."*<br>*"While MVO and MSI are markers of prognosis, LV systolic function remains the most important prognostic factor, which can be assessed with echo. We need interventions based on CMR parameters which improve prognosis."*<br>**Cost of CMR in relation to perceived benefit**<br>*"Agree but not sure current restricted availability and high cost and only modest anticipated change in clinical action justifies wholesale change from echo that also predicts risk well."*
Statement 2	**Importance of management change**<br>*"PPCI patients are already discharged early and often have little follow up."*<br>*"CMR has more reproducible results than echo for measuring cardiac function. When this reproducibility is combined with additional measurements that can further indicate good prognosis this should aid physicians' confidence in earlier discharge and appropriate timing of follow up."*<br>*"Also agree, but if good LV on echo and no clinical heart failure then discharge is safe. Unaware if that can be accelerated further by CMR."*<br>**Quality of supporting evidence**<br>*"I don't think it has been proved that clinical outcomes are better with a CMR strategy."*<br>*"Although this could be used in clinical practice there are no studies that I am aware that have assessed such a strategy."*
Statement 8	**Importance of management change**<br>*"I'd accept perfusion scanning or DSE as adequate tests for ischaemia and would really only specifically request CMR if there were additional diagnostic questions."*<br>*"Total revascularisation at one sitting with FFR guidance may render this unnecessary."*<br>*"Stress CMR in my experience is better than SPECT or stress echo. This is because the improved prognostic and diagnostic accuracy helps physicians manage, with confidence, non-significant coronary disease medically rather than invasively. There are also additional benefits of CMR, for example definition of scar for CRT implant or for VT ablation. It can be helpful to "archive" this information for latter use if the patient is going to receive a device such as an ICD that may preclude latter CMR scanning."*<br>**Quality of supporting evidence**<br>*"Evidence shows improved diagnostic accuracy compared to SPECT and DSE, but not*

For peer review only - http://bmjopen.bmj.com/site/about/guidelines.xhtml

	in this specific cohort." *"The evidence for ischaemia testing in the PPCI era does not really exist. There are no studies comparing MRI in this context with other modalities and definitely no RCT comparing CMR vs another modality."*

CMR: cardiovascular magnetic resonance imaging; DSE: dobutamine stress echocardiography; FFR: fractional flow reserve; IHD: ischaemic heart disease; LGE: late gadolinium enhancement; LV: left ventricular; MI: Myocardial infarction; MVO: microvascular obstruction; MSI: myocardial salvage index; PPCI: primary percutaneous coronary intervention; RCT: randomised controlled trial; SPECT: single photon emission computed tomography.

Face-to-face meeting

The face-to-face meeting was attended by six of the seven cardiologists and three non-clinical members of the initial working group. As a result of the face-to-face meeting, the survey was modified as follows:

i) The number of statements was reduced from 12 to 10. Statement 10 (relating to patients with MVD) was removed because cardiologists felt that this statement overlapped significantly with statement 8. Statements 11 and 12 (relating to incidental cardiac and non-cardiac findings) were combined into one statement.

ii) Respondents were asked to rate five aspects of each statement, organised hierarchically: (a) whether CMR is better than the comparator (e.g. echocardiography); (b) whether the information from CMR leads to a change in management; (c) whether the change in management is clinically important (i.e. likely to reduce risk of MACE in the long term); (d) whether the change in management is likely to reduce NHS costs in the long term; (e) whether the anticipated benefit was sufficiently large to make CMR cost effective among the patients in whom it would be indicated. Consensus was based on the distribution of responses to questions (a)-(c) of each statement [i.e. median ≥7, IQ range 6-9 for clinically important changes in management and median ≤ 3 and IQ range 1-3 for (a), (b) and (c)]. Responses to questions (a)-(c) were used because (a) was constructed to interrogate the respondent's appraisal of the evidence, (b) was constructed to determine whether the respondent believed the NHS service delivery changed as a result of CMR and (c) was constructed to determine whether the respondent believed that any change recognised in (b) was clinically important.

For peer review only - http://bmjopen.bmj.com/site/about/guidelines.xhtml

Discussions in the meeting revealed that consideration of costs and cost-effectiveness for the NHS influenced some panel member's responses to the initial survey. Questions (d) and (e) were added to isolate these aspects of consideration and separate them from the question of change in management, which was the aim of the consensus process.

iii) The free text box in which respondents could justify their score was removed.

The modified survey is shown in Error! Reference source not found..

Table 3 Consensus statements (modified survey)

Statement	
1	The following statements relate to the ability of CMR to identify patients who have a <u>poor</u> prognosis after PPCI.<br>a. CMR markers (e.g. impaired left ventricular function, large infarct size, microvascular obstruction) better identify patients with a poor prognosis after PPCI than markers based on echocardiography.<br>b. Better identification of patients with a poor prognosis after PPCI allows these patients to be followed up more appropriately and treated more aggressively.<br>c. More appropriate follow up and more aggressive treatment in these patients is expected to lead to a reduced risk of MACE in the long term.
2	The following statements relate to the ability of CMR to identify patients who have a <u>good</u> prognosis after PPCI.<br>a. CMR markers (e.g. normal left ventricular function, high myocardial salvage, no microvascular obstruction, no residual ischemia) better identify patients with a good prognosis after PPCI than markers based on echocardiography.<br>b. Better identification of patients with a good prognosis after PPCI allows these patients to be followed up less frequently.<br>c. Less frequent follow up in these patients is expected to lead to less NHS resource use in the long term.
3	The following statements relate to the ability of CMR to identify the causes of OHCA in patients who undergo an emergency angiogram.<br>a. CMR better identifies the cause of OHCA (e.g. large myocardial infarction, ARVC, aberrant coronary arteries, HCM) than echocardiography.<br>b. Better identification of the cause of out-of-hospital cardiac arrest allows treatment to be optimised for these patients (e.g. defibrillator for primary arrhythmia or percutaneous coronary intervention) or their family members (e.g. genetic screening and counselling, primary prevention).<br>c. The ability to optimise treatment for these patients or family members is expected to lead to a reduced risk of MACE in the long term.
4	The following statements relate to the ability of CMR to identify patients with VSD after myocardial infarction.<br>a. CMR identifies the location and characteristics of post-infarct VSD better than echocardiography.<br>b. Better identification of the location and characteristics of post-infarct VSD guides the optimal management of these patients.<br>c. Optimal management of patients with post-infarct VSD is expected to lead to a reduced risk of MACE in the long term.
5	The following statements relate to the ability of CMR to differentiate myocaradial infarction from other diagnoses in patients found to have unobstructed coronary arteries on emergency angiography.

For peer review only

	a. Unlike echocardiography, CMR can provide a definitive ischaemic diagnosis (e.g. myocardial infarction with spontaneous reperfusion or distal embolization) or a non-ischaemic diagnosis (e.g. myocarditis, Takotsubo cardiomyopathy, aortic dissection) in patients with unobstructed coronary arteries on angiography.<br>b. A definitive diagnosis results in a patient treatment plan appropriate for that diagnosis.<br>c. A treatment plan appropriate for the diagnosis is expected to lead to a reduced risk of MACE in the long term.
6	The following statements relate to the ability of CMR to identify patients at high risk of sudden cardiac death after PPCI who would benefit most from an implantable cardiac device.<br>a. CMR identifies PPCI patients who are at high risk of sudden cardiac death better than echocardiography.<br>b. Better identification of PPCI patients at high risk of sudden cardiac death allows optimal patient selection for an implantable cardiac device (ICD or CRT).<br>c. Optimal patient selection for an implantable cardiac device is expected to lead to a reduced risk of MACE in these patients in the long term.
7	The following statements relate to the ability of CMR to identify patients who would not benefit from CRT after PPCI.<br>a. CMR identifies patients who would not benefit from CRT better than echocardiography.<br>b. The ability to identify patients who would not benefit from CRT would reduce CRT use in patients who do not need it.<br>c. Reducing CRT use in patients who do not need it is expected to lead to reduced risk of MACE in these patients in the long term.
8	The following statements relate to the ability of CMR to assess ischemia and viability in patients with multi-vessel disease.<br>a. CMR assesses ischemia and viability of the myocardium better than echocardiography.<br>b. Better assessment of ischemia and viability of the myocardium optimises the revascularisation strategy for patients with multi-vessel disease and avoids additional diagnostic tests.<br>c. The ability to optimise the revascularisation strategy for patients with multi-vessel disease is expected to lead to a reduced risk of MACE in the long term.
9	The following statements relate to the ability of CMR to identify patients with post-infarct LV thrombus.<br>a. CMR identifies post-infarct LV thrombus better than echocardiography.<br>b. Better detection of post-infarct LV thrombus in PPCI patients allows more affected patients to be treated with anticoagulation therapy.<br>c. Treatment with anticoagulation therapy in patients with post-infarct LV thrombus is expected to lead to a reduced risk of MACE in the long term.
10	The following statements relate to the ability of CMR to detect incidental cardiac and non-cardiac findings if offered routinely to patients who undergo an emergency angiogram.<br>a. CMR identifies more incidental cardiac / non-cardiac findings than echocardiography.<br>b. Improved detection of potentially significant incidental findings allows affected patients to be investigated further and / or treated.<br>c. Further investigation and treatment is expected to reduce the risk of MACE / increase overall survival in affected patients in the long term.

Modified survey

For peer review only

For peer review only - http://bmjopen.bmj.com/site/about/guidelines.xhtml

Fifty four cardiologists (including five of the cardiologists who attended the formal consensus meeting) completed the survey. There was consensus that five of the 10 statements (50%) described clinically important changes in management (

Figure). These were statements 3, 5 and 9 (which were in consensus in the first survey) and two additional statements: statement 1, relating to the ability of CMR to identify patients who have a poor prognosis after PPCI; and statement 8, relating to the ability of CMR to assess ischemia and viability in patients with MVD. There was no consensus that any of the other statements described a change in management that was not clinically important.

Figure 2 Median and interquartile (IQ) range for the 10 statements in the modified survey (n=54). Boxes represent the median and IQ range and the whiskers represent the range. Dots represent extreme values. Consensus was based on the distribution of responses to a, b and c. Statements 1, 3, 5, 8 and 9 were considered to be in consensus.

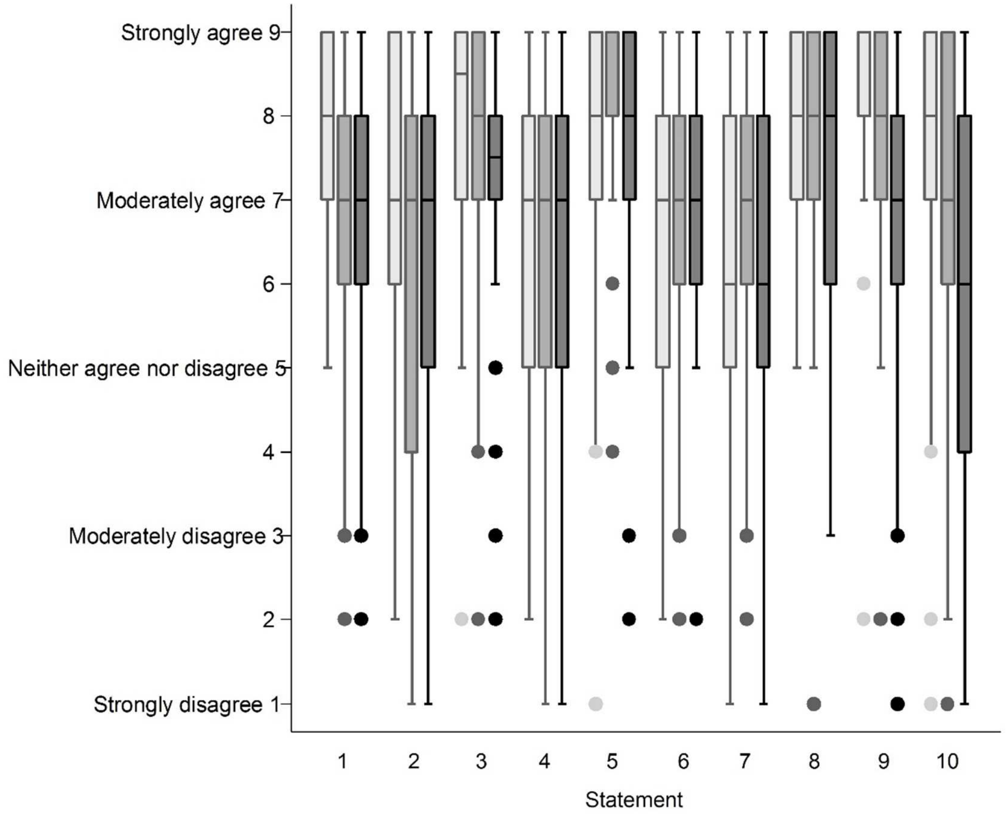

Discussion

For peer review only - http://bmjopen.bmj.com/site/about/guidelines.xhtml

We identified five subgroups of ACS patients who activate the PPCI pathway for whom there was consensus that CMR changes patient management in a clinically important way: i) patients who have an OHCA (about 7% of those who activate the PPCI pathway [4]); ii) patients who have a “normal” (unobstructed) coronary angiogram (about 10% of those who activate the PPCI pathway [5 6]); iii) patients who develop LV thrombus (3% overall and 9% in patients with anterior ST-elevation myocardial infarction, STEMI[7]); iv) patients who have MVD (between 40-65% of those who undergo PPCI [8-10]); and v) patients in whom CMR markers indicate poor prognosis (up to 60% of patients after STEMI [11 12]). There was consensus about the first three subgroups in both rounds of the survey, while consensus was reached about the latter subgroups in the modified survey. These results suggest that CMR benefits a large proportion of patients who activate the PPCI pathway.

Evidence for the roles for CMR in patients who activate the PPCI pathway

Cardiologists who participated in our research agreed that CMR is superior to echocardiography in establishing a diagnosis in patients who survive an OHCA, which has implications for treatment and prognosis. Despite this view, there are few studies that have reported the role of CMR in managing these patients. An unpublished retrospective case series of 54 OHCA survivors showed that CMR diagnosed the cause (ischaemic or non-ischaemic cardiomyopathy) in 40 (74%) [13].

The cardiologists also agreed that CMR can provide a definitive diagnosis (acute MI, acute myocarditis and cardiomyopathy, especially Takotsubo cardiomyopathy) in patients with ACS and unobstructed coronary arteries. Evidence for the role of CMR in patients with ACS and unobstructed coronary arteries also comes from small retrospective case series. These suggest that CMR provides a definitive diagnosis in 80-90% of these patients [14-16]. Without access to CMR, the management of these patients is variable in clinical practice, which may have long-term implications. A recent systematic review showed that the overall all-cause mortality in patients presenting with suspected MI and unobstructed coronary arteries was about 5% at 12 months [17].

For peer review only - http://bmjopen.bmj.com/site/about/guidelines.xhtml

The cardiologists agreed that CMR identifies LV thrombus better than echocardiography, which allows more patients to be identified and treated appropriately. Accurate identification of a LV thrombus is important because it often directs subsequent anticoagulation therapy to prevent embolic events. Evidence for the statement comes from a recent systematic review, published after we conducted the formal consensus study, which showed that late gadolinium enhancement CMR is the most accurate modality for detecting LV thrombus, with 88% sensitivity and 99% specificity (compared with routine echocardiography, which has 24%-33% sensitivity and 94%-95% specificity, and contrast echocardiography, which has 23%-61% sensitivity and 96%-99% specificity) [18].

There was agreement among cardiologists that a CMR-based testing of ischaemia after PPCI after PPCI would likely optimise the revascularisation strategy for these patients, although they acknowledged that there is no evidence to support the view that a CMR-based revascularisation strategy would improve outcomes for patients with MVD (see

For peer review only

For peer review only - http://bmjopen.bmj.com/site/about/guidelines.xhtml

Table 2). Patients with MVD have a two-fold increase in MACE compared with patients with single-vessel disease [19]. There is continuing debate about the benefits of complete vs single lesion revascularisation during the index PPCI admission [20]. Despite recent evidence from randomised controlled trials showing improved clinical outcomes when complete revascularisation is undertaken at PPCI [21], current American and European revascularization guidelines for acute MI recommend revascularisation only of the infarct-related artery at PPCI in patients with MVD [22 23].

There was considerable debate about using CMR to identify patients with poor prognosis after prognosis after PPCI. Whilst there was agreement that CMR parameters of cardiac function (e.g. impaired LV function, large infarct size, microvascular obstruction, etc.) are useful for risk risk stratification after STEMI, there was disagreement about whether this would lead to a management change. Some cardiologists felt that there would be no changes to prescribing for secondary prevention since all patients should receive aggressive secondary prevention according to guidelines (see

For peer review only

For peer review only - http://bmjopen.bmj.com/site/about/guidelines.xhtml

Table 2). It was acknowledged, however, that CMR markers are prognostic for outcome (statement 1a in Error! Reference source not found.); two recent meta analyses of prognostic studies (conducted after this formal consensus study) showed an increased risk of MACE, by 13-15% for every 10% decrease in ejection fraction assessed by CMR [11], and in patients with microvascular obstruction (MVO) assessed by CMR (odds ratio (OR) 2.60, 95% confidence interval (CI) 1.68-4.02 and OR 4.30, 95% CI 2.19-8.43, depending on method of MVO assessment) [24].

Strengths and limitations

A key strength of this study was the use of a formal consensus approach, based on a systematic search of the literature as well as expert opinion. Formal consensus is particularly useful to clarify and standardise practice when relevant and rigorous evidence is lacking. The evidence supporting many of our statements came from small retrospective case series. There were no prospective studies in the literature that reported the impact of CMR on clinical management in our population or whether any changes in management impacted on patient outcomes.

The main features of a formal consensus method are anonymity (statements were rated free from peer-group pressure) and iterative feedback (participants could adjust their initial rating based on the feedback of the group rating). We described the supporting evidence for each statement, identified via the literature review and fed back qualitative (panel members' comments) and quantitative (group median ratings of each statement) information at the face-to-face meeting. We also included a varied group of cardiologists from different specialties in order to encompass diverse perspectives.

The number of panel members in our study was lower than the recommended 8-12 members for a consensus panel [26]. Nevertheless, smaller groups are preferable to larger ones since, although having more group members increases the reliability of group judgement, large groups reduce the ability to elicit potentially important contributions from every member of the panel [26]. To overcome any criticism of the number of panel members

For peer review only - http://bmjopen.bmj.com/site/about/guidelines.xhtml

in our study and to prevent the possibility of introducing bias (given the self-selected nature of the panel), we extended the survey to UK cardiologists who did not participate in the formal consensus process. Apart from one statement (statement 1, relating to the ability of CMR to identify patients with poor prognosis after PPCI), the same statements were in consensus when the survey was completed by cardiologists external to the consensus process. This indicates that our process was robust and our survey statements were clear and unambiguous.

A further limitation of our study was that we did not include other stakeholders (e.g. general practitioners and patient representatives) in the consensus process.[27] Although we had originally planned to do so, we decided that the specialised nature of the statements would not be easily understood by those without cardiology expertise.

Conclusions and future research

We have identified the main ways in which cardiologists believe CMR changes clinical management in five subgroups of patients who activate the PPCI pathway. These clinically important changes in management can now be used to design a composite primary outcome for an evaluation of the effectiveness and cost-effectiveness of CMR. It will allow evidence to be obtained more quickly to inform decisions about implementing CMR than would be possible for an evaluation based on MACE.

Authors' contributions

MP: determined the structure of the consensus process, conducted the literature review, formulated statements based on the literature review, was a member of the working group and wrote the manuscript.

RB: contributed to the consensus process, was a member of the working group, co-wrote the manuscript and approved the final manuscript.

For peer review only - http://bmjopen.bmj.com/site/about/guidelines.xhtml

For peer review only

CBD: conceived the overall feasibility study, contributed to the consensus process, was a member of the working group, revised the manuscript with respect to intellectual content and approved the final manuscript.

JPG: contributed to the consensus process, was a member of the working group, revised the manuscript with respect to intellectual content and approved the final manuscript.

SD: contributed to the consensus process, was a member of the working group, revised the manuscript with respect to intellectual content and approved the final manuscript.

RA: contributed to the consensus process, was a member of the working group, revised the manuscript with respect to intellectual content and approved the final manuscript.

JH: reviewed the statistical analysis, was a member of the working group, revised the manuscript with respect to intellectual content and approved the final manuscript

EM: contributed to the formulation of the consensus statements, revised the manuscript with respect to intellectual content and approved the final manuscript

CR: reviewed the analysis, was a member of the working group and approved the final manuscript

BR: chief investigator who conceived the overall feasibility study, including the consensus element, was a member of the working group, revised the manuscript with respect to intellectual content and approved the final manuscript.

Funding

This study is part of a project funded by the National Institute of Health Research (NIHR) Health Services & Delivery Research (HS&DR 11/2003/58). The British Heart Foundation and NIHR Bristol Biomedical Research Unit for Cardiovascular Disease funded some staff time (CAR, CBD, JH, EM, BCR). The views and opinions expressed are those of the authors and do not necessarily reflect those of the HS&DR programme, NIHR, the UK NHS or the Department of Health.

Competing interests

For peer review only - http://bmjopen.bmj.com/site/about/guidelines.xhtml

For peer review only

JPG has received a research grant from Philips Healthcare. The remaining authors declare that they have no competing interests.

Data sharing statement

The literature search based on the published search strategy, details of development of the consensus statements and the survey results can be obtained by contacting the corresponding author (maria.pufulete@bristol.ac.uk).

For peer review only

Acknowledgements

This project was funded by the National Institute for Health Research HS & DR Programme (project number 11/2003/58). The views and opinions expressed therein are those of the authors and do not necessarily reflect those of the HS & DR Programme, NIHR, NHS or the Department of Health.

References

1. Antony R, Daghem M, McCann GP, et al. Cardiovascular magnetic resonance activity in the United Kingdom: a survey on behalf of the British Society of Cardiovascular Magnetic Resonance. J Cardiovasc Magn Reson 2011;**13**:57.
2. http://www.nets.nihr.ac.uk/projects/hsdr/11200358.
3. Rycroft-Malone J. Formal consensus: the development of a national clinical guideline. Qual Health Care 2001;**10**(4):238-44.
4. Choudry FA, Weerackody RP, Timmis AD, et al. Importance of primary percutaneous coronary intervention for reducing mortality in ST-elevation myocardial infarction complicated by out of hospital cardiac arrest. European heart journal Acute cardiovascular care 2015;**4**(4):378-85.
5. Larsen AI, Galbraith PD, Ghali WA, et al. Characteristics and outcomes of patients with acute myocardial infarction and angiographically normal coronary arteries. Am J Cardiol 2005;**95**(2):261-3.
6. Meryon I, Patel N, Millane T, et al. Normal coronary angiography and primary percutaneous coronary intervention for ST elevation myocardial infarction: a literature review and audit findings. Int J Clin Pract 2010;**64**(9):1245-51.
7. Robinson AA, Jain A, Gentry M, et al. Left ventricular thrombi after STEMI in the primary PCI era: A systematic review and meta-analysis. Int J Cardiol 2016;**221**:554-59.
8. Corpus RA, House JA, Marso SP, et al. Multivessel percutaneous coronary intervention in patients with multivessel disease and acute myocardial infarction. Am Heart J 2004;**148**(3):493-500.
9. Goldstein JA, Demetriou D, Grines CL, et al. Multiple complex coronary plaques in patients with acute myocardial infarction. N Engl J Med 2000;**343**(13):915-22.

10. Toma M, Buller CE, Westerhout CM, et al. Non-culprit coronary artery percutaneous coronary intervention during acute ST-segment elevation myocardial infarction: insights from the APEX-AMI trial. Eur Heart J 2010;**31**(14):1701-7.
11. El Aidi H, Adams A, Moons KG, et al. Cardiac magnetic resonance imaging findings and the risk of cardiovascular events in patients with recent myocardial infarction or suspected or known coronary artery disease: a systematic review of prognostic studies. J Am Coll Cardiol 2014;**63**(11):1031-45.
12. van Kranenburg M, Magro M, Thiele H, et al. Prognostic value of microvascular obstruction and infarct size, as measured by CMR in STEMI patients. JACC Cardiovasc Imaging 2014;**7**(9):930-9.
13. Dastidar AG, Ahmed A, McAlindon E, et al. Incremental Value of Cardiovascular Magnetic Resonance Imaging in Patients Surviving non Traumatic out of Hospital Cardiac Arrest: A Tertiary UK Centre Experience. Heart (British Cardiac Society) 2014;**100**:A79-A80.
14. Assomull RG, Lyne JC, Keenan N, et al. The role of cardiovascular magnetic resonance in patients presenting with chest pain, raised troponin, and unobstructed coronary arteries. Eur Heart J 2007;**28**(10):1242-9.
15. Leurent G, Langella B, Fougerou C, et al. Diagnostic contributions of cardiac magnetic resonance imaging in patients presenting with elevated troponin, acute chest pain syndrome and unobstructed coronary arteries. Arch Cardiovasc Dis 2011;**104**(3):161-70.
16. Monney PA, Sekhri N, Burchell T, et al. Acute myocarditis presenting as acute coronary syndrome: role of early cardiac magnetic resonance in its diagnosis. Heart (British Cardiac Society) 2011;**97**(16):1312-8.
17. Pasupathy S, Air T, Dreyer RP, et al. Systematic review of patients presenting with suspected myocardial infarction and nonobstructive coronary arteries. Circulation 2015;**131**(10):861-70.
18. Roifman I, Connelly KA, Wright GA, et al. Echocardiography vs. Cardiac Magnetic Resonance Imaging for the Diagnosis of Left Ventricular Thrombus: A Systematic Review. The Canadian journal of cardiology 2015;**31**(6):785-91.
19. Sorajja P, Gersh BJ, Cox DA, et al. Impact of multivessel disease on reperfusion success and clinical outcomes in patients undergoing primary percutaneous coronary intervention for acute myocardial infarction. Eur Heart J 2007;**28**(14):1709-16.
20. Ruggieri A, Piraino D, Dendramis G, et al. STEMI patients and nonculprit lesions: To treat or not to treat? and when? A review of most recent literature. Catheter Cardiovasc Interv 2015.
21. El-Hayek GE, Gershlick AH, Hong MK, et al. Meta-Analysis of Randomized Controlled Trials Comparing Multivessel Versus Culprit-Only Revascularization for Patients With ST-Segment Elevation Myocardial Infarction and Multivessel Disease Undergoing Primary Percutaneous Coronary Intervention. Am J Cardiol 2015;**115**(11):1481-6.
22. O'Gara PT, Kushner FG, Ascheim DD, et al. 2013 ACCF/AHA guideline for the management of ST-elevation myocardial infarction: a report of the American College of Cardiology Foundation/American Heart Association Task Force on Practice Guidelines. J Am Coll Cardiol 2013;**61**(4):e78-140.
23. Steg PG, James SK, Atar D, et al. ESC Guidelines for the management of acute myocardial infarction in patients presenting with ST-segment elevation. Eur Heart J 2012;**33**(20):2569-619.
24. Hamirani YS, Wong A, Kramer CM, et al. Effect of microvascular obstruction and intramyocardial hemorrhage by CMR on LV remodeling and outcomes after myocardial infarction: a systematic review and meta-analysis. JACC Cardiovasc Imaging 2014;**7**(9):940-52.
25. https://www.nice.org.uk/guidance/cg3/resources/guidance-preoperative-tests-pdf.
26. Murphy MK, Black NA, Lamping DL, et al. Consensus development methods, and their use in clinical guideline development. Health technology assessment (Winchester, England) 1998;**2**(3):i-iv, 1-88.

For peer review only - http://bmjopen.bmj.com/site/about/guidelines.xhtml

27. Hutchings A, Raine R. A systematic review of factors affecting the judgments produced by formal consensus development methods in health care. Journal of health services research & policy 2006;**11**(3):172-9.

For peer review only

For peer review only - http://bmjopen.bmj.com/site/about/guidelines.xhtml

Benefit to patients and the NHS of cardiac magnetic resonance imaging (CMR) after Primary percutaneous coronary Intervention (PPCI) Pathway Activation

Thank you for agreeing to complete this survey. We expect the survey to take about 20 minutes of your time. You are asked to read and rate 12 statements relating to the potential impact of cardiac magnetic resonance imaging (CMR) in changing the management of patients in a clinically important way. The patient population under consideration includes all patients who activate the primary percutaneous coronary intervention (PPCI) pathway. Please rate your agreement with each statement on the scale provided. Each statement has a supporting paragraph that provides background information and links to full text references. We believe we have used the best available evidence but we are aware that the evidence is of variable quality.

In order to progress through the survey, please use the following navigation buttons:

Click the Next button to go to the next page.

Click the Previous button to return to the previous page.

Click the Number buttons in the text to access the full references.

Click the Submit button to submit the survey at the end.

Statement 1. *Compared with echocardiography, CMR after PPCI allows patients with CMR markers that indicate **poor** prognosis (e.g. impaired left ventricular function, large infarct size, microvascular obstruction) to be followed up more appropriately and undergo more aggressive medical therapy for secondary prevention.*

Accurate assessment of infarct characteristics is important for risk stratification after PPCI. CMR can quantify in a single scan all cardiac markers relevant to PPCI outcomes, with high reproducibility and accuracy. CMR has high spatial and temporal resolution and is superior to echocardiography for measuring left ventricular volumes and ejection fraction. **[1]** Additionally, CMR markers such as infarct size, microvascular obstruction and myocardial salvage have been shown to have long term prognostic value. **[2-4]** Late gadolinium enhancement CMR has added prognostic value over echocardiography. **[5]**

Statement 2. *Compared with echocardiography, CMR after PPCI allows patients with CMR markers that indicate **good** prognosis (e.g. normal left ventricular function, high myocardial salvage, no microvascular obstruction, no residual ischemia) to be discharged earlier and followed up less frequently.*

CMR measures several markers (infarct size, myocardial salvage index, microvascular obstruction, myocardial oedema and haemorrhage) that cannot be measured by echocardiography. These markers can be used to predict cardiac remodelling and prognosis after myocardial infarction, **[2, 4]** determine the optimal therapeutic pathway for each patient and prevent over- and under-treatment.

Statement 3. *Compared with echocardiography, CMR better identifies the cause of out of hospital cardiac arrest [e.g. large myocardial infarction, arrhythmogenic right ventricular cardiomyopathy (ARVC), aberrant coronary arteries, hypertrophic cardiomyopathy (HCM)] to optimise further treatment for the patient (e.g. defibrillator for primary arrhythmia or percutaneous coronary intervention) or family members.*

Out-of-hospital cardiac arrest affects about 60,000 people in the UK each year. Currently, hospital survival is about 32%. Early identification of the cause is essential to improve survival. Causes of out-of-hospital cardiac arrest include myocardial infarction (40%-90%) and inherited cardiomyopathies such as ARVC and HCM. Unlike echocardiography, CMR allows in vivo tissue characterisation, which differentiates scarring resulting from myocardial infarction from other causes of focal fibrosis (e.g. observed in non-ischaemic cardiomyopathies such as ARVC and HCM). **[6]** Late gadolinium enhancement on CMR can predict serious cardiac complications in patients with HCM (all-cause death, cardiac death and death from heart failure), **[7]** and identifies patients that may need more aggressive medical and device therapy (e.g. renin-aldosterone system inhibition for prevention of heart failure or implantable cardioverter defibrillator placement for primary prevention of sudden cardiac death).

Statement 4. *CMR after PPCI identifies patients at high risk of having ventricular septal defect (VSD) or impending cardiac rupture, who may require ventricular patch or other urgent cardiac surgery, and guides the optimal management of these patients.*

VSD and left ventricular free wall rupture are rare complications of myocardial infarction, occurring in less than 1% of PPCI patients, usually within one week of the infarct. Mortality ranges between 50% and 80%. Diagnosis can be difficult to make and usually requires multi-modality imaging (echocardiography, ventriculography, computed tomography, CMR) before surgical repair. Because of its high spatial resolution, CMR can be used to clarify the detailed structure of these lesions. CMR accurately identifies the location, size and tissue margins of VSD and is useful for detecting apical defects, which are not easily identified by echocardiography. CMR measurements may also be used to determine the size of ventricular patch required to close the VSD, which avoids the inflation of a sizing balloon in friable infarcted tissue. **[8]** These features, together with other CMR markers of damage (e.g. infarct size, microvascular obstruction, left ventricular dysfunction) are useful for guiding optimal management of patients with post-infarct VSD. Similarly, CMR can differentiate between the different types of impending free wall rupture (acute, sub-acute and chronic), which may be helpful in planning surgical management. **[9]**

Statement 5. *In patients with a "normal" (unobstructed) coronary angiogram, CMR can differentiate patients who have had a myocardial infarction with spontaneous reperfusion or distal embolization from patients with a non-ischaemic diagnosis (e.g. myocarditis, Takotsubo cardiomyopathy, aortic dissection), resulting in a patient treatment plan appropriate for the definitive diagnosis.*

The incidence of unobstructed coronary angiogram in patients who activate the PPCI pathway is between 5% and 12%. In these patients, the lack of an accurate diagnosis may result in inappropriate or unnecessary treatment and/or follow-up and poorer prognosis. **[10]** CMR facilitates differential diagnosis in the context of an unobstructed coronary angiogram, providing a definitive diagnosis (e.g. myocardial infarction, myocarditis, Takotsubo cardiomyopathy) in 65-90% of these patients. **[11, 12]** In patients with myocarditis, late gadolinium enhancement (LGE) on CMR may predict long term adverse outcomes. **[13]** In the context of myocardial infarction without an angiographic lesion, CMR can locate the

For peer review only

culprit infarct-related artery in patients with spontaneous reperfusion or with distal embolization.

Statement 6. *Compared with echocardiography, CMR after PPCI better identifies patients at high risk of sudden cardiac death who **would** benefit most from an implantable cardiac device [e.g.* implantable cardioverter defibrillator (*ICD), cardiac resynchronisation therapy, (CRT)].*

About 6% of heart attack patients subsequently die suddenly from a presumed cardiac cause. Current guidelines recommend the use of ICD to prevent sudden cardiac death in patients who have a low left ventricular ejection fraction (EF) after a heart attack. EF is used in clinical practice to make decisions about ICD implantation, but it has a low predictive value and many patients with an ICD will never benefit from it. **[14]** EF is most commonly measured by echocardiography, but CMR is now considered the gold standard for EF measurement because it is more reproducible than echocardiography. **[1]** CMR has been shown to be better than echocardiography for selecting patients for ICD implantation when strict EF thresholds are used to guide implantation. **[15]** The extent of myocardial scar characterised by late gadolinium enhancement (LGE) CMR may also be used to predict whether ICD implantation is appropriate in this patient group. **[16]** Furthermore, LGE CMR can guide placement of the left ventricular lead away from scarred myocardium, which results in a better clinical outcome after CRT. **[17]**

Statement 7. *Compared with echocardiography, CMR after PPCI better identifies patients who **would not** benefit from cardiac resynchronisation therapy (CRT).*

CRT (or biventricular pacing) uses a specialised pacemaker to resynchronise the beating of the two ventricles by pacing both simultaneously, which improves the contraction of the left ventricle and the overall efficiency of the heart. It is used in patients with systolic ventricular dysfunction and heart failure. However, about 30% of patients who meet the inclusion criteria for CRT do not respond to it. **[18]** CMR assessment of mechanical dyssynchrony and myocardial scar provides additional value over echocardiography for identifying responsive patients. **[19, 20]** Myocardial scar is an important feature of non-response to CRT **[21, 22]** and late gadolinium enhancement (LGE) CMR accurately differentiates between transmural, mid-myocardial, epicardial, and subendocardial scar.

Statement 8. *Compared with stress echocardiography* or *single photon emission computed tomography (SPECT), CMR after PPCI in patients with multi-vessel disease better assesses ischaemia and viability of the myocardium to optimise the revascularisation strategy for the patient and avoid additional diagnostic tests.*

Between 40% and 65% of the patients who activate the PPCI pathway have multi-vessel disease. Adequate assessment of residual ischemia in non-culprit arteries post PPCI is important for effective management because this patient group has an adverse prognosis. Various techniques are currently used to assess the need for additional revascularisation, including stress echocardiography, SPECT and stress-perfusion CMR. Stress CMR has excellent prognostic value **[23]** and better diagnostic accuracy than SPECT **[24, 25]** or stress echocardiography **[26]** for detecting angiographically significant coronary artery disease.

Statement 9. *Compared with echocardiography, CMR after PPCI better identifies patients with left ventricular (LV) thrombus for treatment with anticoagulation therapy.*

For peer review only

LV thrombus is a serious complication of acute myocardial infarction. It increases risk of thromboembolic events, particularly stroke. LV thrombus develops in up to 10% of patients with anterior wall infarctions after PPCI. Although echocardiography is most commonly used to detect LV thrombus and to assess its shape and size, between 10-46% of echocardiograms are inconclusive. **[27]** Late gadolinium enhancement (LGE) CMR is considered the gold standard for detecting LV thrombus, because it detects thrombus based on tissue characteristics rather than anatomic appearance. Fewer thrombi are detected by contrast-echocardiography than by LGE CMR. **[28]** CMR has a higher sensitivity (88%) than contrast (61%) and noncontrast (<33%) echocardiography. **[28, 29]**

Statement 10. *Compared with other imaging modalities, in patients with multi-vessel disease, CMR after PPCI better identifies the artery that caused the myocardial infarction and guides the subsequent treatment plan in relation to additional revascularisation (percutaneous coronary intervention, or coronary artery bypass graft surgery).*

In patients with multi vessel disease presenting with acute MI, the culprit artery is not always easy to identify as more than one artery could be responsible. CMR has higher spatial resolution than single photon emission computed tomography (SPECT), allowing easy identification of the infarct-related artery and non-culprit territory stenosis in PPCI patients, **[30]** and hence appropriate risk-stratification of patients with multi-vessel disease for planning their future treatment. **[31]**

Statement 11. *Compared with echocardiography, CMR after PPCI better identifies incidental cardiac findings that may need further investigation and treatment.*

Compared with echocardiography, CMR provides versatile imaging planes, superior tissue contrast and advanced tissue characterisation, allowing a comprehensive assessment of cardiac anatomy, function and flow, and imaging of the great vessels (including venous return), the pericardium and suspected cardiac tumours. **[32]** As such, CMR can identify congenital coronary anomalies, cardiac masses, coronary artery aneurysms, valvular heart disease, thoracic aortic disease, etc. CMR can adequately differentiate benign from malignant tumours in the heart. **[33]**

Statement 12. *Compared with echocardiography, CMR after PPCI identifies significant incidental non-cardiac findings (e.g. oesophageal and lung tumours, pulmonary embolus, aortic aneurysm) that may need further investigation and treatment.*

Unlike echocardiography, which provides limited imaging of mediastinal and extra-cardiac structures, CMR images a substantial part of the thorax and abdomen in the field of view, which may potentially contain non-cardiac abnormalities. The prevalence of non-cardiac findings on CMR is up to 80% (with up to 30% of these representing potentially significant findings), depending on the characteristics of the population examined. **[34-36]**

References

1. Dorosz JL, Lezotte DC, Weitzenkamp DA, Allen LA, Salcedo EE: **Performance of 3-dimensional echocardiography in measuring left ventricular volumes and ejection fraction: a systematic review and meta-analysis**. *J Am Coll Cardiol* 2012, **59**(20):1799-1808.
2. Eitel I, Desch S, de WS, Fuernau G, Gutberlet M, Schuler G, Thiele H: **Long-term prognostic value of myocardial salvage assessed by cardiovascular magnetic resonance in acute reperfused myocardial infarction**. *Heart* 2011, **97**(24):2038-2045.
3. Eitel I, Desch S, Fuernau G, Hildebrand L, Gutberlet M, Schuler G, Thiele H: **Prognostic significance and determinants of myocardial salvage assessed by cardiovascular magnetic**

resonance in acute reperfused myocardial infarction. *J Am Coll Cardiol* 2010, **55**(22):2470-2479.

4. Klug G, Mayr A, Schenk S, Esterhammer R, Schocke M, Nocker M, Jaschke W, Pachinger O, Metzler B: **Prognostic value at 5 years of microvascular obstruction after acute myocardial infarction assessed by cardiovascular magnetic resonance**. *Journal of Cardiovascular Magnetic Resonance* 2012, **14**:46.
5. Wong TC, Piehler K, Puntil KS, Moguillansky D, Meier CG, Lacomis JM, Kellman P, Cook SC, Schwartzman DS, Simon MA *et al*: **Effectiveness of late gadolinium enhancement to improve outcomes prediction in patients referred for cardiovascular magnetic resonance after echocardiography**. *Journal of cardiovascular magnetic resonance : official journal of the Society for Cardiovascular Magnetic Resonance* 2013, **15**:6.
6. Parsai C, O'Hanlon R, Prasad SK, Mohiaddin RH: **Diagnostic and prognostic value of cardiovascular magnetic resonance in non-ischaemic cardiomyopathies**. *Journal of cardiovascular magnetic resonance : official journal of the Society for Cardiovascular Magnetic Resonance* 2012, **14**:54.
7. Green JJ, Berger JS, Kramer CM, Salerno M: **Prognostic value of late gadolinium enhancement in clinical outcomes for hypertrophic cardiomyopathy**. *JACC Cardiovascular imaging* 2012, **5**(4):370-377.
8. Artis NJ, Thomson J, Plein S, Greenwood JP: **Percutaneous closure of postinfarction ventricular septal defect: cardiac magnetic resonance-guided case selection and postprocedure evaluation**. *The Canadian journal of cardiology* 2011, **27**(6):869 e863-865.
9. Galvin S, Chen V, Bunton R, Doyle T: **Sutureless pericardial patch augmentation for impending left ventricular free wall rupture**. *Interactive cardiovascular and thoracic surgery* 2010, **10**(2):341-343.
10. Dokainish H, Pillai M, Murphy SA, DiBattiste PM, Schweiger MJ, Lotfi A, Morrow DA, Cannon CP, Braunwald E, Lakkis N: **Prognostic implications of elevated troponin in patients with suspected acute coronary syndrome but no critical epicardial coronary disease: a TACTICS-TIMI-18 substudy**. *J Am Coll Cardiol* 2005, **45**(1):19-24.
11. Assomull RG, Lyne JC, Keenan N, Gulati A, Bunce NH, Davies SW, Pennell DJ, Prasad SK: **The role of cardiovascular magnetic resonance in patients presenting with chest pain, raised troponin, and unobstructed coronary arteries**. *Eur Heart J* 2007, **28**(10):1242-1249.
12. Leurent G, Langella B, Fougerou C, Lentz PA, Larralde A, Bedossa M, Boulmier D, Le Breton H: **Diagnostic contributions of cardiac magnetic resonance imaging in patients presenting with elevated troponin, acute chest pain syndrome and unobstructed coronary arteries**. *Arch Cardiovasc Dis* 2011, **104**(3):161-170.
13. Grun S, Schumm J, Greulich S, Wagner A, Schneider S, Bruder O, Kispert EM, Hill S, Ong P, Klingel K *et al*: **Long-term follow-up of biopsy-proven viral myocarditis: predictors of mortality and incomplete recovery**. *J Am Coll Cardiol* 2012, **59**(18):1604-1615.
14. Moss AJ, Greenberg H, Case RB, Zareba W, Hall WJ, Brown MW, Daubert JP, McNitt S, Andrews ML, Elkin AD *et al*: **Long-term clinical course of patients after termination of ventricular tachyarrhythmia by an implanted defibrillator**. *Circulation* 2004, **110**(25):3760-3765.
15. Joshi SB, Connelly KA, Jimenez-Juan L, Hansen M, Kirpalani A, Dorian P, Mangat I, Al-Hesayen A, Crean AM, Wright GA *et al*: **Potential clinical impact of cardiovascular magnetic resonance assessment of ejection fraction on eligibility for cardioverter defibrillator implantation**. *Journal of cardiovascular magnetic resonance : official journal of the Society for Cardiovascular Magnetic Resonance* 2012, **14**:69.
16. Alexandre J, Saloux E, Dugue AE, Lebon A, Lemaitre A, Roule V, Labombarda F, Provost N, Gomes S, Scanu P *et al*: **Scar extent evaluated by late gadolinium enhancement CMR: a powerful predictor of long term appropriate ICD therapy in patients with coronary artery**

For peer review only

disease. *Journal of cardiovascular magnetic resonance : official journal of the Society for Cardiovascular Magnetic Resonance* 2013, **15**:12.

17. Leyva F, Foley PW, Chalil S, Ratib K, Smith RE, Prinzen F, Auricchio A: **Cardiac resynchronization therapy guided by late gadolinium-enhancement cardiovascular magnetic resonance**. *Journal of cardiovascular magnetic resonance : official journal of the Society for Cardiovascular Magnetic Resonance* 2011, **13**:29.
18. Leclercq C, Kass DA: **Retiming the failing heart: principles and current clinical status of cardiac resynchronization**. *J Am Coll Cardiol* 2002, **39**(2):194-201.
19. Bilchick KC, Dimaano V, Wu KC, Helm RH, Weiss RG, Lima JA, Berger RD, Tomaselli GF, Bluemke DA, Halperin HR *et al*: **Cardiac magnetic resonance assessment of dyssynchrony and myocardial scar predicts function class improvement following cardiac resynchronization therapy**. *JACC Cardiovascular imaging* 2008, **1**(5):561-568.
20. Ellims AH, Pfluger H, Elsik M, Butler MJ, Hare JL, Taylor AJ: **Utility of cardiac magnetic resonance imaging, echocardiography and electrocardiography for the prediction of clinical response and long-term survival following cardiac resynchronisation therapy**. *Int J Cardiovasc Imaging* 2013, **29**(6):1303-1311.
21. Adelstein EC, Saba S: **Scar burden by myocardial perfusion imaging predicts echocardiographic response to cardiac resynchronization therapy in ischemic cardiomyopathy**. *Am Heart J* 2007, **153**(1):105-112.
22. White JA, Yee R, Yuan X, Krahn A, Skanes A, Parker M, Klein G, Drangova M: **Delayed enhancement magnetic resonance imaging predicts response to cardiac resynchronization therapy in patients with intraventricular dyssynchrony**. *J Am Coll Cardiol* 2006, **48**(10):1953-1960.
23. Lipinski MJ, McVey CM, Berger JS, Kramer CM, Salerno M: **Prognostic value of stress cardiac magnetic resonance imaging in patients with known or suspected coronary artery disease: a systematic review and meta-analysis**. *J Am Coll Cardiol* 2013, **62**(9):826-838.
24. Greenwood JP, Maredia N, Younger JF, Brown JM, Nixon J, Everett CC, Bijsterveld P, Ridgway JP, Radjenovic A, Dickinson CJ *et al*: **Cardiovascular magnetic resonance and single-photon emission computed tomography for diagnosis of coronary heart disease (CE-MARC): a prospective trial**. *Lancet* 2012, **379**(9814):453-460.
25. Schwitter J, Wacker CM, Wilke N, Al-Saadi N, Sauer E, Huettle K, Schonberg SO, Debl K, Strohm O, Ahlstrom H *et al*: **Superior diagnostic performance of perfusion-cardiovascular magnetic resonance versus SPECT to detect coronary artery disease: The secondary endpoints of the multicenter multivendor MR-IMPACT II (Magnetic Resonance Imaging for Myocardial Perfusion Assessment in Coronary Artery Disease Trial)**. *Journal of cardiovascular magnetic resonance : official journal of the Society for Cardiovascular Magnetic Resonance* 2012, **14**:61.
26. Nagel E, Lehmkuhl HB, Bocksch W, Klein C, Vogel U, Frantz E, Ellmer A, Dreysse S, Fleck E: **Noninvasive diagnosis of ischemia-induced wall motion abnormalities with the use of high-dose dobutamine stress MRI: comparison with dobutamine stress echocardiography**. *Circulation* 1999, **99**(6):763-770.
27. Delewi R, Zijlstra F, Piek JJ: **Left ventricular thrombus formation after acute myocardial infarction**. *Heart* 2012, **98**(23):1743-1749.
28. Weinsaft JW, Kim RJ, Ross M, Krauser D, Manoushagian S, LaBounty TM, Cham MD, Min JK, Healy K, Wang Y *et al*: **Contrast-enhanced anatomic imaging as compared to contrast-enhanced tissue characterization for detection of left ventricular thrombus**. *JACC Cardiovascular imaging* 2009, **2**(8):969-979.
29. Srichai MB, Junor C, Rodriguez LL, Stillman AE, Grimm RA, Lieber ML, Weaver JA, Smedira NG, White RD: **Clinical, imaging, and pathological characteristics of left ventricular thrombus: a comparison of contrast-enhanced magnetic resonance imaging, transthoracic**

For peer review only

echocardiography, and transesophageal echocardiography with surgical or pathological validation. *Am Heart J* 2006, **152**(1):75-84.

30. Wong DT, Leung MC, Das R, Liew GY, Williams K, Dundon BK, Molaee P, Teo KS, Meredith IT, Worthley MI *et al*: **Diagnostic accuracy of adenosine stress cardiovascular magnetic resonance following acute ST-segment elevation myocardial infarction post primary angioplasty**. *Journal of Cardiovascular Magnetic Resonance* 2011, **13**:62.
31. Doesch C, Seeger A, Doering J, Herdeg C, Burgstahler C, Claussen CD, Gawaz M, Miller S, May AE: **Risk stratification by adenosine stress cardiac magnetic resonance in patients with coronary artery stenoses of intermediate angiographic severity**. *Jacc: Cardiovascular Imaging* 2009, **2**(4):424-433.
32. American College of Cardiology Foundation Task Force on Expert Consensus D, Hundley WG, Bluemke DA, Finn JP, Flamm SD, Fogel MA, Friedrich MG, Ho VB, Jerosch-Herold M, Kramer CM *et al*: **ACCF/ACR/AHA/NASCI/SCMR 2010 expert consensus document on cardiovascular magnetic resonance: a report of the American College of Cardiology Foundation Task Force on Expert Consensus Documents**. *Circulation* 2010, **121**(22):2462-2508.
33. Hoffmann U, Globits S, Schima W, Loewe C, Puig S, Oberhuber G, Frank H: **Usefulness of magnetic resonance imaging of cardiac and paracardiac masses**. *The American journal of cardiology* 2003, **92**(7):890-895.
34. Chan PG, Smith MP, Hauser TH, Yeon SB, Appelbaum E, Rofsky NM, Manning WJ: **Noncardiac pathology on clinical cardiac magnetic resonance imaging**. *JACC Cardiovascular imaging* 2009, **2**(8):980-986.
35. McKenna DA, Laxpati M, Colletti PM: **The prevalence of incidental findings at cardiac MRI**. *The open cardiovascular medicine journal* 2008, **2**:20-25.
36. Wyttenbach R, Medioni N, Santini P, Vock P, Szucs-Farkas Z: **Extracardiac findings detected by cardiac magnetic resonance imaging**. *Eur Radiol* 2012, **22**(6):1295-1302.

For peer review only

Accurate assessment of infarct characteristics is important for risk stratification after PPCI. CMR can quantify in a single scan all cardiac markers relevant to PPCI outcomes, with high reproducibility and accuracy. CMR has high spatial and temporal resolution and is superior to echocardiography for measuring left ventricular volumes and ejection fraction. **[1]** Additionally, CMR markers that cannot be measured by echocardiography, such as infarct size, microvascular obstruction and myocardial salvage have been shown to have long term prognostic value. **[2-4]** Late gadolinium enhancement CMR has added prognostic value over echocardiography. **[5]**

1. <u>The following statements relate to the ability of CMR to identify patients who have a **poor** prognosis after PPCI.</u>

   a. CMR markers (e.g. impaired left ventricular function, large infarct size, microvascular obstruction) better identify patients with a poor prognosis after PPCI than markers based on echocardiography.

Strongly disagree	Moderately disagree	Neither agree nor disagree	Moderately agree	Strongly agree

   b. Better identification of patients with a poor prognosis after PPCI allows these patients to be followed up more appropriately and treated more aggressively

Strongly disagree	Moderately disagree	Neither agree nor disagree	Moderately agree	Strongly agree

   c. More appropriate follow up and more aggressive treatment in these patients is expected to lead to a reduced risk of MACE in the long term.

Strongly disagree	Moderately disagree	Neither agree nor disagree	Moderately agree	Strongly agree

   d. More appropriate follow up and more aggressive treatment in these patients is expected to lead to less NHS resource use in the long term.

Strongly disagree	Moderately disagree	Neither agree nor disagree	Moderately agree	Strongly agree

   e. The anticipated benefit (reduced risk of MACE and/or less NHS resource use) is likely to be sufficiently large to make CMR cost effective among the patients in whom it would be indicated.

Strongly disagree	Moderately disagree	Neither agree nor disagree	Moderately agree	Strongly agree

2. <u>The following statements relate to the ability of CMR to identify patients who have a **good** prognosis after PPCI.</u>

   a. CMR markers (e.g. normal left ventricular function, high myocardial salvage, no microvascular obstruction, no residual ischemia) better identify patients with a good prognosis after PPCI than markers based on echocardiography.

Strongly disagree	Moderately disagree	Neither agree nor disagree	Moderately agree	Strongly agree

   b. Better identification of patients with a good prognosis after PPCI allows these patients to be followed up less frequently.

Strongly disagree	Moderately disagree	Neither agree nor disagree	Moderately agree	Strongly agree

   c. Less frequent follow up in these patients is expected to lead to less NHS resource use in the long term.

Strongly disagree	Moderately disagree	Neither agree nor disagree	Moderately agree	Strongly agree

   d. The anticipated benefit (less NHS resource use) is likely to be sufficiently large to make CMR cost effective among the patients in whom it would be indicated.

Strongly disagree	Moderately disagree	Neither agree nor disagree	Moderately agree	Strongly agree

For peer review only

Out-of-hospital cardiac arrest (OHCA) affects about 60,000 people in the UK each year. Currently, hospital survival among patients alive on arrival is about 32%. Early identification of the cause of OHCA is essential to improve survival. Causes of OHCA include myocardial infarction (40%-90%) and inherited cardiomyopathies such as arrhythmogenic right ventricular cardiomyopathy (ARVC) and hypertrophic cardiomyopathy (HCM). Unlike echocardiography, CMR allows *in vivo* tissue characterisation, which differentiates scarring due to myocardial infarction from other causes of focal fibrosis (e.g. observed in non-ischaemic cardiomyopathies such as ARVC and HCM).**[6]** Late gadolinium enhancement on CMR can predict serious cardiac complications in patients with HCM (all-cause death, cardiac death and death from heart failure),**[7]** and identifies patients that may need more aggressive medical and device therapy (e.g. renin-aldosterone system inhibition for prevention of heart failure or implantable cardioverter defibrillator placement for primary prevention of sudden cardiac death.

3. <u>The following statements relate to the ability of CMR to identify the causes of OHCA in patients who undergo an emergency angiogram.</u>

   a. CMR better identifies the cause of OHCA (e.g. large myocardial infarction, ARVC, aberrant coronary arteries, HCM) than echocardiography.

Strongly disagree	Moderately disagree	Neither agree nor disagree	Moderately agree	Strongly agree

   b. Better identification of the cause of out-of-hospital cardiac arrest allows treatment to be optimised for these patients (e.g. defibrillator for primary arrhythmia or percutaneous coronary intervention) or their family members (e.g. genetic screening and counselling, primary prevention).

Strongly disagree	Moderately disagree	Neither agree nor disagree	Moderately agree	Strongly agree

   c. The ability to optimise treatment for these patients or family members is expected to lead to a reduced risk of MACE in the long term.

Strongly disagree	Moderately disagree	Neither agree nor disagree	Moderately agree	Strongly agree

   d. The ability to optimise treatment for these patients or family members is expected to lead to less NHS resource use in the long term.

Strongly disagree	Moderately disagree	Neither agree nor disagree	Moderately agree	Strongly agree

   e. The anticipated benefit (reduced risk of MACE and/or less NHS resource use) is likely to be sufficiently large to make CMR cost effective among the patients in whom it would be indicated.

Strongly disagree	Moderately disagree	Neither agree nor disagree	Moderately agree	Strongly agree

Ventricular septal defect (VSD) and left ventricular free wall rupture are rare complications of myocardial infarction, occurring in less than 1% of PPCI patients, usually within one week of the infarct. Mortality ranges between 50% and 87%. Diagnosis usually requires multi-modality imaging (echocardiography, ventriculography, computed tomography, CMR) before surgical repair. Because of its high spatial resolution, CMR can be used to clarify the detailed structure of these lesions. CMR accurately identifies the location, size and tissue margins of VSD and is useful for detecting apical defects, which are not easily identified by echocardiography. CMR measurements may also be used to determine the size of ventricular patch required to close the VSD, which avoids the inflation of a sizing balloon in friable infarcted tissue. **[8]** These features, together with other CMR markers of damage (e.g. infarct size, microvascular obstruction, left ventricular dysfunction) are useful for guiding optimal management of patients with post-infarct VSD.

4. <u>The following statements relate to the ability of CMR to identify patients with VSD after myocardial infarction.</u>

   a. CMR identifies the location and characteristics of post-infarct VSD better than echocardiography.

   Strongly disagree | Moderately disagree | Neither agree nor disagree | Moderately agree | Strongly agree

   b. Better identification of the location and characteristics of post-infarct VSD guides the optimal management of these patients.

   Strongly disagree | Moderately disagree | Neither agree nor disagree | Moderately agree | Strongly agree

   c. Optimal management of patients with post-infarct VSD is expected to lead to a reduced risk of MACE in the long term.

   Strongly disagree | Moderately disagree | Neither agree nor disagree | Moderately agree | Strongly agree

   d. Optimal management of patients with post-infarct VSD is expected to lead to less NHS resource use in the long term.

   Strongly disagree | Moderately disagree | Neither agree nor disagree | Moderately agree | Strongly agree

   e. The anticipated benefit (reduced risk of MACE and/or less NHS resource use) is likely to be sufficiently large to make CMR cost effective among the patients in whom it would be indicated.

   Strongly disagree | Moderately disagree | Neither agree nor disagree | Moderately agree | Strongly agree

For peer review only - http://bmjopen.bmj.com/site/about/guidelines.xhtml

Between 5% and 12% of patients who have an emergency angiogram are found to have unobstructed coronary arteries. In these patients, the lack of an accurate diagnosis may result in inappropriate or unnecessary treatment or follow-up and poorer prognosis. **[9]** CMR facilitates differential diagnosis in patients with unobstructed coronary arteries on angiography, providing a definitive diagnosis (e.g. myocardial infarction, myocarditis, Takotsubo cardiomyopathy) in 65-90% of these patients. **[10, 11]** In patients with myocarditis, late gadolinium enhancement (LGE) on CMR may predict long term adverse outcomes. **[12]** In the context of myocardial infarction without an angiographic lesion, CMR can locate the culprit infarct-related artery in patients with spontaneous reperfusion or with distal embolization.

5. <u>The following statements relate to the ability of CMR to differentiate myocaradial infarction from other diagnoses in patients found to have unobstructed coronary arteries on emergency angiography.</u>

   a. Unlike echocardiography, CMR can provide a definitive ischaemic diagnosis (e.g. myocardial infarction with spontaneous reperfusion or distal embolization) or a non-ischaemic diagnosis (e.g. myocarditis, Takotsubo cardiomyopathy, aortic dissection) in patients with unobstructed coronary arteries on angiography.

Strongly disagree	Moderately disagree	Neither agree nor disagree	Moderately agree	Strongly agree

   b. A definitive diagnosis results in a patient treatment plan appropriate for that diagnosis.

Strongly disagree	Moderately disagree	Neither agree nor disagree	Moderately agree	Strongly agree

   c. A treatment plan appropriate for the diagnosis is expected to lead to a reduced risk of MACE in the long term.

Strongly disagree	Moderately disagree	Neither agree nor disagree	Moderately agree	Strongly agree

   d. A treatment plan appropriate for the definitive diagnosis is expected to lead to less NHS resource use in the long term.

Strongly disagree	Moderately disagree	Neither agree nor disagree	Moderately agree	Strongly agree

   e. The anticipated benefit (reduced risk of MACE and/or less NHS resource use) is likely to be sufficiently large to make CMR cost effective among the patients in whom it would be indicated.

Strongly disagree	Moderately disagree	Neither agree nor disagree	Moderately agree	Strongly agree

About 6% of heart attack patients subsequently die suddenly from a presumed cardiac cause. Current guidelines recommend the use of an implantable cardioverter defibrillator (ICD) to prevent sudden cardiac death in patients who have a low left ventricular ejection fraction (EF) after a heart attack. EF is used in clinical practice to make decisions about ICD implantation, but it has a low predictive value and many patients with an ICD will never benefit from it. **[13]** EF is most commonly measured by echocardiography, but CMR is now considered the gold standard for EF measurement because it is more reproducible than echocardiography. **[1]** CMR has been shown to be better than echocardiography for selecting patients for ICD implantation when strict EF thresholds are used to guide implantation. **[14]** The extent of myocardial scar characterised by late gadolinium enhancement (LGE) CMR may also be used to predict whether ICD implantation is appropriate in this patient group. **[15]** Furthermore, LGE CMR can guide placement of the left ventricular lead away from scarred myocardium, which results in a better clinical outcome after cardiac resynchronisation therapy (CRT). **[16]**

6. <u>The following statements relate to the ability of CMR to identify patients at high risk of sudden cardiac death after PPCI who would benefit most from an implantable cardiac device.</u>

   a. CMR identifies PPCI patients who are at high risk of sudden cardiac death better than echocardiography.

   Strongly disagree | Moderately disagree | Neither agree nor disagree | Moderately agree | Strongly agree

   b. Better identification of PPCI patients at high risk of sudden cardiac death allows optimal patient selection for an implantable cardiac device (ICD or CRT).

   Strongly disagree | Moderately disagree | Neither agree nor disagree | Moderately agree | Strongly agree

   c. Optimal patient selection for an implantable cardiac device is expected to lead to a reduced risk of MACE in these patients in the long term.

   Strongly disagree | Moderately disagree | Neither agree nor disagree | Moderately agree | Strongly agree

   d. Optimal patient selection for an implantable cardiac device is expected to lead to less NHS resource use in the long term.

   Strongly disagree | Moderately disagree | Neither agree nor disagree | Moderately agree | Strongly agree

   e. The anticipated benefit (reduced risk of MACE and/or less NHS resource use) is likely to be sufficiently large to make CMR cost effective among the patients in whom it would be indicated.

   Strongly disagree | Moderately disagree | Neither agree nor disagree | Moderately agree | Strongly agree

Cardiac resynchronisation therapy (CRT), or biventricular pacing, uses a specialised pacemaker to improve the contraction of the left ventricle and the overall efficiency of the heart. It is used in patients with systolic ventricular dysfunction and heart failure. However, about 30% of patients who meet the inclusion criteria for CRT do not respond to it. **[17]** CMR assessment of mechanical dyssynchrony and myocardial scar provides additional value over echocardiography for identifying non responsive patients. **[18, 19]** Myocardial scar is an important feature of non-response to CRT **[20, 21]** and late gadolinium enhancement (LGE) CMR accurately differentiates between transmural, mid-myocardial, epicardial, and subendocardial scar.

7. <u>The following statements relate to the ability of CMR to identify patients who **would not** benefit from CRT after PPCI.</u>

   a. CMR identifies patients who would not benefit from CRT better than echocardiography.

Strongly disagree	Moderately disagree	Neither agree nor disagree	Moderately agree	Strongly agree

   b. The ability to identify patients who would not benefit from CRT would reduce CRT use in patients who do not need it.

Strongly disagree	Moderately disagree	Neither agree nor disagree	Moderately agree	Strongly agree

   c. Reducing CRT use in patients who do not need it is expected to lead to reduced risk of MACE in these patients in the long term.

Strongly disagree	Moderately disagree	Neither agree nor disagree	Moderately agree	Strongly agree

   d. Reducing CRT use in patients who do not need it is expected to lead to less NHS resource use in the long term.

Strongly disagree	Moderately disagree	Neither agree nor disagree	Moderately agree	Strongly agree

   e. The anticipated benefit (reduced risk of MACE and/or less NHS resource use) is likely to be sufficiently large to make CMR cost effective among the patients in whom it would be indicated.

Strongly disagree	Moderately disagree	Neither agree nor disagree	Moderately agree	Strongly agree

For peer review only - http://bmjopen.bmj.com/site/about/guidelines.xhtml

Between 40% and 65% of the patients who activate the PPCI pathway have multi-vessel disease. Adequate assessment of residual ischemia in non-culprit arteries post PPCI is important for effective management because this patient group has an adverse prognosis. Various techniques are currently used to assess the need for additional revascularisation, including stress echocardiography, single photon emission computed tomography (SPECT) and stress-perfusion CMR. Stress CMR has excellent prognostic value **[22]** and better diagnostic accuracy than SPECT **[23, 24]** or stress echocardiography **[25]** for detecting coronary artery disease causing significant regional myocardial ischaemia.

8. <u>The following statements relate to the ability of CMR to assess ischemia and viability in patients with multi-vessel disease.</u>

   a. CMR assesses ischemia and viability of the myocardium better than echocardiography.

Strongly disagree	Moderately disagree	Neither agree nor disagree	Moderately agree	Strongly agree

   b. Better assessment of ischemia and viability of the myocardium optimises the revascularisation strategy for patients with multi-vessel disease and avoids additional diagnostic tests.

Strongly disagree	Moderately disagree	Neither agree nor disagree	Moderately agree	Strongly agree

   c. The ability to optimise the revascularisation strategy for patients with multi-vessel disease is expected to lead to a reduced risk of MACE in the long term.

Strongly disagree	Moderately disagree	Neither agree nor disagree	Moderately agree	Strongly agree

   f. The ability to optimise the revascularisation strategy and avoid additional diagnostic tests in patients with multi-vessel disease is expected to lead to less NHS resource use in the long term.

Strongly disagree	Moderately disagree	Neither agree nor disagree	Moderately agree	Strongly agree

   d. The anticipated benefit (reduced risk of MACE and/or less NHS resource use) is likely to be sufficiently large to make CMR cost effective among the patients in whom it would be indicated.

Strongly disagree	Moderately disagree	Neither agree nor disagree	Moderately agree	Strongly agree

Left ventricular (LV) thrombus is a serious complication of acute myocardial infarction. It increases risk of thromboembolic events, particularly stroke. LV thrombus develops in up to 10% of patients with anterior wall infarction after PPCI. Although echocardiography is most commonly used to detect LV thrombus and to assess its shape and size, between 10-46% of echocardiograms are inconclusive. **[26]** Late gadolinium enhancement (LGE) CMR is considered the gold standard for detecting LV thrombus, because it detects thrombus based on tissue characteristics rather than anatomic appearance. Fewer thrombi are detected by contrast-echocardiography than by LGE CMR. **[27]** CMR has a higher sensitivity (88%) than contrast (61%) and noncontrast (<33%) echocardiography. **[27, 28]**

9. The following statements relate to the ability of CMR to identify patients with post-infarct LV thrombus.

   a. CMR identifies post-infarct LV thrombus better than echocardiography.

Strongly disagree	Moderately disagree	Neither agree nor disagree	Moderately agree	Strongly agree

   b. Better detection of post-infarct LV thrombus in PPCI patients allows more affected patients to be treated with anticoagulation therapy.

Strongly disagree	Moderately disagree	Neither agree nor disagree	Moderately agree	Strongly agree

   c. Treatment with anticoagulation therapy in patients with post-infarct LV thrombus is expected to lead to a reduced risk of MACE in the long term.

Strongly disagree	Moderately disagree	Neither agree nor disagree	Moderately agree	Strongly agree

   d. Treatment with anticoagulation therapy in patients with post-infarct LV thrombus is expected to lead to less NHS resource use in the long term.

Strongly disagree	Moderately disagree	Neither agree nor disagree	Moderately agree	Strongly agree

   e. The anticipated benefit (reduced risk of MACE and/or less NHS resource use) is likely to be sufficiently large to make CMR cost effective among the patients in whom it would be indicated.

Strongly disagree	Moderately disagree	Neither agree nor disagree	Moderately agree	Strongly agree

For peer review only

Compared with echocardiography, CMR provides versatile imaging planes, superior tissue contrast and advanced tissue characterisation, allowing a comprehensive assessment of cardiac anatomy, function and flow, and imaging of the great vessels (including venous return), the pericardium and suspected cardiac tumours. **[29]** As such, CMR can identify congenital coronary anomalies, cardiac masses, coronary artery aneurysms, valvular heart disease, thoracic aortic disease, etc. CMR can adequately differentiate benign from malignant tumours in the heart. **[30]** Unlike echocardiography, which provides limited imaging of mediastinal and extra-cardiac structures, CMR also images a substantial part of the thorax and abdomen in the field of view, which may potentially contain non-cardiac abnormalities. The prevalence of non-cardiac findings on CMR is up to 80% (with up to 30% of these representing potentially significant findings), depending on the characteristics of the population examined. **[31-33]**

10. <u>The following statements relate to the ability of CMR to detect incidental cardiac and non-cardiac findings if offered routinely to patients who undergo an emergency angiogram.</u>

    a. CMR identifies more incidental cardiac / non-cardiac findings than echocardiography.

Strongly disagree	Moderately disagree	Neither agree nor disagree	Moderately agree	Strongly agree

    b. Improved detection of potentially significant incidental findings allows affected patients to be investigated further and / or treated.

Strongly disagree	Moderately disagree	Neither agree nor disagree	Moderately agree	Strongly agree

    c. Further investigation and treatment is expected to reduce the risk of MACE / increase overall survival in affected patients in the long term.

Strongly disagree	Moderately disagree	Neither agree nor disagree	Moderately agree	Strongly agree

    d. Further investigation and treatment is expected to lead to less NHS resource use in the long term.

Strongly disagree	Moderately disagree	Neither agree nor disagree	Moderately agree	Strongly agree

    e. The anticipated benefit (reduced risk of MACE and/or less NHS resource use) is likely to be sufficiently large to make CMR cost effective among the patients who undergo an emergency angiogram.

Strongly disagree	Moderately disagree	Neither agree nor disagree	Moderately agree	Strongly agree

References

1. Dorosz JL, Lezotte DC, Weitzenkamp DA, Allen LA, Salcedo EE: **Performance of 3-dimensional echocardiography in measuring left ventricular volumes and ejection fraction: a systematic review and meta-analysis**. *J Am Coll Cardiol* 2012, **59**(20):1799-1808.
2. Eitel I, Desch S, de WS, Fuernau G, Gutberlet M, Schuler G, Thiele H: **Long-term prognostic value of myocardial salvage assessed by cardiovascular magnetic resonance in acute reperfused myocardial infarction**. *Heart* 2011, **97**(24):2038-2045.
3. Eitel I, Desch S, Fuernau G, Hildebrand L, Gutberlet M, Schuler G, Thiele H: **Prognostic significance and determinants of myocardial salvage assessed by cardiovascular magnetic resonance in acute reperfused myocardial infarction**. *J Am Coll Cardiol* 2010, **55**(22):2470-2479.
4. Klug G, Mayr A, Schenk S, Esterhammer R, Schocke M, Nocker M, Jaschke W, Pachinger O, Metzler B: **Prognostic value at 5 years of microvascular obstruction after acute myocardial infarction assessed by cardiovascular magnetic resonance**. *Journal of Cardiovascular Magnetic Resonance* 2012, **14**:46.
5. Wong TC, Piehler K, Puntil KS, Moguillansky D, Meier CG, Lacomis JM, Kellman P, Cook SC, Schwartzman DS, Simon MA *et al*: **Effectiveness of late gadolinium enhancement to improve outcomes prediction in patients referred for cardiovascular magnetic resonance after echocardiography**. *Journal of cardiovascular magnetic resonance : official journal of the Society for Cardiovascular Magnetic Resonance* 2013, **15**:6.
6. Parsai C, O'Hanlon R, Prasad SK, Mohiaddin RH: **Diagnostic and prognostic value of cardiovascular magnetic resonance in non-ischaemic cardiomyopathies**. *Journal of cardiovascular magnetic resonance : official journal of the Society for Cardiovascular Magnetic Resonance* 2012, **14**:54.
7. Green JJ, Berger JS, Kramer CM, Salerno M: **Prognostic value of late gadolinium enhancement in clinical outcomes for hypertrophic cardiomyopathy**. *JACC Cardiovascular imaging* 2012, **5**(4):370-377.
8. Artis NJ, Thomson J, Plein S, Greenwood JP: **Percutaneous closure of postinfarction ventricular septal defect: cardiac magnetic resonance-guided case selection and postprocedure evaluation**. *The Canadian journal of cardiology* 2011, **27**(6):869 e863-865.
9. Dokainish H, Pillai M, Murphy SA, DiBattiste PM, Schweiger MJ, Lotfi A, Morrow DA, Cannon CP, Braunwald E, Lakkis N: **Prognostic implications of elevated troponin in patients with suspected acute coronary syndrome but no critical epicardial coronary disease: a TACTICS-TIMI-18 substudy**. *J Am Coll Cardiol* 2005, **45**(1):19-24.
10. Assomull RG, Lyne JC, Keenan N, Gulati A, Bunce NH, Davies SW, Pennell DJ, Prasad SK: **The role of cardiovascular magnetic resonance in patients presenting with chest pain, raised troponin, and unobstructed coronary arteries**. *Eur Heart J* 2007, **28**(10):1242-1249.
11. Leurent G, Langella B, Fougerou C, Lentz PA, Larralde A, Bedossa M, Boulmier D, Le Breton H: **Diagnostic contributions of cardiac magnetic resonance imaging in patients presenting with elevated troponin, acute chest pain syndrome and unobstructed coronary arteries**. *Arch Cardiovasc Dis* 2011, **104**(3):161-170.
12. Grun S, Schumm J, Greulich S, Wagner A, Schneider S, Bruder O, Kispert EM, Hill S, Ong P, Klingel K *et al*: **Long-term follow-up of biopsy-proven viral myocarditis: predictors of mortality and incomplete recovery**. *J Am Coll Cardiol* 2012, **59**(18):1604-1615.
13. Moss AJ, Greenberg H, Case RB, Zareba W, Hall WJ, Brown MW, Daubert JP, McNitt S, Andrews ML, Elkin AD *et al*: **Long-term clinical course of patients after termination of ventricular tachyarrhythmia by an implanted defibrillator**. *Circulation* 2004, **110**(25):3760-3765.
14. Joshi SB, Connelly KA, Jimenez-Juan L, Hansen M, Kirpalani A, Dorian P, Mangat I, Al-Hesayen A, Crean AM, Wright GA *et al*: **Potential clinical impact of cardiovascular magnetic**

For peer review only

resonance assessment of ejection fraction on eligibility for cardioverter defibrillator implantation. *Journal of cardiovascular magnetic resonance : official journal of the Society for Cardiovascular Magnetic Resonance* 2012, **14**:69.

15. Alexandre J, Saloux E, Dugue AE, Lebon A, Lemaitre A, Roule V, Labombarda F, Provost N, Gomes S, Scanu P *et al*: **Scar extent evaluated by late gadolinium enhancement CMR: a powerful predictor of long term appropriate ICD therapy in patients with coronary artery disease**. *Journal of cardiovascular magnetic resonance : official journal of the Society for Cardiovascular Magnetic Resonance* 2013, **15**:12.
16. Leyva F, Foley PW, Chalil S, Ratib K, Smith RE, Prinzen F, Auricchio A: **Cardiac resynchronization therapy guided by late gadolinium-enhancement cardiovascular magnetic resonance**. *Journal of cardiovascular magnetic resonance : official journal of the Society for Cardiovascular Magnetic Resonance* 2011, **13**:29.
17. Leclercq C, Kass DA: **Retiming the failing heart: principles and current clinical status of cardiac resynchronization**. *J Am Coll Cardiol* 2002, **39**(2):194-201.
18. Bilchick KC, Dimaano V, Wu KC, Helm RH, Weiss RG, Lima JA, Berger RD, Tomaselli GF, Bluemke DA, Halperin HR *et al*: **Cardiac magnetic resonance assessment of dyssynchrony and myocardial scar predicts function class improvement following cardiac resynchronization therapy**. *JACC Cardiovascular imaging* 2008, **1**(5):561-568.
19. Ellims AH, Pfluger H, Elsik M, Butler MJ, Hare JL, Taylor AJ: **Utility of cardiac magnetic resonance imaging, echocardiography and electrocardiography for the prediction of clinical response and long-term survival following cardiac resynchronisation therapy**. *Int J Cardiovasc Imaging* 2013, **29**(6):1303-1311.
20. Adelstein EC, Saba S: **Scar burden by myocardial perfusion imaging predicts echocardiographic response to cardiac resynchronization therapy in ischemic cardiomyopathy**. *Am Heart J* 2007, **153**(1):105-112.
21. White JA, Yee R, Yuan X, Krahn A, Skanes A, Parker M, Klein G, Drangova M: **Delayed enhancement magnetic resonance imaging predicts response to cardiac resynchronization therapy in patients with intraventricular dyssynchrony**. *J Am Coll Cardiol* 2006, **48**(10):1953-1960.
22. Lipinski MJ, McVey CM, Berger JS, Kramer CM, Salerno M: **Prognostic value of stress cardiac magnetic resonance imaging in patients with known or suspected coronary artery disease: a systematic review and meta-analysis**. *J Am Coll Cardiol* 2013, **62**(9):826-838.
23. Greenwood JP, Maredia N, Younger JF, Brown JM, Nixon J, Everett CC, Bijsterveld P, Ridgway JP, Radjenovic A, Dickinson CJ *et al*: **Cardiovascular magnetic resonance and single-photon emission computed tomography for diagnosis of coronary heart disease (CE-MARC): a prospective trial**. *Lancet* 2012, **379**(9814):453-460.
24. Schwitter J, Wacker CM, Wilke N, Al-Saadi N, Sauer E, Huettle K, Schonberg SO, Debl K, Strohm O, Ahlstrom H *et al*: **Superior diagnostic performance of perfusion-cardiovascular magnetic resonance versus SPECT to detect coronary artery disease: The secondary endpoints of the multicenter multivendor MR-IMPACT II (Magnetic Resonance Imaging for Myocardial Perfusion Assessment in Coronary Artery Disease Trial)**. *Journal of cardiovascular magnetic resonance : official journal of the Society for Cardiovascular Magnetic Resonance* 2012, **14**:61.
25. Nagel E, Lehmkuhl HB, Bocksch W, Klein C, Vogel U, Frantz E, Ellmer A, Dreysse S, Fleck E: **Noninvasive diagnosis of ischemia-induced wall motion abnormalities with the use of high-dose dobutamine stress MRI: comparison with dobutamine stress echocardiography**. *Circulation* 1999, **99**(6):763-770.
26. Delewi R, Zijlstra F, Piek JJ: **Left ventricular thrombus formation after acute myocardial infarction**. *Heart* 2012, **98**(23):1743-1749.
27. Weinsaft JW, Kim RJ, Ross M, Krauser D, Manoushagian S, LaBounty TM, Cham MD, Min JK, Healy K, Wang Y *et al*: **Contrast-enhanced anatomic imaging as compared to contrast-**

For peer review only

enhanced tissue characterization for detection of left ventricular thrombus. *JACC Cardiovascular imaging* 2009, **2**(8):969-979.

28. Srichai MB, Junor C, Rodriguez LL, Stillman AE, Grimm RA, Lieber ML, Weaver JA, Smedira NG, White RD: **Clinical, imaging, and pathological characteristics of left ventricular thrombus: a comparison of contrast-enhanced magnetic resonance imaging, transthoracic echocardiography, and transesophageal echocardiography with surgical or pathological validation**. *Am Heart J* 2006, **152**(1):75-84.
29. American College of Cardiology Foundation Task Force on Expert Consensus D, Hundley WG, Bluemke DA, Finn JP, Flamm SD, Fogel MA, Friedrich MG, Ho VB, Jerosch-Herold M, Kramer CM *et al*: **ACCF/ACR/AHA/NASCI/SCMR 2010 expert consensus document on cardiovascular magnetic resonance: a report of the American College of Cardiology Foundation Task Force on Expert Consensus Documents**. *Circulation* 2010, **121**(22):2462-2508.
30. Hoffmann U, Globits S, Schima W, Loewe C, Puig S, Oberhuber G, Frank H: **Usefulness of magnetic resonance imaging of cardiac and paracardiac masses**. *The American journal of cardiology* 2003, **92**(7):890-895.
31. Chan PG, Smith MP, Hauser TH, Yeon SB, Appelbaum E, Rofsky NM, Manning WJ: **Noncardiac pathology on clinical cardiac magnetic resonance imaging**. *JACC Cardiovascular imaging* 2009, **2**(8):980-986.
32. McKenna DA, Laxpati M, Colletti PM: **The prevalence of incidental findings at cardiac MRI**. *The open cardiovascular medicine journal* 2008, **2**:20-25.
33. Wyttenbach R, Medioni N, Santini P, Vock P, Szucs-Farkas Z: **Extracardiac findings detected by cardiac magnetic resonance imaging**. *Eur Radiol* 2012, **22**(6):1295-1302.

For peer review only

Appendix 3. Literature search

Medline (1950 to Jan 2014)

1 exp Myocardial Infarction/ (142973)
2 myocardial infarct$.tw. (134547)
3 heart attack$.tw. (3601)
4 MI.tw. (25881)
5 ami.tw. (12166)
6 stemi.tw. (3663)
7 heart infarct$.tw. (705)
8 Acute Coronary Syndrome/ (6784)
9 acute coronary syndrome$.tw. (15521)
10 ACS.tw. (9320)
11 or/1-10 (210783)
12 exp angioplasty/ (53568)
13 angioplasty.tw. (34003)
14 percutaneous coronary intervention$.tw. (14406)
15 exp Percutaneous Coronary Intervention/ (34668)
16 PCI.tw. (11157)
17 PPCI.tw. (366)
18 exp Stents/ (50784)
19 stent$.tw. (56427)
20 Myocardial Revascularization/ (9079)
21 revasculari$.tw. (37853)
22 reperfused.tw. (5688)
23 reperfusion.tw. (55775)
24 or/12-23 (191056)
25 exp Magnetic Resonance Imaging/ (295774)
26 Magnetic resonance imag$.tw. (122420)
27 Cardiovascular magnetic resonance.tw. (1765)
28 cardiac magnetic resonance.tw. (3179)
29 MRI.tw. (121901)
30 CMR.tw. (3035)
31 or/25-30 (344849)
32 24 and 31 (5713)
33 11 and 32 (1318)
34 exp animals/ not humans/ (3863199)
35 33 not 34 (1051)

Embase (1980 to Jan 2014)

1 exp heart infarction/ (261299)
2 myocardial infarct$.tw. (185732)
3 heart attack$.tw. (4991)
4 MI.tw. (45694)
5 ami.tw. (19697)
6 stemi.tw. (10463)
7 heart infarct$.tw. (1450)
8 exp Acute Coronary Syndrome/ (26244)
9 acute coronary syndrome$.tw. (28004)
10 ACS.tw. (17708)
11 or/1-10 (337561)
12 exp angioplasty/ (67762)
13 angioplasty.tw. (47179)

For peer review only

14 percutaneous coronary intervention$.tw. (27203)
15 exp Percutaneous Coronary Intervention/ (59160)
16 PCI.tw. (26664)
17 PPCI.tw. (1281)
18 Stent/ (64591)
19 exp cardiovascular stent/ (34380)
20 stent$.tw. (94492)
21 heart muscle revascularization/ (21848)
22 revasculari$.tw. (56445)
23 reperfused.tw. (7304)
24 reperfusion.tw. (77453)
25 or/12-24 (298341)
26 exp nuclear magnetic resonance imaging/ (524106)
27 Magnetic resonance imag$.tw. (164672)
28 Cardiovascular magnetic resonance.tw. (3414)
29 cardiac magnetic resonance.tw. (6341)
30 MRI.tw. (203793)
31 CMR.tw. (7673)
32 or/26-31 (563445)
33 25 and 32 (13408)
34 11 and 33 (3451)
35 (exp animal/ or nonhuman/) not exp humans/ (5173875)
36 34 not 35 (3048)
37 limit 36 to embase (2864)

The Cochrane Library

#1 MeSH descriptor: [Myocardial Infarction] explode all trees
#2 MeSH descriptor: [Acute Coronary Syndrome] explode all trees
#3 myocardial next infarct*
#4 heart next attack*
#5 MI
#6 AMI
#7 STEMI
#8 heart next infarct*
#9 "acute coronary syndrome*"
#10 #1 or #2 or #3 or #4 or #5 or #6 or #7 or #8 or #9
#11 MeSH descriptor: [Angioplasty] explode all trees
#12 MeSH descriptor: [Percutaneous Coronary Intervention] explode all trees
#13 MeSH descriptor: [Stents] explode all trees
#14 MeSH descriptor: [Myocardial Revascularization] this term only
#15 angioplasty
#16 "percutaneous coronary intervention*"
#17 PCI or PPCI
#18 stent*
#19 revasculari*
#20 reperfused
#21 reperfusion
#22 #11 or #12 or #13 or #14 or #15 or #16 or #17 or #18 or #19 or #20 or #21
#23 MeSH descriptor: [Magnetic Resonance Imaging] explode all trees
#24 "Magnetic resonance imag*"
#25 " Cardiovascular magnetic resonance"
#26 "cardiac magnetic resonance"
#27 MRI or CMR
#28 #23 or #24 or #25 or #26 or #27

For peer review only

#29 #11 and #22 and #28

ISI Web of Science

4 1,351 #3 AND #2 AND #1
3 218,389 TOPIC: (Angioplasty or “percutaneous coronary intervention*” or PCI or PPCI or stent* or revasulari* or reperfused or reperfusion)
2 257,465 TOPIC: (“Magnetic Resonance Imag*” or “Cardiovascular magnetic resonance” or “ cardiac magnetic resonance” or MRI or CMR)
1 273,481 TOPIC: (“myocardial infarct*” or “heart attack*” or “heart infarct*” or “acute coronary syndrome*” or MI or AMI or STEMI or ACS)

BIOSIS

6 873 #5 AND #4
5 TAXONOMIC DATA: (human not (animal* not human))
4 #3 AND #2 AND #1
3 TOPIC: (Angioplasty or “percutaneous coronary intervention*” or PCI or PPCI or stent* or revasulari* or reperfused or reperfusion)
2 TOPIC: (“Magnetic Resonance Imag*” or “Cardiovascular magnetic resonance” or “ cardiac magnetic resonance” or MRI or CMR)
1 TOPIC: (“myocardial infarct*” or “heart attack*” or “heart infarct*” or “acute coronary syndrome*” or MI or AMI or STEMI or ACS)

For peer review only

BMJ Open

Formal consensus to identify clinically important changes in management resulting from the use of cardiovascular magnetic resonance (CMR) patients who activate the primary percutaneous coronary intervention (PPCI) pathway

Journal:	*BMJ Open*
Manuscript ID	bmjopen-2016-014627.R1
Article Type:	Research
Date Submitted by the Author:	23-Mar-2017

Complete List of Authors:	Pufulete, Maria; University of Bristol,<br>Brierley, Rachel; University of Bristol<br>Bucciarelli Ducci, Chiara; University of Bristol<br>Greenwood, John; Leeds General Infirmary, Cardiology<br>Dorman, Stephen; University Hospitals Bristol NHS Foundation Trust<br>Anderson, Richard; University Hospital of Wales, Cardiology<br>Harris, Jessica; University of Bristol School of Clinical Science<br>MacAlindon, Elisa; New Cross Hospital<br>rogers, chris; university of bristol, Clinical Science South Bristol<br>Reeves, Barnaby; University of Bristol, Bristol Heart Institute

**Primary Subject Heading**:	Cardiovascular medicine
Secondary Subject Heading:	Diagnostics, Health services research, Medical management, Radiology and imaging, Research methods
Keywords:	primary percutaneous coronary intervention, systematic review, formal consensus, Cardiovascular magnetic resonance imaging

SCHOLARONE™
Manuscripts

Formal consensus to identify clinically important changes in management resulting from the use of cardiovascular magnetic resonance (CMR) patients who activate the primary percutaneous coronary intervention (PPCI) pathway

Maria Pufulete[1], Rachel Brierley[1], Chiara Bucciarelli-Ducci[2], John P. Greenwood[3], Stephen Dorman[2], Richard A Anderson[4], Jessica Harris[1], Elisa McAlindon[5], Chris A Rogers[1], Barnaby C Reeves[1]*

[1] Clinical Trials and Evaluation Unit, University of Bristol, Level 7, Bristol Royal Infirmary, Queen's Building, Bristol, United Kingdom.

[2] NIHR Bristol Cardiovascular Research Unit, Bristol Heart Institute, University Hospitals Bristol NHS Foundation Trust, United Kingdom.

[3] Multidisciplinary Cardiovascular Research Centre and Leeds Institute of Cardiovascular and Metabolic Medicine, University of Leeds, United Kingdom.

[4] University Hospitals of Wales, Heath Park, Cardiff, United Kingdom.

[5] Department of Cardiology, New Cross Hospital, Wolverhampton, United Kingdom.

[*] Correspondence to: Maria Pufulete, Clinical Trials and Evaluation Unit, University of Bristol, Level 7, Bristol Royal Infirmary, Queen's Building, Bristol, United Kingdom. Tel. +44 117 3422526. Fax. +44 117 3423288. Email: maria.pufulete@bristol.ac.uk

Abbreviations

ACS, acute coronary syndrome; CI, confidence interval; CMR, cardiovascular magnetic resonance; IQ, interquartile range; IS, infarct size; LV, left ventricular; MACE, major adverse cardiovascular events; MI, myocardial infarction; MVD, multi-vessel disease; MVO, microvascular obstruction; NSTEMI, non ST-elevation myocardial infarction; OHCA, out of hospital cardiac arrest; PPCI, primary percutaneous coronary intervention; SPECT, single photon emission computed tomography; STEMI, ST-elevation myocardial infarction.

For peer review only - http://bmjopen.bmj.com/site/about/guidelines.xhtml

For peer review only

Key words: Cardiovascular magnetic resonance, primary percutaneous coronary intervention, systematic review, formal consensus.

Word count: approx. 2089 (not including abstract, strengths and limitations, contributions, figure and tables or titles)

Number of tables: 2

Number of figures: 1

For peer review only

For peer review only - http://bmjopen.bmj.com/site/about/guidelines.xhtml

Abstract

Objective

To define important changes in management arising from the use of cardiovascular magnetic resonance (CMR) in patients who activate the primary percutaneous coronary intervention (PPCI) pathway.

Design

Formal consensus study using literature review and cardiologist expert opinion to formulate consensus statements and setting up a consensus panel to review the statements (by completing a web-based survey, attending a face-to-face meeting to discuss survey results and modify the survey to reflect group discussion, and completing the modified survey to determine which statements were in consensus).

Participants

Formulation of consensus statements: four cardiologists (two CMR, two interventional) and six non-clinical researchers. Formal consensus: seven cardiologists (two CMR, three interventional, one echocardiography and one heart failure). Forty nine additional cardiologists completed the modified survey.

Results

Thirty seven draft statements describing changes in management following CMR were generated; these were condensed into 12 statements and reviewed through the formal consensus process. Three of 12 statements were classified in consensus in the first survey; these related to the role of CMR in: identifying the cause of out-of-hospital cardiac arrest; providing a definitive diagnosis in patients found to have unobstructed arteries on angiography; and identifying patients with left ventricular (LV) thrombus. Two additional statements were in consensus in the modified survey, relating to the ability of CMR to: identify patients who have a poor prognosis after PPCI; and assess ischemia and viability in patients with multi-vessel disease.

Conclusion

For peer review only - http://bmjopen.bmj.com/site/about/guidelines.xhtml

For peer review only

There was consensus that CMR leads to clinically important changes in management in five subgroups of patients who activate the PPCI pathway.

For peer review only

For peer review only - http://bmjopen.bmj.com/site/about/guidelines.xhtml

Strengths and limitations of this study

- We used formal consensus methods to identify potentially important changes in management arising from the use of cardiac magnetic resonance (CMR) in patients who activate the PPCI pathway.
- We identified five patient subgroups in this population in which CMR leads to clinically important changes in management.
- We did not include different stakeholder groups in the consensus panel as the technical wording of the statements would not have been easily understood by non-cardiologists.

For peer review only

For peer review only - http://bmjopen.bmj.com/site/about/guidelines.xhtml

Introduction

There has been rapid uptake of cardiovascular magnetic resonance (CMR) in the UK, with a 44% increase in the number of scans per centre (from 557 to 802) between 2008 and 2010 [1]. Despite the rapid increase in the number of scans undertaken there is limited evidence about the impact of CMR on long term prognosis or treatment decisions. CMR is increasingly being used for patients who activate the primary percutaneous coronary intervention (PPCI) pathway, but it is not clear how CMR influences clinical management in this population.

We conducted a study to determine the feasibility of setting up a prospective registry using routine data linkage to assess the clinical and cost-effectiveness of CMR in patients who activate the PPCI pathway [2]. A long term objective of the registry is to compare the incidence of major adverse cardiovascular events (MACE) in patients who do or do not have CMR after the index event. However, given the rare occurrence of such events in these patients, the study would have to accrue over 37,000 subjects to detect a clinically important reduction in the incidence of MACE with adequate power. Therefore, a key objective of the feasibility study was to define a primary composite outcome, acceptable to cardiologists and other stakeholders (e.g. clinical commissioners) as representing a clinically important change in management as a result of an eligible patient having had CMR (e.g. expected to prevent future MACE), that could be used for the registry in the medium term.

We used a formal consensus method based on the modified nominal group technique [3] to identify important changes in management (and the specific patient subgroups these changes in management relate to) that can be used to define the composite outcome. Formal consensus is a method that combines both research evidence and expert opinion. We used this approach because we knew from a preliminary scoping of the literature that there were few studies that reported the impact of CMR on patient management. There were three components to the consensus process: (i) a literature review to identify evidence about the use of CMR in patients who activate the PPCI pathway; (ii) formulation of statements about how CMR changes patient management using research evidence and

For peer review only - http://bmjopen.bmj.com/site/about/guidelines.xhtml

expert opinion; and (iii) the formal consensus involving an independent panel of cardiologists using surveys and discussion to reach consensus.

Methods

Literature review and formulation of the consensus statements

An initial working group was convened including two cardiologists with CMR expertise, two interventional cardiologists from sites participating in the feasibility study, one cardiac network director, two methodologists, two statisticians, one health economist and the study manager. We conducted a literature review to search for studies reporting the impact of CMR on prognosis, patient management, and risk stratification in the population of patients who activate the PPCI pathway. We searched without restriction by study design or search terms related to outcomes, so that we could determine the full extent of the literature in this area and identify all studies that used CMR in our population. The search was conducted on Medline, EMBASE, The Cochrane Library, ISI Web of Science (Citations Index and Proceedings) and BIOSIS (see **Appendix 1**). The following search terms were used, both as free text and Medical Subject Headings where possible: ‘acute coronary syndrome’, ‘myocardial infarction’, ‘angioplasty’, ‘percutaneous coronary intervention’, ‘cardiovascular magnetic resonance. We applied no restriction on publication date or language.

Draft statements were generated independently by three of the cardiologists (based on clinical expertise) and by one methodologist (based on evidence from the systematic literature search). Three members of the working group (study manager, systematic reviewer and cardiologist with CMR expertise) collated the statements, organised them according to patient subgroup, and standardised the wording of each statement. A one day meeting was organised for all members of the working group to consider the relevance, format and wording of each statement.

Survey design

For peer review only - http://bmjopen.bmj.com/site/about/guidelines.xhtml

Statements and supporting paragraphs were worded in a consistent manner and collated in the form of a web-based survey (SurveyMonkey, Palo Alto, California, USA; **Appendix 2**). The survey included an introductory page explaining the purpose and layout of the survey and instructions about how to complete it. Each statement followed with its supporting paragraph containing links to PDF references. Each statement was accompanied by a 9 point Likert scale asking the respondent to indicate whether he/she agreed with the statement or not (with 1 indicating “completely disagree” and 9 “completely agree”). There was also a free text box for each statement for respondents to comment and justify their score.

Establishing the expert panel

Clinicians in the working group identified consultant cardiologists with CMR, interventional, echocardiography, electrophysiology and heart failure expertise from across the UK. The cardiologists were invited by email to form an expert consensus panel.

Expert panel: completion of first survey

The expert panel completed the survey independently. Responses were collated and analysed by members of the working group.

Expert panel: face-to-face meeting

The expert panel attended a face-to-face meeting, chaired by a non-cardiology clinician experienced in facilitating formal consensus panels. The meeting was also attended by non-clinical members of the working group, whose role was to introduce the study, describe the structure of the formal consensus process, provide study related information and take minutes of the meeting. The expert panel discussed each statement and anonymised responses to the first survey in turn and agreed on modifications to the survey.

For peer review only - http://bmjopen.bmj.com/site/about/guidelines.xhtml

For peer review only

Expert panel: completion of the modified survey

The survey was modified by members of the working group as agreed in the face-to-face meeting (**Appendix 3**). The expert panel completed the modified survey independently and rated the statements a second time.

Extension of survey to other UK cardiologists

The survey was extended to other UK cardiologists through the British Cardiovascular Society, which advertised the survey in their monthly newsletter to members (over two consecutive months).

Criteria for consensus

Data for each statement are shown as median and interquartile (IQ) range. A median score of ≥and IQ range 6-9 was considered to be in agreement, or consensus, that the change in management described by the statement was clinically important. These statements were used to identify the patient subgroups perceived to benefit from CMR and define the treatment / process outcome that constitutes a definitive management change as a result of having CMR. A median score of ≤3 and IQ range 1-3 was considered to be in consensus that the statement did not constitute a clinically important change in management. Data were analysed using Stata/IC (V14, StataCorp LP, Texas, USA).

For peer review only

Results

Literature review and formulation of consensus statements

Thirty seven draft statements were generated by the three cardiologists and one methodologist. The literature search identified a total of 171 studies reporting the use of CMR in patients with acute myocardial infarction (MI) who activated the PPCI pathway. There were no studies that directly compared groups of patients having CMR or not with respect to patient management or clinical outcomes in this population. Statements relating to the same patient subgroup/condition (e.g. patients at risk of complications after PPCI who

For peer review only - http://bmjopen.bmj.com/site/about/guidelines.xhtml

develop left ventricular thrombus) were condensed into one statement. Participants at the working group combined some statements to avoid repetition and clarified the care pathway without CMR compared to which the additional benefit of CMR was anticipated, e.g. alternative imaging modalities such as echocardiography or single-photon emission computed tomography (SPECT)). This process resulted in twelve statements (**Appendix 3**) describing changes in management relating to six patient subgroups:

i. Patients with poor prognosis or at risk of complications after PPCI (five statements);
ii. Patients with good prognosis after PPCI who could be discharged earlier and followed up less often (one statement);
iii. Patients with multi-vessel disease (MVD) (two statements);
iv. Patients with unobstructed arteries on angiography (one statement);
v. Patients with out-of-hospital-cardiac arrest (OHCA) (one statement);
vi. Patients with incidental cardiac and extra-cardiac findings (two statements).

The expert panel

Nineteen consultant cardiologists were invited to participate in the consensus process. Seven cardiologists (37% of those invited) agreed to participate. Of these, two had CMR, three had interventional, one had echocardiography and one had heart failure expertise. All seven cardiologists completed the first survey independently.

First survey

There was consensus for three of the 12 statements (25%) that the change in management was clinically important (**Appendix 4**): the ability of CMR to identify the cause of OHCA and therefore optimise treatment for the patient; the ability of CMR to provide a definitive diagnosis in patients found to have unobstructed arteries on angiography; and the ability of CMR to identify patients with left ventricular (LV) thrombus and initiate treatment with anticoagulation therapy. There was no consensus for any statement that the change in management was not clinically important.

For peer review only - http://bmjopen.bmj.com/site/about/guidelines.xhtml

Consensus was sought on the importance of the change in management and most respondents commented on this issue. However, some respondents also considered other factors when rating statements, for example, the quality of the supporting evidence, the proportion of patients likely to benefit, the ability of the NHS to provide a service in line with the statement, and whether the cost of CMR justified the perceived benefit.

Face-to-face meeting

The face-to-face meeting was attended by six of the seven cardiologists and three non-clinical members of the initial working group. As a result of the face-to-face meeting, the survey was modified as follows:

i) The number of statements was reduced from 12 to 10. One statement (relating to patients with MVD) was removed because cardiologists felt that it overlapped significantly with a second statement relating to patients with MVD. Statements relating to incidental cardiac and extra-cardiac findings were combined into one statement.

ii) Respondents were asked to rate five aspects of each statement, organised hierarchically: (a) whether CMR is better than the comparator (e.g. echocardiography); (b) whether the information from CMR leads to a change in management; (c) whether the change in management is clinically important (i.e. likely to reduce risk of MACE in the long term); (d) whether the change in management is likely to reduce NHS costs in the long term; (e) whether the anticipated benefit was sufficiently large to make CMR cost effective among the patients in whom it would be indicated (see **Appendix 3**). Consensus was based on the distribution of responses to questions (a)-(c) of each statement [i.e. median ≥7, IQ range 6-9 for clinically important changes in management and median ≤ 3 and IQ range 1-3 for (a), (b) and (c)]. Responses to questions (a)-(c) were used because (a) was constructed to interrogate the respondent's appraisal of the evidence, (b) was constructed to determine whether the respondent believed the NHS service delivery changed as a result of CMR and (c) was

For peer review only - http://bmjopen.bmj.com/site/about/guidelines.xhtml

constructed to determine whether the respondent believed that any change recognised in (b) was clinically important. Discussions in the meeting revealed that consideration of costs and cost-effectiveness for the NHS influenced some panel member's responses to the initial survey. Questions (d) and (e) were added to isolate these aspects of consideration and separate them from the question of change in management, which was the aim of the consensus process.

iii) The free text box in which respondents could justify their score was removed.

The modified survey is shown in **Table 1**.

Table 1 Consensus statements (modified survey)

Statement	
1	The following statements relate to the ability of CMR to identify patients who have a poor prognosis after PPCI.<br>a. CMR markers (e.g. impaired left ventricular function, large infarct size, microvascular obstruction) better identify patients with a poor prognosis after PPCI than markers based on echocardiography.<br>b. Better identification of patients with a poor prognosis after PPCI allows these patients to be followed up more appropriately and treated more aggressively.<br>c. More appropriate follow up and more aggressive treatment in these patients is expected to lead to a reduced risk of MACE in the long term.
2	The following statements relate to the ability of CMR to identify patients who have a good prognosis after PPCI.<br>a. CMR markers (e.g. normal left ventricular function, high myocardial salvage, no microvascular obstruction, no residual ischemia) better identify patients with a good prognosis after PPCI than markers based on echocardiography.<br>b. Better identification of patients with a good prognosis after PPCI allows these patients to be followed up less frequently.<br>c. Less frequent follow up in these patients is expected to lead to less NHS resource use in the long term.
3	The following statements relate to the ability of CMR to identify the causes of OHCA in patients who undergo an emergency angiogram.<br>a. CMR better identifies the cause of OHCA (e.g. large myocardial infarction, ARVC, aberrant coronary arteries, HCM) than echocardiography.<br>b. Better identification of the cause of out-of-hospital cardiac arrest allows treatment to be optimised for these patients (e.g. defibrillator for primary arrhythmia or percutaneous coronary intervention) or their family members (e.g. genetic screening and counselling, primary prevention).<br>c. The ability to optimise treatment for these patients or family members is expected to lead to a reduced risk of MACE in the long term.
4	The following statements relate to the ability of CMR to identify patients with VSD after myocardial infarction.<br>a. CMR identifies the location and characteristics of post-infarct VSD better than echocardiography.<br>b. Better identification of the location and characteristics of post-infarct VSD guides the optimal management of these patients.<br>c. Optimal management of patients with post-infarct VSD is expected to lead to a reduced risk of MACE in the long term.
5	The following statements relate to the ability of CMR to differentiate myocardial infarction from other

For peer review only - http://bmjopen.bmj.com/site/about/guidelines.xhtml

	diagnoses in patients found to have unobstructed coronary arteries on emergency angiography.<br>a. Unlike echocardiography, CMR can provide a definitive ischaemic diagnosis (e.g. myocardial infarction with spontaneous reperfusion or distal embolization) or a non-ischaemic diagnosis (e.g. myocarditis, Takotsubo cardiomyopathy, aortic dissection) in patients with unobstructed coronary arteries on angiography.<br>b. A definitive diagnosis results in a patient treatment plan appropriate for that diagnosis.<br>c. A treatment plan appropriate for the diagnosis is expected to lead to a reduced risk of MACE in the long term.
6	The following statements relate to the ability of CMR to identify patients at high risk of sudden cardiac death after PPCI who would benefit most from an implantable cardiac device.<br>a. CMR identifies PPCI patients who are at high risk of sudden cardiac death better than echocardiography.<br>b. Better identification of PPCI patients at high risk of sudden cardiac death allows optimal patient selection for an implantable cardiac device (ICD or CRT).<br>c. Optimal patient selection for an implantable cardiac device is expected to lead to a reduced risk of MACE in these patients in the long term.
7	The following statements relate to the ability of CMR to identify patients who would not benefit from CRT after PPCI.<br>a. CMR identifies patients who would not benefit from CRT better than echocardiography.<br>b. The ability to identify patients who would not benefit from CRT would reduce CRT use in patients who do not need it.<br>c. Reducing CRT use in patients who do not need it is expected to lead to reduced risk of MACE in these patients in the long term.
8	The following statements relate to the ability of CMR to assess ischemia and viability in patients with multi-vessel disease.<br>a. CMR assesses ischemia and viability of the myocardium better than echocardiography.<br>b. Better assessment of ischemia and viability of the myocardium optimises the revascularisation strategy for patients with multi-vessel disease and avoids additional diagnostic tests.<br>c. The ability to optimise the revascularisation strategy for patients with multi-vessel disease is expected to lead to a reduced risk of MACE in the long term.
9	The following statements relate to the ability of CMR to identify patients with post-infarct LV thrombus.<br>a. CMR identifies post-infarct LV thrombus better than transthoracic echocardiography.<br>b. Better detection of post-infarct LV thrombus in PPCI patients allows more affected patients to be treated with anticoagulation therapy.<br>c. Treatment with anticoagulation therapy in patients with post-infarct LV thrombus is expected to lead to a reduced risk of MACE in the long term.
10	The following statements relate to the ability of CMR to detect incidental cardiac and non-cardiac findings if offered routinely to patients who undergo an emergency angiogram.<br>a. CMR identifies more incidental cardiac / non-cardiac findings than echocardiography.<br>b. Improved detection of potentially significant incidental findings allows affected patients to be investigated further and / or treated.<br>c. Further investigation and treatment is expected to reduce the risk of MACE / increase overall survival in affected patients in the long term.

For peer review only

Modified survey

Fifty four cardiologists (including five of the cardiologists who attended the formal consensus meeting) completed the survey. There was consensus that five of the 10 statements (50%) described clinically important changes in management (**Figure 1**). These were statements 3,

For peer review only - http://bmjopen.bmj.com/site/about/guidelines.xhtml

5 and 9 (which were in consensus in the first survey) and two additional statements: statement 1, relating to the ability of CMR to identify patients who have a poor prognosis after PPCI; and statement 8, relating to the ability of CMR to assess ischemia and viability in patients with MVD. There was no consensus that any of the other statements described a change in management that was not clinically important. Examples of comments relating to these five statements from cardiologists who attended the formal consensus meeting are shown in Table 2.

For peer review only

Table 1 Examples of respondents' comments for statements describing changes in management for the three statements (3, 5 & 9) that were in consensus in both the first survey and the modified survey and the two statements (1 and 8) that were in consensus in the modified survey only.

Statement 3<br><br>Patients with out-of-hospital-cardiac arrest (OHCA)	**Importance of management change**<br><br>*"CMR differentiates causes of cardiomyopathy (IHD vs not). We use it regularly in patients post out-of-hospital cardiac arrest."*<br><br>*"I have seen many cases of out-of-hospital cardiac arrest in which the use of CMR has made a diagnosis or significantly altered the diagnosis. Diagnostic refinement in the light of CMR findings also frequently results in changes in drug or device treatment, and often highlights the need for family screening. The improved anatomical, morphological, and tissue characterisation that CMR allows drives the benefit of CMR over echo and allows better identification of the cause of out-of-hospital arrest."*<br><br>**Quality of supporting evidence**<br><br>*"I don't think it has been proved that clinical outcomes are better with a CMR strategy."*<br><br>*"CMR is unlikely to alter the immediate management of these patients. However if the cause is unclear after echo and enzymes then CMR is helpful but I am not aware of any studies specifically assessing this."*
Statement 5<br><br>Patients with unobstructed arteries on angiography	**Importance of management change**<br><br>*"Agree. Unobstructed coronaries with elevated troponin need diagnostic resolution and CMR is helpful."*<br><br>*"These patients are difficult to manage and often given incorrect diagnosis and especially different theories by different doctors during same admission."*<br><br>*"I have direct experience of the benefit of CMR in this area. Therefore, I strongly agree with statement 5. CMR with LGE is especially useful and I have seen many instances when CMR after acute MI with "normal" coronary arteries has helped diagnosis, differentiating between distal vessel occlusion, LV clot, myocarditis and Takotsubo cardiomyopathy. The results of CMR in this patient group have also directly affected my management of this group of patients including drugs used and length of stay."*<br><br>*"MRI is clinically useful in this situation in my experience and occasionally makes a very useful change to management."*

For peer review only - http://bmjopen.bmj.com/site/about/guidelines.xhtml

	**Quality of supporting evidence**<br>*"Unfortunately again no RCT to indicate benefit of CMR."*
Statement 9<br>Patients with post-infarct LV thrombus	**Importance of management change**<br>*"Thrombus is poorly assessed by echo with many inconclusive reports in my experience locally."*<br>*"I strongly agree with this statement as I have experience of many patients in which echo demonstrated no LV thrombus that was subsequently found on CMR. This personal experience correlates with the studies quoted in the text of the statement."*<br>**Quality of supporting evidence**<br>*"This statement is true but whether this leads to better patient outcomes is uncertain."*
Statement 1<br>Patients with poor prognosis after PPCI	**Importance of management change**<br>*"All patients who have suffered an acute MI and subsequently undergone PPCI should receive aggressive secondary prevention. Thus, even though CMR can help refine prognosis I do not feel that this additional information would lead to any significant changes to prescribing for secondary prevention. Enhanced confidence in CMR findings (compared to echo) and refinement of prognosis with respect to infarct size, MVO and MSI may help physicians discharge/follow up patients more appropriately."*<br>*"All patients should have aggressive secondary prevention."*<br>**Quality of supporting evidence**<br>*"Whilst I agree with the evidence presented, there is no evidence to support the assertion the CMR findings lead to better outcomes for patients as there have been no trials assessing this."*<br>*"I don't think it has been proved that CMR compared with echo leads to improved patient outcomes."*<br>*"While MVO and MSI are markers of prognosis, LV systolic function remains the most important prognostic factor, which can be assessed with echo. We need interventions based on CMR parameters which improve prognosis."*<br>**Cost of CMR in relation to perceived benefit**<br>*"Agree but not sure current restricted availability and high cost and only modest anticipated change in clinical action justifies wholesale change from echo that also predicts risk well."*
Statement 8<br>Patients with MVD	**Importance of management change**<br>*"I'd accept perfusion scanning or DSE as adequate tests for ischaemia and would really only specifically request CMR if there were additional diagnostic questions."*<br>*"Total revascularisation at one sitting with FFR guidance may render this unnecessary."*<br>*"Stress CMR in my experience is better than SPECT or stress echo. This is because the improved prognostic and diagnostic accuracy helps physicians manage, with confidence, non-significant coronary disease medically rather than invasively. There are also additional benefits of CMR, for example definition of scar for CRT implant or for VT ablation. It can be helpful to "archive" this information for latter use if the patient is going to receive a device such as an ICD that may preclude latter CMR scanning."*<br>**Quality of supporting evidence**

For peer review only - http://bmjopen.bmj.com/site/about/guidelines.xhtml

	"Evidence shows improved diagnostic accuracy compared to SPECT and DSE, but not in this specific cohort." *"The evidence for ischaemia testing in the PPCI era does not really exist. There are no studies comparing MRI in this context with other modalities and definitely no RCT comparing CMR vs another modality."*

CMR: cardiovascular magnetic resonance; DSE: dobutamine stress echocardiography; FFR: fractional flow reserve; IHD: ischaemic heart disease; LGE: late gadolinium enhancement; LV: left ventricular; MI: Myocardial infarction; MSI: myocardial salvage index; MVD: multivessel disease; MVO: microvascular obstruction; PPCI: primary percutaneous coronary intervention; RCT: randomised controlled trial; SPECT: single photon emission computed tomography.

Discussion

We identified five subgroups of ACS patients who activate the PPCI pathway for whom there was consensus that CMR changes patient management in a clinically important way: i) patients who have an OHCA (about 7% of those who activate the PPCI pathway [4]); ii) patients who have a "normal" (unobstructed) coronary angiogram (about 10% of those who activate the PPCI pathway [5 6]); iii) patients who develop LV thrombus (3% overall and 9% in patients with anterior ST-elevation myocardial infarction, STEMI[7]); iv) patients who have MVD (between 40-65% of those who undergo PPCI [8-10]); and v) patients in whom CMR markers indicate poor prognosis (up to 60% of patients after STEMI [11 12]). There was consensus about the first three subgroups in both rounds of the survey, while consensus was reached about the latter subgroups in the modified survey. These results suggest that CMR benefits a large proportion of patients who activate the PPCI pathway.

Evidence for the roles for CMR in patients who activate the PPCI pathway

Cardiologists who participated in our research agreed that CMR is superior to echocardiography in establishing a diagnosis in patients who survive an OHCA, which has implications for treatment and prognosis. Despite this view, there are few studies that have reported the role of CMR in managing these patients. An unpublished retrospective case series of 54 OHCA survivors showed that CMR diagnosed the cause (ischaemic or non-ischaemic cardiomyopathy) in 40 (74%) [13].

For peer review only - http://bmjopen.bmj.com/site/about/guidelines.xhtml

The cardiologists also agreed that CMR can provide a definitive diagnosis (acute MI, acute myocarditis and cardiomyopathy, especially Takotsubo cardiomyopathy) in patients with ACS and unobstructed coronary arteries. Evidence for the role of CMR in patients with ACS and unobstructed coronary arteries also comes from small retrospective case series. These suggest that CMR provides a definitive diagnosis in 80-90% of these patients [14-16]. Without access to CMR, the management of these patients is variable in clinical practice, which may have long-term implications. A recent systematic review showed that the overall all-cause mortality in patients presenting with suspected MI and unobstructed coronary arteries was about 5% at 12 months [17].

The cardiologists agreed that CMR identifies LV thrombus better than echocardiography, which allows more patients to be identified and treated appropriately. Accurate identification of a LV thrombus is important because it often directs subsequent anticoagulation therapy to prevent embolic events. A recent systematic review, published after we conducted the formal consensus study, showed that late gadolinium enhancement CMR is the most accurate modality for detecting LV thrombus, with 88% sensitivity and 99% specificity (compared with routine echocardiography, which had 24%-33% sensitivity and 94%-95% specificity, and contrast echocardiography, which had 23%-61% sensitivity and 96%-99% specificity) [18], although most of the included studies in this review did not use a pathological or surgical gold standard for the detection of LV thrombus.

There was agreement among cardiologists that a CMR-based testing of ischaemia after PPCI would likely optimise the revascularisation strategy for these patients, although they acknowledged that there is no evidence to support the view that a CMR-based revascularisation strategy would improve outcomes for patients with MVD (see **Error! Reference source not found.**). Patients with MVD have a two-fold increase in MACE compared with patients with single-vessel disease [19]. There is continuing debate about the benefits of complete vs single lesion revascularisation during the index PPCI admission [20]. Despite recent evidence from randomised controlled trials showing improved clinical outcomes when complete revascularisation is undertaken at PPCI [21], current American and

For peer review only - http://bmjopen.bmj.com/site/about/guidelines.xhtml

European revascularization guidelines for acute MI recommend revascularisation only of the infarct-related artery at PPCI in patients with MVD [22 23].

There was considerable debate about using CMR to identify patients with poor prognosis after PPCI. Whilst there was agreement that CMR parameters of cardiac function (e.g. impaired LV function, large infarct size, microvascular obstruction, etc.) are useful for risk stratification after STEMI, there was disagreement about whether this would lead to a management change. Some cardiologists felt that there would be no changes to prescribing for secondary prevention since all patients should receive aggressive secondary prevention according to guidelines (see Error! Reference source not found.). It was acknowledged, however, that CMR markers are prognostic for outcome (statement 1a in Table 1); two recent meta analyses of prognostic studies (conducted after this formal consensus study) showed an increased risk of MACE, by 13-15% for every 10% decrease in ejection fraction assessed by CMR [11], and in patients with microvascular obstruction (MVO) assessed by CMR (odds ratio (OR) 2.60, 95% confidence interval (CI) 1.68-4.02 and OR 4.30, 95% CI 2.19-8.43, depending on method of MVO assessment) [24].

Strengths and limitations

A key strength of this study was the use of a formal consensus approach, based on a systematic search of the literature as well as expert opinion. The nominal group technique is one of the four well-established methodologies for formal consensus (the other three are the Delphi method, RAND/UCLA Appropriateness Method (RAM), and National Institutes of Health (NIH) consensus development conference methodology) [2 25]. Formal consensus is particularly useful to clarify and standardise practice when relevant and rigorous evidence is lacking. The evidence supporting many of our statements came from small retrospective case series. There were no prospective studies in the literature that reported the impact of CMR on clinical management in our population or whether any changes in management impacted on patient outcomes.

For peer review only - http://bmjopen.bmj.com/site/about/guidelines.xhtml

The main features of a formal consensus method are anonymity (statements were rated free from peer-group pressure) and iterative feedback (participants could adjust their initial rating based on the feedback of the group rating). We described the supporting evidence for each statement, identified via the literature review and fed back qualitative (panel members' comments) and quantitative (group median ratings of each statement) information at the face-to-face meeting. We also included a varied group of cardiologists from different specialties in order to encompass diverse perspectives

The number of panel members in our study was lower than the recommended 8-12 members for a consensus panel [25]. Nevertheless, smaller groups are preferable to larger ones since, although having more group members increases the reliability of group judgement, large groups reduce the ability to elicit potentially important contributions from every member of the panel [25]. To overcome any criticism of the number of panel members in our study and to prevent the possibility of introducing bias (given the self-selected nature of the panel), we extended the survey to UK cardiologists who did not participate in the formal consensus process. Apart from one statement (statement 1, relating to the ability of CMR to identify patients with poor prognosis after PPCI), the same statements were in consensus when the survey was completed by cardiologists external to the consensus process. This indicates that our process was robust and our survey statements were clear and unambiguous.

A further limitation of our study was that we did not include other stakeholders (e.g. general practitioners and patient representatives) in the consensus process.[26] Although we had originally planned to do so, we decided that the specialised nature of the statements would not be easily understood by those without cardiology expertise.

Conclusions and future research

We have identified the main ways in which cardiologists believe CMR changes clinical management in five subgroups of patients who activate the PPCI pathway. These clinically important changes in management can now be used to design a composite primary

For peer review only - http://bmjopen.bmj.com/site/about/guidelines.xhtml

outcome for an evaluation of the effectiveness and cost-effectiveness of CMR. It will allow evidence to be obtained more quickly to inform decisions about implementing CMR than would be possible for an evaluation based on MACE.

Authors' contributions

MP: determined the structure of the consensus process, conducted the literature review, formulated statements based on the literature review, was a member of the working group and wrote the manuscript.

RB: contributed to the consensus process, was a member of the working group, co-wrote the manuscript and approved the final manuscript.

CBD: conceived the overall feasibility study, contributed to the consensus process, was a member of the working group, revised the manuscript with respect to intellectual content and approved the final manuscript.

JPG: contributed to the consensus process, was a member of the working group, revised the manuscript with respect to intellectual content and approved the final manuscript.

SD: contributed to the consensus process, was a member of the working group, revised the manuscript with respect to intellectual content and approved the final manuscript.

RA: contributed to the consensus process, was a member of the working group, revised the manuscript with respect to intellectual content and approved the final manuscript.

JH: reviewed the statistical analysis, was a member of the working group, revised the manuscript with respect to intellectual content and approved the final manuscript

EM: contributed to the formulation of the consensus statements, revised the manuscript with respect to intellectual content and approved the final manuscript

CR: reviewed the analysis, was a member of the working group and approved the final manuscript

BR: chief investigator who conceived the overall feasibility study, including the consensus element, was a member of the working group, revised the manuscript with respect to intellectual content and approved the final manuscript.

For peer review only - http://bmjopen.bmj.com/site/about/guidelines.xhtml

Funding

This study is part of a project funded by the National Institute of Health Research (NIHR) Health Services & Delivery Research (HS&DR 11/2003/58). The British Heart Foundation and NIHR Bristol Biomedical Research Unit for Cardiovascular Disease funded some staff time (CAR, CBD, JH, EM, BCR). The views and opinions expressed are those of the authors and do not necessarily reflect those of the HS&DR programme, NIHR, the UK NHS or the Department of Health.

Competing interests

JPG has received a research grant from Philips Healthcare. The remaining authors declare that they have no competing interests.

Data sharing statement

The literature search based on the published search strategy, details of development of the consensus statements and the survey results can be obtained by contacting the corresponding author (maria.pufulete@bristol.ac.uk).

Acknowledgements

This project was funded by the National Institute for Health Research HS & DR Programme (project number 11/2003/58). The views and opinions expressed therein are those of the authors and do not necessarily reflect those of the HS & DR Programme, NIHR, NHS or the Department of Health.

References

1. Antony R, Daghem M, McCann GP, et al. Cardiovascular magnetic resonance activity in the United Kingdom: a survey on behalf of the British Society of Cardiovascular Magnetic Resonance. *J Cardiovasc Magn Reson* 2011;13:57. doi: 10.1186/1532-429x-13-57 [published Online First: 2011/10/08]
2. Nair R, Aggarwal R, Khanna D. Methods of formal consensus in classification/diagnostic criteria and guideline development. *Seminars in arthritis and rheumatism* 2011;41(2):95-105. doi: 10.1016/j.semarthrit.2010.12.001 [published Online First: 2011/03/23]

For peer review only - http://bmjopen.bmj.com/site/about/guidelines.xhtml

3. Rycroft-Malone J. Formal consensus: the development of a national clinical guideline. *Qual Health Care* 2001;10(4):238-44. [published Online First: 2001/12/18]
4. Choudry FA, Weerackody RP, Timmis AD, et al. Importance of primary percutaneous coronary intervention for reducing mortality in ST-elevation myocardial infarction complicated by out of hospital cardiac arrest. *European heart journal Acute cardiovascular care* 2015;4(4):378-85. doi: 10.1177/2048872614555990 [published Online First: 2014/10/19]
5. Larsen AI, Galbraith PD, Ghali WA, et al. Characteristics and outcomes of patients with acute myocardial infarction and angiographically normal coronary arteries. *Am J Cardiol* 2005;95(2):261-3. doi: S0002-9149(04)01535-8 [pii]

10.1016/j.amjcard.2004.09.014 [doi] [published Online First: 2005/01/12]
6. Meryon I, Patel N, Millane T, et al. Normal coronary angiography and primary percutaneous coronary intervention for ST elevation myocardial infarction: a literature review and audit findings. *Int J Clin Pract* 2010;64(9):1245-51. doi: IJCP2394 [pii]

10.1111/j.1742-1241.2010.02394.x [doi] [published Online First: 2010/07/27]
7. Robinson AA, Jain A, Gentry M, et al. Left ventricular thrombi after STEMI in the primary PCI era: A systematic review and meta-analysis. *Int J Cardiol* 2016;221:554-59. doi: 10.1016/j.ijcard.2016.07.069 [published Online First: 2016/07/18]
8. Corpus RA, House JA, Marso SP, et al. Multivessel percutaneous coronary intervention in patients with multivessel disease and acute myocardial infarction. *Am Heart J* 2004;148(3):493-500. doi: 10.1016/j.ahj.2004.03.051 [published Online First: 2004/09/25]
9. Goldstein JA, Demetriou D, Grines CL, et al. Multiple complex coronary plaques in patients with acute myocardial infarction. *N Engl J Med* 2000;343(13):915-22. doi: 10.1056/NEJM200009283431303 [doi] [published Online First: 2000/09/28]
10. Toma M, Buller CE, Westerhout CM, et al. Non-culprit coronary artery percutaneous coronary intervention during acute ST-segment elevation myocardial infarction: insights from the APEX-AMI trial. *Eur Heart J* 2010;31(14):1701-7. doi: 10.1093/eurheartj/ehq129 [published Online First: 2010/06/10]
11. El Aidi H, Adams A, Moons KG, et al. Cardiac magnetic resonance imaging findings and the risk of cardiovascular events in patients with recent myocardial infarction or suspected or known coronary artery disease: a systematic review of prognostic studies. *J Am Coll Cardiol* 2014;63(11):1031-45. doi: 10.1016/j.jacc.2013.11.048 [published Online First: 2014/02/04]
12. van Kranenburg M, Magro M, Thiele H, et al. Prognostic value of microvascular obstruction and infarct size, as measured by CMR in STEMI patients. *JACC Cardiovasc Imaging* 2014;7(9):930-9. doi: 10.1016/j.jcmg.2014.05.010 [published Online First: 2014/09/13]
13. Dastidar AG, Ahmed A, McAlindon E, et al. Incremental Value of Cardiovascular Magnetic Resonance Imaging in Patients Surviving non Traumatic out of Hospital Cardiac Arrest: A Tertiary UK Centre Experience. *Heart (British Cardiac Society)* 2014;100:A79-A80.
14. Assomull RG, Lyne JC, Keenan N, et al. The role of cardiovascular magnetic resonance in patients presenting with chest pain, raised troponin, and unobstructed coronary arteries. *Eur Heart J* 2007;28(10):1242-9. doi: ehm113 [pii]

10.1093/eurheartj/ehm113 [doi] [published Online First: 2007/05/05]
15. Leurent G, Langella B, Fougerou C, et al. Diagnostic contributions of cardiac magnetic resonance imaging in patients presenting with elevated troponin, acute chest pain syndrome and unobstructed coronary arteries. *Arch Cardiovasc Dis* 2011;104(3):161-70. doi: S1875-2136(11)00055-6 [pii]

10.1016/j.acvd.2011.01.005 [doi] [published Online First: 2011/04/19]

For peer review only - http://bmjopen.bmj.com/site/about/guidelines.xhtml

For peer review only

16. Monney PA, Sekhri N, Burchell T, et al. Acute myocarditis presenting as acute coronary syndrome: role of early cardiac magnetic resonance in its diagnosis. *Heart (British Cardiac Society)* 2011;97(16):1312-8. doi: 10.1136/hrt.2010.204818 [published Online First: 2010/11/26]
17. Pasupathy S, Air T, Dreyer RP, et al. Systematic review of patients presenting with suspected myocardial infarction and nonobstructive coronary arteries. *Circulation* 2015;131(10):861-70. doi: 10.1161/circulationaha.114.011201 [published Online First: 2015/01/15]
18. Roifman I, Connelly KA, Wright GA, et al. Echocardiography vs. Cardiac Magnetic Resonance Imaging for the Diagnosis of Left Ventricular Thrombus: A Systematic Review. *The Canadian journal of cardiology* 2015;31(6):785-91. doi: 10.1016/j.cjca.2015.01.011 [published Online First: 2015/04/29]
19. Sorajja P, Gersh BJ, Cox DA, et al. Impact of multivessel disease on reperfusion success and clinical outcomes in patients undergoing primary percutaneous coronary intervention for acute myocardial infarction. *Eur Heart J* 2007;28(14):1709-16. doi: 10.1093/eurheartj/ehm184 [published Online First: 2007/06/09]
20. Ruggieri A, Piraino D, Dendramis G, et al. STEMI patients and nonculprit lesions: To treat or not to treat? and when? A review of most recent literature. *Catheter Cardiovasc Interv* 2015 doi: 10.1002/ccd.26236 [published Online First: 2015/09/15]
21. El-Hayek GE, Gershlick AH, Hong MK, et al. Meta-Analysis of Randomized Controlled Trials Comparing Multivessel Versus Culprit-Only Revascularization for Patients With ST-Segment Elevation Myocardial Infarction and Multivessel Disease Undergoing Primary Percutaneous Coronary Intervention. *Am J Cardiol* 2015;115(11):1481-6. doi: 10.1016/j.amjcard.2015.02.046 [published Online First: 2015/04/05]
22. O'Gara PT, Kushner FG, Ascheim DD, et al. 2013 ACCF/AHA guideline for the management of ST-elevation myocardial infarction: a report of the American College of Cardiology Foundation/American Heart Association Task Force on Practice Guidelines. *J Am Coll Cardiol* 2013;61(4):e78-140. doi: 10.1016/j.jacc.2012.11.019 [published Online First: 2012/12/22]
23. Steg PG, James SK, Atar D, et al. ESC Guidelines for the management of acute myocardial infarction in patients presenting with ST-segment elevation. *Eur Heart J* 2012;33(20):2569-619. doi: 10.1093/eurheartj/ehs215 [published Online First: 2012/08/28]
24. Hamirani YS, Wong A, Kramer CM, et al. Effect of microvascular obstruction and intramyocardial hemorrhage by CMR on LV remodeling and outcomes after myocardial infarction: a systematic review and meta-analysis. *JACC Cardiovasc Imaging* 2014;7(9):940-52. doi: 10.1016/j.jcmg.2014.06.012 [published Online First: 2014/09/13]
25. Murphy MK, Black NA, Lamping DL, et al. Consensus development methods, and their use in clinical guideline development. *Health technology assessment (Winchester, England)* 1998;2(3):i-iv, 1-88. [published Online First: 1998/04/30]
26. Hutchings A, Raine R. A systematic review of factors affecting the judgments produced by formal consensus development methods in health care. *Journal of health services research & policy* 2006;11(3):172-9. doi: 10.1258/135581906777641659 [published Online First: 2006/07/11]

For peer review only - http://bmjopen.bmj.com/site/about/guidelines.xhtml

Figure legend

Figure 1 Median and interquartile (IQ) range for the 10 statements in the modified survey (n=54). Boxes represent the median and IQ range and the whiskers represent the range. Dots represent extreme values. Consensus was based on the distribution of responses to a, b and c. Statements 1, 3, 5, 8 and 9 were considered to be in consensus.

For peer review only

For peer review only - http://bmjopen.bmj.com/site/about/guidelines.xhtml

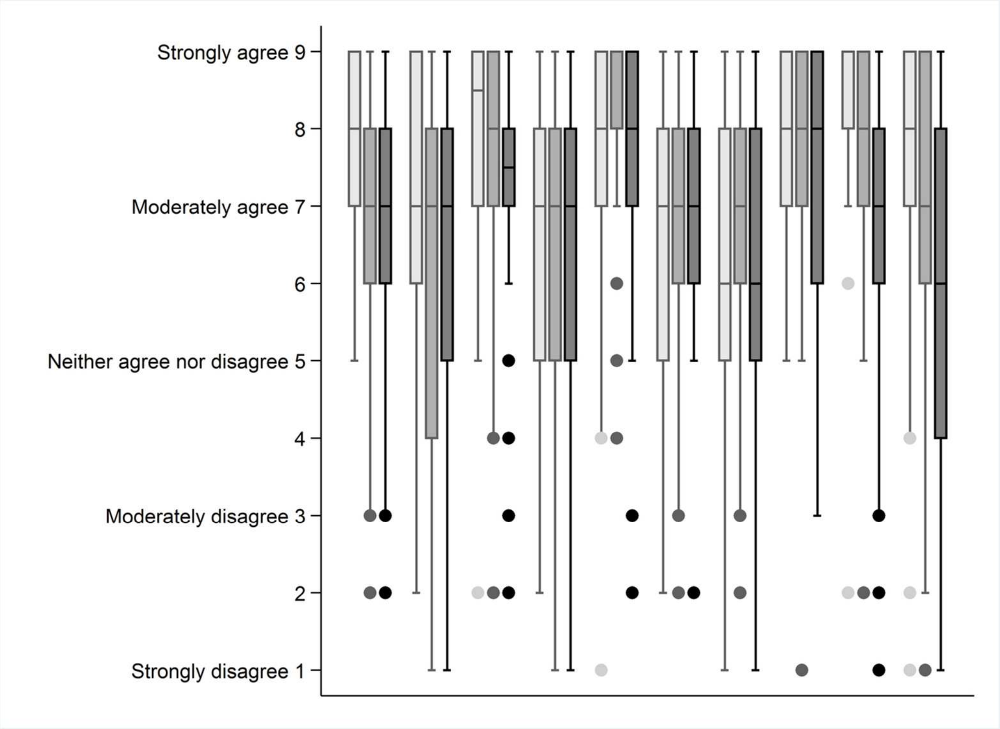

Figure 1 Median and interquartile (IQ) range for the 10 statements in the modified survey (n=54). Boxes represent the median and IQ range and the whiskers represent the range. Dots represent extreme values. Consensus was based on the distribution of responses to a, b and c. Statements 1, 3, 5, 8 and 9 were considered to be in consensus.

126x91mm (300 x 300 DPI)

Appendix 1

Literature search

Medline (1950 to Jan 2014)

1 exp Myocardial Infarction/ (142973)
2 myocardial infarct$.tw. (134547)
3 heart attack$.tw. (3601)
4 MI.tw. (25881)
5 ami.tw. (12166)
6 stemi.tw. (3663)
7 heart infarct$.tw. (705)
8 Acute Coronary Syndrome/ (6784)
9 acute coronary syndrome$.tw. (15521)
10 ACS.tw. (9320)
11 or/1-10 (210783)
12 exp angioplasty/ (53568)
13 angioplasty.tw. (34003)
14 percutaneous coronary intervention$.tw. (14406)
15 exp Percutaneous Coronary Intervention/ (34668)
16 PCI.tw. (11157)
17 PPCI.tw. (366)
18 exp Stents/ (50784)
19 stent$.tw. (56427)
20 Myocardial Revascularization/ (9079)
21 revasculari$.tw. (37853)
22 reperfused.tw. (5688)
23 reperfusion.tw. (55775)
24 or/12-23 (191056)
25 exp Magnetic Resonance Imaging/ (295774)
26 Magnetic resonance imag$.tw. (122420)
27 Cardiovascular magnetic resonance.tw. (1765)
28 cardiac magnetic resonance.tw. (3179)
29 MRI.tw. (121901)
30 CMR.tw. (3035)
31 or/25-30 (344849)
32 24 and 31 (5713)
33 11 and 32 (1318)
34 exp animals/ not humans/ (3863199)
35 33 not 34 (1051)

Embase (1980 to Jan 2014)

1 exp heart infarction/ (261299)
2 myocardial infarct$.tw. (185732)
3 heart attack$.tw. (4991)
4 MI.tw. (45694)
5 ami.tw. (19697)
6 stemi.tw. (10463)
7 heart infarct$.tw. (1450)
8 exp Acute Coronary Syndrome/ (26244)
9 acute coronary syndrome$.tw. (28004)
10 ACS.tw. (17708)
11 or/1-10 (337561)

For peer review only

12 exp angioplasty/ (67762)
13 angioplasty.tw. (47179)
14 percutaneous coronary intervention$.tw. (27203)
15 exp Percutaneous Coronary Intervention/ (59160)
16 PCI.tw. (26664)
17 PPCI.tw. (1281)
18 Stent/ (64591)
19 exp cardiovascular stent/ (34380)
20 stent$.tw. (94492)
21 heart muscle revascularization/ (21848)
22 revasculari$.tw. (56445)
23 reperfused.tw. (7304)
24 reperfusion.tw. (77453)
25 or/12-24 (298341)
26 exp nuclear magnetic resonance imaging/ (524106)
27 Magnetic resonance imag$.tw. (164672)
28 Cardiovascular magnetic resonance.tw. (3414)
29 cardiac magnetic resonance.tw. (6341)
30 MRI.tw. (203793)
31 CMR.tw. (7673)
32 or/26-31 (563445)
33 25 and 32 (13408)
34 11 and 33 (3451)
35 (exp animal/ or nonhuman/) not exp humans/ (5173875)
36 34 not 35 (3048)
37 limit 36 to embase (2864)

The Cochrane Library

#1 MeSH descriptor: [Myocardial Infarction] explode all trees
#2 MeSH descriptor: [Acute Coronary Syndrome] explode all trees
#3 myocardial next infarct*
#4 heart next attack*
#5 MI
#6 AMI
#7 STEMI
#8 heart next infarct*
#9 "acute coronary syndrome*"
#10 #1 or #2 or #3 or #4 or #5 or #6 or #7 or #8 or #9
#11 MeSH descriptor: [Angioplasty] explode all trees
#12 MeSH descriptor: [Percutaneous Coronary Intervention] explode all trees
#13 MeSH descriptor: [Stents] explode all trees
#14 MeSH descriptor: [Myocardial Revascularization] this term only
#15 angioplasty
#16 "percutaneous coronary intervention*"
#17 PCI or PPCI
#18 stent*
#19 revasculari*
#20 reperfused
#21 reperfusion
#22 #11 or #12 or #13 or #14 or #15 or #16 or #17 or #18 or #19 or #20 or #21
#23 MeSH descriptor: [Magnetic Resonance Imaging] explode all trees
#24 "Magnetic resonance imag*"
#25 " Cardiovascular magnetic resonance"
#26 "cardiac magnetic resonance"

For peer review only

#27 MRI or CMR
#28 #23 or #24 or #25 or #26 or #27
#29 #11 and #22 and #28

ISI Web of Science

4 1,351 #3 AND #2 AND #1
3 218,389 TOPIC: (Angioplasty or "percutaneous coronary intervention*" or PCI or PPCI or stent* or revasulari* or reperfused or reperfusion)
2 257,465 TOPIC: ("Magnetic Resonance Imag*" or "Cardiovascular magnetic resonance" or " cardiac magnetic resonance" or MRI or CMR)
1 273,481 TOPIC: ("myocardial infarct*" or "heart attack*" or "heart infarct*" or "acute coronary syndrome*" or MI or AMI or STEMI or ACS)

BIOSIS

6 873 #5 AND #4
5 TAXONOMIC DATA: (human not (animal* not human))
4 #3 AND #2 AND #1
3 TOPIC: (Angioplasty or "percutaneous coronary intervention*" or PCI or PPCI or stent* or revasulari* or reperfused or reperfusion)
2 TOPIC: ("Magnetic Resonance Imag*" or "Cardiovascular magnetic resonance" or " cardiac magnetic resonance" or MRI or CMR)
1 TOPIC: ("myocardial infarct*" or "heart attack*" or "heart infarct*" or "acute coronary syndrome*" or MI or AMI or STEMI or ACS)

For peer review only

Appendix 2

First survey (statements, supporting paragraphs and references)

Benefit to patients and the NHS of cardiac magnetic resonance imaging (CMR) after Primary percutaneous coronary Intervention (PPCI) Pathway Activation

Thank you for agreeing to complete this survey. We expect the survey to take about 20 minutes of your time. You are asked to read and rate 12 statements relating to the potential impact of cardiac magnetic resonance imaging (CMR) in changing the management of patients in a clinically important way. The patient population under consideration includes all patients who activate the primary percutaneous coronary intervention (PPCI) pathway. Please rate your agreement with each statement on the scale provided. Each statement has a supporting paragraph that provides background information and links to full text references. We believe we have used the best available evidence but we are aware that the evidence is of variable quality.

In order to progress through the survey, please use the following navigation buttons:

Click the Next button to go to the next page.

Click the Previous button to return to the previous page.

Click the Number buttons in the text to access the full references.

Click the Submit button to submit the survey at the end.

Statement 1. *Compared with echocardiography, CMR after PPCI allows patients with CMR markers that indicate **poor** prognosis (e.g. impaired left ventricular function, large infarct size, microvascular obstruction) to be followed up more appropriately and undergo more aggressive medical therapy for secondary prevention.*

Accurate assessment of infarct characteristics is important for risk stratification after PPCI. CMR can quantify in a single scan all cardiac markers relevant to PPCI outcomes, with high reproducibility and accuracy. CMR has high spatial and temporal resolution and is superior to echocardiography for measuring left ventricular volumes and ejection fraction. **[1]** Additionally, CMR markers such as infarct size, microvascular obstruction and myocardial salvage have been shown to have long term prognostic value. **[2-4]** Late gadolinium enhancement CMR has added prognostic value over echocardiography. **[5]**

Statement 2. *Compared with echocardiography, CMR after PPCI allows patients with CMR markers that indicate **good** prognosis (e.g. normal left ventricular function, high myocardial salvage, no microvascular obstruction, no residual ischemia) to be discharged earlier and followed up less frequently.*

For peer review only - http://bmjopen.bmj.com/site/about/guidelines.xhtml

CMR measures several markers (infarct size, myocardial salvage index, microvascular obstruction, myocardial oedema and haemorrhage) that cannot be measured by echocardiography. These markers can be used to predict cardiac remodelling and prognosis after myocardial infarction, **[2, 4]** determine the optimal therapeutic pathway for each patient and prevent over- and under-treatment.

Statement 3. *Compared with echocardiography, CMR better identifies the cause of out of hospital cardiac arrest [e.g. large myocardial infarction, arrhythmogenic right ventricular cardiomyopathy (ARVC), aberrant coronary arteries, hypertrophic cardiomyopathy (HCM)] to optimise further treatment for the patient (e.g. defibrillator for primary arrhythmia or percutaneous coronary intervention) or family members.*

Out-of-hospital cardiac arrest affects about 60,000 people in the UK each year. Currently, hospital survival is about 32%. Early identification of the cause is essential to improve survival. Causes of out-of-hospital cardiac arrest include myocardial infarction (40%-90%) and inherited cardiomyopathies such as ARVC and HCM. Unlike echocardiography, CMR allows in vivo tissue characterisation, which differentiates scarring resulting from myocardial infarction from other causes of focal fibrosis (e.g. observed in non-ischaemic cardiomyopathies such as ARVC and HCM). **[6]** Late gadolinium enhancement on CMR can predict serious cardiac complications in patients with HCM (all-cause death, cardiac death and death from heart failure), **[7]** and identifies patients that may need more aggressive medical and device therapy (e.g. renin-aldosterone system inhibition for prevention of heart failure or implantable cardioverter defibrillator placement for primary prevention of sudden cardiac death).

Statement 4. *CMR after PPCI identifies patients at high risk of having ventricular septal defect (VSD) or impending cardiac rupture, who may require ventricular patch or other urgent cardiac surgery, and guides the optimal management of these patients.*

VSD and left ventricular free wall rupture are rare complications of myocardial infarction, occurring in less than 1% of PPCI patients, usually within one week of the infarct. Mortality ranges between 50% and 80%. Diagnosis can be difficult to make and usually requires multi-modality imaging (echocardiography, ventriculography, computed tomography, CMR) before surgical repair. Because of its high spatial resolution, CMR can be used to clarify the detailed structure of these lesions. CMR accurately identifies the location, size and tissue margins of VSD and is useful for detecting apical defects, which are not easily identified by echocardiography. CMR measurements may also be used to determine the size of ventricular patch required to close the VSD, which avoids the inflation of a sizing balloon in friable infarcted tissue. **[8]** These features, together with other CMR markers of damage (e.g. infarct size, microvascular obstruction, left ventricular dysfunction) are useful for guiding optimal management of patients with post-infarct VSD. Similarly, CMR can differentiate between the different types of impending free wall rupture (acute, sub-acute and chronic), which may be helpful in planning surgical management. **[9]**

Statement 5. *In patients with a "normal" (unobstructed) coronary angiogram, CMR can differentiate patients who have had a myocardial infarction with spontaneous reperfusion or distal embolization from patients with a non-ischaemic diagnosis (e.g. myocarditis, Takotsubo cardiomyopathy, aortic dissection), resulting in a patient treatment plan appropriate for the definitive diagnosis.*

The incidence of unobstructed coronary angiogram in patients who activate the PPCI pathway is between 5% and 12%. In these patients, the lack of an accurate diagnosis may result in inappropriate or unnecessary treatment and/or follow-up and poorer prognosis. **[10]**

For peer review only - http://bmjopen.bmj.com/site/about/guidelines.xhtml

CMR facilitates differential diagnosis in the context of an unobstructed coronary angiogram, providing a definitive diagnosis (e.g. myocardial infarction, myocarditis, Takotsubo cardiomyopathy) in 65-90% of these patients. **[11, 12]** In patients with myocarditis, late gadolinium enhancement (LGE) on CMR may predict long term adverse outcomes. **[13]** In the context of myocardial infarction without an angiographic lesion, CMR can locate the culprit infarct-related artery in patients with spontaneous reperfusion or with distal embolization.

Statement 6. *Compared with echocardiography, CMR after PPCI better identifies patients at high risk of sudden cardiac death who* ***would*** *benefit most from an implantable cardiac device [e.g.* implantable cardioverter defibrillator (*ICD), cardiac resynchronisation therapy, (CRT)].*

About 6% of heart attack patients subsequently die suddenly from a presumed cardiac cause. Current guidelines recommend the use of ICD to prevent sudden cardiac death in patients who have a low left ventricular ejection fraction (EF) after a heart attack. EF is used in clinical practice to make decisions about ICD implantation, but it has a low predictive value and many patients with an ICD will never benefit from it. **[14]** EF is most commonly measured by echocardiography, but CMR is now considered the gold standard for EF measurement because it is more reproducible than echocardiography. **[1]** CMR has been shown to be better than echocardiography for selecting patients for ICD implantation when strict EF thresholds are used to guide implantation. **[15]** The extent of myocardial scar characterised by late gadolinium enhancement (LGE) CMR may also be used to predict whether ICD implantation is appropriate in this patient group. **[16]** Furthermore, LGE CMR can guide placement of the left ventricular lead away from scarred myocardium, which results in a better clinical outcome after CRT. **[17]**

Statement 7. *Compared with echocardiography, CMR after PPCI better identifies patients who* ***would not*** *benefit from cardiac resynchronisation therapy (CRT).*

CRT (or biventricular pacing) uses a specialised pacemaker to resynchronise the beating of the two ventricles by pacing both simultaneously, which improves the contraction of the left ventricle and the overall efficiency of the heart. It is used in patients with systolic ventricular dysfunction and heart failure. However, about 30% of patients who meet the inclusion criteria for CRT do not respond to it. **[18]** CMR assessment of mechanical dyssynchrony and myocardial scar provides additional value over echocardiography for identifying responsive patients. **[19, 20]** Myocardial scar is an important feature of non-response to CRT **[21, 22]** and late gadolinium enhancement (LGE) CMR accurately differentiates between transmural, mid-myocardial, epicardial, and subendocardial scar.

Statement 8. *Compared with stress echocardiography* or *single photon emission computed tomography (SPECT), CMR after PPCI in patients with multi-vessel disease better assesses ischaemia and viability of the myocardium to optimise the revascularisation strategy for the patient and avoid additional diagnostic tests.*

Between 40% and 65% of the patients who activate the PPCI pathway have multi-vessel disease. Adequate assessment of residual ischemia in non-culprit arteries post PPCI is important for effective management because this patient group has an adverse prognosis. Various techniques are currently used to assess the need for additional revascularisation, including stress echocardiography, SPECT and stress-perfusion CMR. Stress CMR has excellent prognostic value **[23]** and better diagnostic accuracy than SPECT **[24, 25]** or stress echocardiography **[26]** for detecting angiographically significant coronary artery disease.

For peer review only - http://bmjopen.bmj.com/site/about/guidelines.xhtml

For peer review only

Statement 9. *Compared with echocardiography, CMR after PPCI better identifies patients with left ventricular (LV) thrombus for treatment with anticoagulation therapy.*

LV thrombus is a serious complication of acute myocardial infarction. It increases risk of thromboembolic events, particularly stroke. LV thrombus develops in up to 10% of patients with anterior wall infarctions after PPCI. Although echocardiography is most commonly used to detect LV thrombus and to assess its shape and size, between 10-46% of echocardiograms are inconclusive. **[27]** Late gadolinium enhancement (LGE) CMR is considered the gold standard for detecting LV thrombus, because it detects thrombus based on tissue characteristics rather than anatomic appearance. Fewer thrombi are detected by contrast-echocardiography than by LGE CMR. **[28]** CMR has a higher sensitivity (88%) than contrast (61%) and noncontrast (<33%) echocardiography. **[28, 29]**

Statement 10. *Compared with other imaging modalities, in patients with multi-vessel disease, CMR after PPCI better identifies the artery that caused the myocardial infarction and guides the subsequent treatment plan in relation to additional revascularisation (percutaneous coronary intervention, or coronary artery bypass graft surgery).*

In patients with multi vessel disease presenting with acute MI, the culprit artery is not always easy to identify as more than one artery could be responsible. CMR has higher spatial resolution than single photon emission computed tomography (SPECT), allowing easy identification of the infarct-related artery and non-culprit territory stenosis in PPCI patients, **[30]** and hence appropriate risk-stratification of patients with multi-vessel disease for planning their future treatment. **[31]**

Statement 11. *Compared with echocardiography, CMR after PPCI better identifies incidental cardiac findings that may need further investigation and treatment.*

Compared with echocardiography, CMR provides versatile imaging planes, superior tissue contrast and advanced tissue characterisation, allowing a comprehensive assessment of cardiac anatomy, function and flow, and imaging of the great vessels (including venous return), the pericardium and suspected cardiac tumours. **[32]** As such, CMR can identify congenital coronary anomalies, cardiac masses, coronary artery aneurysms, valvular heart disease, thoracic aortic disease, etc. CMR can adequately differentiate benign from malignant tumours in the heart. **[33]**

Statement 12. *Compared with echocardiography, CMR after PPCI identifies significant incidental non-cardiac findings (e.g. oesophageal and lung tumours, pulmonary embolus, aortic aneurysm) that may need further investigation and treatment.*

Unlike echocardiography, which provides limited imaging of mediastinal and extra-cardiac structures, CMR images a substantial part of the thorax and abdomen in the field of view, which may potentially contain non-cardiac abnormalities. The prevalence of non-cardiac findings on CMR is up to 80% (with up to 30% of these representing potentially significant findings), depending on the characteristics of the population examined. **[34-36]**

References

1. Dorosz JL, Lezotte DC, Weitzenkamp DA, Allen LA, Salcedo EE: **Performance of 3-dimensional echocardiography in measuring left ventricular volumes and ejection fraction: a systematic review and meta-analysis**. *J Am Coll Cardiol* 2012, **59**(20):1799-1808.

For peer review only - http://bmjopen.bmj.com/site/about/guidelines.xhtml

2. Eitel I, Desch S, de WS, Fuernau G, Gutberlet M, Schuler G, Thiele H: **Long-term prognostic value of myocardial salvage assessed by cardiovascular magnetic resonance in acute reperfused myocardial infarction**. *Heart* 2011, **97**(24):2038-2045.
3. Eitel I, Desch S, Fuernau G, Hildebrand L, Gutberlet M, Schuler G, Thiele H: **Prognostic significance and determinants of myocardial salvage assessed by cardiovascular magnetic resonance in acute reperfused myocardial infarction**. *J Am Coll Cardiol* 2010, **55**(22):2470-2479.
4. Klug G, Mayr A, Schenk S, Esterhammer R, Schocke M, Nocker M, Jaschke W, Pachinger O, Metzler B: **Prognostic value at 5 years of microvascular obstruction after acute myocardial infarction assessed by cardiovascular magnetic resonance**. *Journal of Cardiovascular Magnetic Resonance* 2012, **14**:46.
5. Wong TC, Piehler K, Puntil KS, Moguillansky D, Meier CG, Lacomis JM, Kellman P, Cook SC, Schwartzman DS, Simon MA *et al*: **Effectiveness of late gadolinium enhancement to improve outcomes prediction in patients referred for cardiovascular magnetic resonance after echocardiography**. *Journal of cardiovascular magnetic resonance : official journal of the Society for Cardiovascular Magnetic Resonance* 2013, **15**:6.
6. Parsai C, O'Hanlon R, Prasad SK, Mohiaddin RH: **Diagnostic and prognostic value of cardiovascular magnetic resonance in non-ischaemic cardiomyopathies**. *Journal of cardiovascular magnetic resonance : official journal of the Society for Cardiovascular Magnetic Resonance* 2012, **14**:54.
7. Green JJ, Berger JS, Kramer CM, Salerno M: **Prognostic value of late gadolinium enhancement in clinical outcomes for hypertrophic cardiomyopathy**. *JACC Cardiovascular imaging* 2012, **5**(4):370-377.
8. Artis NJ, Thomson J, Plein S, Greenwood JP: **Percutaneous closure of postinfarction ventricular septal defect: cardiac magnetic resonance-guided case selection and postprocedure evaluation**. *The Canadian journal of cardiology* 2011, **27**(6):869 e863-865.
9. Galvin S, Chen V, Bunton R, Doyle T: **Sutureless pericardial patch augmentation for impending left ventricular free wall rupture**. *Interactive cardiovascular and thoracic surgery* 2010, **10**(2):341-343.
10. Dokainish H, Pillai M, Murphy SA, DiBattiste PM, Schweiger MJ, Lotfi A, Morrow DA, Cannon CP, Braunwald E, Lakkis N: **Prognostic implications of elevated troponin in patients with suspected acute coronary syndrome but no critical epicardial coronary disease: a TACTICS-TIMI-18 substudy**. *J Am Coll Cardiol* 2005, **45**(1):19-24.
11. Assomull RG, Lyne JC, Keenan N, Gulati A, Bunce NH, Davies SW, Pennell DJ, Prasad SK: **The role of cardiovascular magnetic resonance in patients presenting with chest pain, raised troponin, and unobstructed coronary arteries**. *Eur Heart J* 2007, **28**(10):1242-1249.
12. Leurent G, Langella B, Fougerou C, Lentz PA, Larralde A, Bedossa M, Boulmier D, Le Breton H: **Diagnostic contributions of cardiac magnetic resonance imaging in patients presenting with elevated troponin, acute chest pain syndrome and unobstructed coronary arteries**. *Arch Cardiovasc Dis* 2011, **104**(3):161-170.
13. Grun S, Schumm J, Greulich S, Wagner A, Schneider S, Bruder O, Kispert EM, Hill S, Ong P, Klingel K *et al*: **Long-term follow-up of biopsy-proven viral myocarditis: predictors of mortality and incomplete recovery**. *J Am Coll Cardiol* 2012, **59**(18):1604-1615.
14. Moss AJ, Greenberg H, Case RB, Zareba W, Hall WJ, Brown MW, Daubert JP, McNitt S, Andrews ML, Elkin AD *et al*: **Long-term clinical course of patients after termination of ventricular tachyarrhythmia by an implanted defibrillator**. *Circulation* 2004, **110**(25):3760-3765.
15. Joshi SB, Connelly KA, Jimenez-Juan L, Hansen M, Kirpalani A, Dorian P, Mangat I, Al-Hesayen A, Crean AM, Wright GA *et al*: **Potential clinical impact of cardiovascular magnetic resonance assessment of ejection fraction on eligibility for cardioverter defibrillator**

For peer review only

implantation. *Journal of cardiovascular magnetic resonance : official journal of the Society for Cardiovascular Magnetic Resonance* 2012, **14**:69.

16. Alexandre J, Saloux E, Dugue AE, Lebon A, Lemaitre A, Roule V, Labombarda F, Provost N, Gomes S, Scanu P *et al*: **Scar extent evaluated by late gadolinium enhancement CMR: a powerful predictor of long term appropriate ICD therapy in patients with coronary artery disease**. *Journal of cardiovascular magnetic resonance : official journal of the Society for Cardiovascular Magnetic Resonance* 2013, **15**:12.
17. Leyva F, Foley PW, Chalil S, Ratib K, Smith RE, Prinzen F, Auricchio A: **Cardiac resynchronization therapy guided by late gadolinium-enhancement cardiovascular magnetic resonance**. *Journal of cardiovascular magnetic resonance : official journal of the Society for Cardiovascular Magnetic Resonance* 2011, **13**:29.
18. Leclercq C, Kass DA: **Retiming the failing heart: principles and current clinical status of cardiac resynchronization**. *J Am Coll Cardiol* 2002, **39**(2):194-201.
19. Bilchick KC, Dimaano V, Wu KC, Helm RH, Weiss RG, Lima JA, Berger RD, Tomaselli GF, Bluemke DA, Halperin HR *et al*: **Cardiac magnetic resonance assessment of dyssynchrony and myocardial scar predicts function class improvement following cardiac resynchronization therapy**. *JACC Cardiovascular imaging* 2008, **1**(5):561-568.
20. Ellims AH, Pfluger H, Elsik M, Butler MJ, Hare JL, Taylor AJ: **Utility of cardiac magnetic resonance imaging, echocardiography and electrocardiography for the prediction of clinical response and long-term survival following cardiac resynchronisation therapy**. *Int J Cardiovasc Imaging* 2013, **29**(6):1303-1311.
21. Adelstein EC, Saba S: **Scar burden by myocardial perfusion imaging predicts echocardiographic response to cardiac resynchronization therapy in ischemic cardiomyopathy**. *Am Heart J* 2007, **153**(1):105-112.
22. White JA, Yee R, Yuan X, Krahn A, Skanes A, Parker M, Klein G, Drangova M: **Delayed enhancement magnetic resonance imaging predicts response to cardiac resynchronization therapy in patients with intraventricular dyssynchrony**. *J Am Coll Cardiol* 2006, **48**(10):1953-1960.
23. Lipinski MJ, McVey CM, Berger JS, Kramer CM, Salerno M: **Prognostic value of stress cardiac magnetic resonance imaging in patients with known or suspected coronary artery disease: a systematic review and meta-analysis**. *J Am Coll Cardiol* 2013, **62**(9):826-838.
24. Greenwood JP, Maredia N, Younger JF, Brown JM, Nixon J, Everett CC, Bijsterveld P, Ridgway JP, Radjenovic A, Dickinson CJ *et al*: **Cardiovascular magnetic resonance and single-photon emission computed tomography for diagnosis of coronary heart disease (CE-MARC): a prospective trial**. *Lancet* 2012, **379**(9814):453-460.
25. Schwitter J, Wacker CM, Wilke N, Al-Saadi N, Sauer E, Huettle K, Schonberg SO, Debl K, Strohm O, Ahlstrom H *et al*: **Superior diagnostic performance of perfusion-cardiovascular magnetic resonance versus SPECT to detect coronary artery disease: The secondary endpoints of the multicenter multivendor MR-IMPACT II (Magnetic Resonance Imaging for Myocardial Perfusion Assessment in Coronary Artery Disease Trial)**. *Journal of cardiovascular magnetic resonance : official journal of the Society for Cardiovascular Magnetic Resonance* 2012, **14**:61.
26. Nagel E, Lehmkuhl HB, Bocksch W, Klein C, Vogel U, Frantz E, Ellmer A, Dreysse S, Fleck E: **Noninvasive diagnosis of ischemia-induced wall motion abnormalities with the use of high-dose dobutamine stress MRI: comparison with dobutamine stress echocardiography**. *Circulation* 1999, **99**(6):763-770.
27. Delewi R, Zijlstra F, Piek JJ: **Left ventricular thrombus formation after acute myocardial infarction**. *Heart* 2012, **98**(23):1743-1749.
28. Weinsaft JW, Kim RJ, Ross M, Krauser D, Manoushagian S, LaBounty TM, Cham MD, Min JK, Healy K, Wang Y *et al*: **Contrast-enhanced anatomic imaging as compared to contrast-**

For peer review only - http://bmjopen.bmj.com/site/about/guidelines.xhtml

enhanced tissue characterization for detection of left ventricular thrombus. *JACC Cardiovascular imaging* 2009, **2**(8):969-979.
29. Srichai MB, Junor C, Rodriguez LL, Stillman AE, Grimm RA, Lieber ML, Weaver JA, Smedira NG, White RD: **Clinical, imaging, and pathological characteristics of left ventricular thrombus: a comparison of contrast-enhanced magnetic resonance imaging, transthoracic echocardiography, and transesophageal echocardiography with surgical or pathological validation**. *Am Heart J* 2006, **152**(1):75-84.
30. Wong DT, Leung MC, Das R, Liew GY, Williams K, Dundon BK, Molaee P, Teo KS, Meredith IT, Worthley MI *et al*: **Diagnostic accuracy of adenosine stress cardiovascular magnetic resonance following acute ST-segment elevation myocardial infarction post primary angioplasty**. *Journal of Cardiovascular Magnetic Resonance* 2011, **13**:62.
31. Doesch C, Seeger A, Doering J, Herdeg C, Burgstahler C, Claussen CD, Gawaz M, Miller S, May AE: **Risk stratification by adenosine stress cardiac magnetic resonance in patients with coronary artery stenoses of intermediate angiographic severity**. *Jacc: Cardiovascular Imaging* 2009, **2**(4):424-433.
32. American College of Cardiology Foundation Task Force on Expert Consensus D, Hundley WG, Bluemke DA, Finn JP, Flamm SD, Fogel MA, Friedrich MG, Ho VB, Jerosch-Herold M, Kramer CM *et al*: **ACCF/ACR/AHA/NASCI/SCMR 2010 expert consensus document on cardiovascular magnetic resonance: a report of the American College of Cardiology Foundation Task Force on Expert Consensus Documents**. *Circulation* 2010, **121**(22):2462-2508.
33. Hoffmann U, Globits S, Schima W, Loewe C, Puig S, Oberhuber G, Frank H: **Usefulness of magnetic resonance imaging of cardiac and paracardiac masses**. *The American journal of cardiology* 2003, **92**(7):890-895.
34. Chan PG, Smith MP, Hauser TH, Yeon SB, Appelbaum E, Rofsky NM, Manning WJ: **Noncardiac pathology on clinical cardiac magnetic resonance imaging**. *JACC Cardiovascular imaging* 2009, **2**(8):980-986.
35. McKenna DA, Laxpati M, Colletti PM: **The prevalence of incidental findings at cardiac MRI**. *The open cardiovascular medicine journal* 2008, **2**:20-25.
36. Wyttenbach R, Medioni N, Santini P, Vock P, Szucs-Farkas Z: **Extracardiac findings detected by cardiac magnetic resonance imaging**. *Eur Radiol* 2012, **22**(6):1295-1302.

For peer review only

For peer review only - http://bmjopen.bmj.com/site/about/guidelines.xhtml

Appendix 3

Modified survey (statements, supporting paragraphs and references)

Accurate assessment of infarct characteristics is important for risk stratification after PPCI. CMR can quantify in a single scan all cardiac markers relevant to PPCI outcomes, with high reproducibility and accuracy. CMR has high spatial and temporal resolution and is superior to echocardiography for measuring left ventricular volumes and ejection fraction. **[1]** Additionally, CMR markers that cannot be measured by echocardiography, such as infarct size, microvascular obstruction and myocardial salvage have been shown to have long term prognostic value. **[2-4]** Late gadolinium enhancement CMR has added prognostic value over echocardiography. **[5]**

1. The following statements relate to the ability of CMR to identify patients who have a **poor** prognosis after PPCI.

   a. CMR markers (e.g. impaired left ventricular function, large infarct size, microvascular obstruction) better identify patients with a poor prognosis after PPCI than markers based on echocardiography.

Strongly disagree		Moderately disagree		Neither agree nor disagree		Moderately agree		Strongly agree
●	●	●	●	●	●	●	●	●

   b. Better identification of patients with a poor prognosis after PPCI allows these patients to be followed up more appropriately and treated more aggressively

Strongly disagree		Moderately disagree		Neither agree nor disagree		Moderately agree		Strongly agree
●	●	●	●	●	●	●	●	●

   c. More appropriate follow up and more aggressive treatment in these patients is expected to lead to a reduced risk of MACE in the long term.

Strongly disagree		Moderately disagree		Neither agree nor disagree		Moderately agree		Strongly agree
●	●	●	●	●	●	●	●	●

   d. More appropriate follow up and more aggressive treatment in these patients is expected to lead to less NHS resource use in the long term.

Strongly disagree		Moderately disagree		Neither agree nor disagree		Moderately agree		Strongly agree
●	●	●	●	●	●	●	●	●

   e. The anticipated benefit (reduced risk of MACE and/or less NHS resource use) is likely to be sufficiently large to make CMR cost effective among the patients in whom it would be indicated.

Strongly disagree		Moderately disagree		Neither agree nor disagree		Moderately agree		Strongly agree
●	●	●	●	●	●	●	●	●

For peer review only - http://bmjopen.bmj.com/site/about/guidelines.xhtml

2. The following statements relate to the ability of CMR to identify patients who have a **good** prognosis after PPCI.

   a. CMR markers (e.g. normal left ventricular function, high myocardial salvage, no microvascular obstruction, no residual ischemia) better identify patients with a good prognosis after PPCI than markers based on echocardiography.

Strongly disagree	Moderately disagree	Neither agree nor disagree	Moderately agree	Strongly agree

   b. Better identification of patients with a good prognosis after PPCI allows these patients to be followed up less frequently.

Strongly disagree	Moderately disagree	Neither agree nor disagree	Moderately agree	Strongly agree

   c. Less frequent follow up in these patients is expected to lead to less NHS resource use in the long term.

Strongly disagree	Moderately disagree	Neither agree nor disagree	Moderately agree	Strongly agree

   d. The anticipated benefit (less NHS resource use) is likely to be sufficiently large to make CMR cost effective among the patients in whom it would be indicated.

Strongly disagree	Moderately disagree	Neither agree nor disagree	Moderately agree	Strongly agree

For peer review only

Out-of-hospital cardiac arrest (OHCA) affects about 60,000 people in the UK each year. Currently, hospital survival among patients alive on arrival is about 32%. Early identification of the cause of OHCA is essential to improve survival. Causes of OHCA include myocardial infarction (40%-90%) and inherited cardiomyopathies such as arrhythmogenic right ventricular cardiomyopathy (ARVC) and hypertrophic cardiomyopathy (HCM). Unlike echocardiography, CMR allows *in vivo* tissue characterisation, which differentiates scarring due to myocardial infarction from other causes of focal fibrosis (e.g. observed in non-ischaemic cardiomyopathies such as ARVC and HCM).**[6]** Late gadolinium enhancement on CMR can predict serious cardiac complications in patients with HCM (all-cause death, cardiac death and death from heart failure),**[7]** and identifies patients that may need more aggressive medical and device therapy (e.g. renin-aldosterone system inhibition for prevention of heart failure or implantable cardioverter defibrillator placement for primary prevention of sudden cardiac death.

3. <u>The following statements relate to the ability of CMR to identify the causes of OHCA in patients who undergo an emergency angiogram.</u>

   a. CMR better identifies the cause of OHCA (e.g. large myocardial infarction, ARVC, aberrant coronary arteries, HCM) than echocardiography.

Strongly disagree	Moderately disagree	Neither agree nor disagree	Moderately agree	Strongly agree

   b. Better identification of the cause of out-of-hospital cardiac arrest allows treatment to be optimised for these patients (e.g. defibrillator for primary arrhythmia or percutaneous coronary intervention) or their family members (e.g. genetic screening and counselling, primary prevention).

Strongly disagree	Moderately disagree	Neither agree nor disagree	Moderately agree	Strongly agree

   c. The ability to optimise treatment for these patients or family members is expected to lead to a reduced risk of MACE in the long term.

Strongly disagree	Moderately disagree	Neither agree nor disagree	Moderately agree	Strongly agree

   d. The ability to optimise treatment for these patients or family members is expected to lead to less NHS resource use in the long term.

Strongly disagree	Moderately disagree	Neither agree nor disagree	Moderately agree	Strongly agree

   e. The anticipated benefit (reduced risk of MACE and/or less NHS resource use) is likely to be sufficiently large to make CMR cost effective among the patients in whom it would be indicated.

Strongly disagree	Moderately disagree	Neither agree nor disagree	Moderately agree	Strongly agree

For peer review only - http://bmjopen.bmj.com/site/about/guidelines.xhtml

Ventricular septal defect (VSD) and left ventricular free wall rupture are rare complications of myocardial infarction, occurring in less than 1% of PPCI patients, usually within one week of the infarct. Mortality ranges between 50% and 87%. Diagnosis usually requires multi-modality imaging (echocardiography, ventriculography, computed tomography, CMR) before surgical repair. Because of its high spatial resolution, CMR can be used to clarify the detailed structure of these lesions. CMR accurately identifies the location, size and tissue margins of VSD and is useful for detecting apical defects, which are not easily identified by echocardiography. CMR measurements may also be used to determine the size of ventricular patch required to close the VSD, which avoids the inflation of a sizing balloon in friable infarcted tissue. **[8]** These features, together with other CMR markers of damage (e.g. infarct size, microvascular obstruction, left ventricular dysfunction) are useful for guiding optimal management of patients with post-infarct VSD.

4. <u>The following statements relate to the ability of CMR to identify patients with VSD after myocardial infarction.</u>

    a. CMR identifies the location and characteristics of post-infarct VSD better than echocardiography.

Strongly disagree	Moderately disagree	Neither agree nor disagree	Moderately agree	Strongly agree

    b. Better identification of the location and characteristics of post-infarct VSD guides the optimal management of these patients.

Strongly disagree	Moderately disagree	Neither agree nor disagree	Moderately agree	Strongly agree

    c. Optimal management of patients with post-infarct VSD is expected to lead to a reduced risk of MACE in the long term.

Strongly disagree	Moderately disagree	Neither agree nor disagree	Moderately agree	Strongly agree

    d. Optimal management of patients with post-infarct VSD is expected to lead to less NHS resource use in the long term.

Strongly disagree	Moderately disagree	Neither agree nor disagree	Moderately agree	Strongly agree

    e. The anticipated benefit (reduced risk of MACE and/or less NHS resource use) is likely to be sufficiently large to make CMR cost effective among the patients in whom it would be indicated.

Strongly disagree	Moderately disagree	Neither agree nor disagree	Moderately agree	Strongly agree

For peer review only - http://bmjopen.bmj.com/site/about/guidelines.xhtml

Between 5% and 12% of patients who have an emergency angiogram are found to have unobstructed coronary arteries. In these patients, the lack of an accurate diagnosis may result in inappropriate or unnecessary treatment or follow-up and poorer prognosis. **[9]** CMR facilitates differential diagnosis in patients with unobstructed coronary arteries on angiography, providing a definitive diagnosis (e.g. myocardial infarction, myocarditis, Takotsubo cardiomyopathy) in 65-90% of these patients. **[10, 11]** In patients with myocarditis, late gadolinium enhancement (LGE) on CMR may predict long term adverse outcomes. **[12]** In the context of myocardial infarction without an angiographic lesion, CMR can locate the culprit infarct-related artery in patients with spontaneous reperfusion or with distal embolization.

5. <u>The following statements relate to the ability of CMR to differentiate myocaradial infarction from other diagnoses in patients found to have unobstructed coronary arteries on emergency angiography.</u>

   a. Unlike echocardiography, CMR can provide a definitive ischaemic diagnosis (e.g. myocardial infarction with spontaneous reperfusion or distal embolization) or a non-ischaemic diagnosis (e.g. myocarditis, Takotsubo cardiomyopathy, aortic dissection) in patients with unobstructed coronary arteries on angiography.

Strongly disagree	Moderately disagree	Neither agree nor disagree	Moderately agree	Strongly agree

   b. A definitive diagnosis results in a patient treatment plan appropriate for that diagnosis.

Strongly disagree	Moderately disagree	Neither agree nor disagree	Moderately agree	Strongly agree

   c. A treatment plan appropriate for the diagnosis is expected to lead to a reduced risk of MACE in the long term.

Strongly disagree	Moderately disagree	Neither agree nor disagree	Moderately agree	Strongly agree

   d. A treatment plan appropriate for the definitive diagnosis is expected to lead to less NHS resource use in the long term.

Strongly disagree	Moderately disagree	Neither agree nor disagree	Moderately agree	Strongly agree

   e. The anticipated benefit (reduced risk of MACE and/or less NHS resource use) is likely to be sufficiently large to make CMR cost effective among the patients in whom it would be indicated.

Strongly disagree	Moderately disagree	Neither agree nor disagree	Moderately agree	Strongly agree

For peer review only - http://bmjopen.bmj.com/site/about/guidelines.xhtml

About 6% of heart attack patients subsequently die suddenly from a presumed cardiac cause. Current guidelines recommend the use of an implantable cardioverter defibrillator (ICD) to prevent sudden cardiac death in patients who have a low left ventricular ejection fraction (EF) after a heart attack. EF is used in clinical practice to make decisions about ICD implantation, but it has a low predictive value and many patients with an ICD will never benefit from it. **[13]** EF is most commonly measured by echocardiography, but CMR is now considered the gold standard for EF measurement because it is more reproducible than echocardiography. **[1]** CMR has been shown to be better than echocardiography for selecting patients for ICD implantation when strict EF thresholds are used to guide implantation. **[14]** The extent of myocardial scar characterised by late gadolinium enhancement (LGE) CMR may also be used to predict whether ICD implantation is appropriate in this patient group. **[15]** Furthermore, LGE CMR can guide placement of the left ventricular lead away from scarred myocardium, which results in a better clinical outcome after cardiac resynchronisation therapy (CRT). **[16]**

6. The following statements relate to the ability of CMR to identify patients at high risk of sudden cardiac death after PPCI who would benefit most from an implantable cardiac device.

   a. CMR identifies PPCI patients who are at high risk of sudden cardiac death better than echocardiography.

Strongly disagree	Moderately disagree	Neither agree nor disagree	Moderately agree	Strongly agree

   b. Better identification of PPCI patients at high risk of sudden cardiac death allows optimal patient selection for an implantable cardiac device (ICD or CRT).

Strongly disagree	Moderately disagree	Neither agree nor disagree	Moderately agree	Strongly agree

   c. Optimal patient selection for an implantable cardiac device is expected to lead to a reduced risk of MACE in these patients in the long term.

Strongly disagree	Moderately disagree	Neither agree nor disagree	Moderately agree	Strongly agree

   d. Optimal patient selection for an implantable cardiac device is expected to lead to less NHS resource use in the long term.

Strongly disagree	Moderately disagree	Neither agree nor disagree	Moderately agree	Strongly agree

   e. The anticipated benefit (reduced risk of MACE and/or less NHS resource use) is likely to be sufficiently large to make CMR cost effective among the patients in whom it would be indicated.

Strongly disagree	Moderately disagree	Neither agree nor disagree	Moderately agree	Strongly agree

For peer review only - http://bmjopen.bmj.com/site/about/guidelines.xhtml

Cardiac resynchronisation therapy (CRT), or biventricular pacing, uses a specialised pacemaker to improve the contraction of the left ventricle and the overall efficiency of the heart. It is used in patients with systolic ventricular dysfunction and heart failure. However, about 30% of patients who meet the inclusion criteria for CRT do not respond to it. **[17]** CMR assessment of mechanical dyssynchrony and myocardial scar provides additional value over echocardiography for identifying non responsive patients. **[18, 19]** Myocardial scar is an important feature of non-response to CRT **[20, 21]** and late gadolinium enhancement (LGE) CMR accurately differentiates between transmural, mid-myocardial, epicardial, and subendocardial scar.

7. The following statements relate to the ability of CMR to identify patients who **would not** benefit from CRT after PPCI.

   a. CMR identifies patients who would not benefit from CRT better than echocardiography.

Strongly disagree	Moderately disagree	Neither agree nor disagree	Moderately agree	Strongly agree

   b. The ability to identify patients who would not benefit from CRT would reduce CRT use in patients who do not need it.

Strongly disagree	Moderately disagree	Neither agree nor disagree	Moderately agree	Strongly agree

   c. Reducing CRT use in patients who do not need it is expected to lead to reduced risk of MACE in these patients in the long term.

Strongly disagree	Moderately disagree	Neither agree nor disagree	Moderately agree	Strongly agree

   d. Reducing CRT use in patients who do not need it is expected to lead to less NHS resource use in the long term.

Strongly disagree	Moderately disagree	Neither agree nor disagree	Moderately agree	Strongly agree

   e. The anticipated benefit (reduced risk of MACE and/or less NHS resource use) is likely to be sufficiently large to make CMR cost effective among the patients in whom it would be indicated.

Strongly disagree	Moderately disagree	Neither agree nor disagree	Moderately agree	Strongly agree

For peer review only - http://bmjopen.bmj.com/site/about/guidelines.xhtml

Between 40% and 65% of the patients who activate the PPCI pathway have multi-vessel disease. Adequate assessment of residual ischemia in non-culprit arteries post PPCI is important for effective management because this patient group has an adverse prognosis. Various techniques are currently used to assess the need for additional revascularisation, including stress echocardiography, single photon emission computed tomography (SPECT) and stress-perfusion CMR. Stress CMR has excellent prognostic value **[22]** and better diagnostic accuracy than SPECT **[23, 24]** or stress echocardiography **[25]** for detecting coronary artery disease causing significant regional myocardial ischaemia.

8. The following statements relate to the ability of CMR to assess ischemia and viability in patients with multi-vessel disease.

   a. CMR assesses ischemia and viability of the myocardium better than echocardiography.

Strongly disagree	Moderately disagree	Neither agree nor disagree	Moderately agree	Strongly agree

   b. Better assessment of ischemia and viability of the myocardium optimises the revascularisation strategy for patients with multi-vessel disease and avoids additional diagnostic tests.

Strongly disagree	Moderately disagree	Neither agree nor disagree	Moderately agree	Strongly agree

   c. The ability to optimise the revascularisation strategy for patients with multi-vessel disease is expected to lead to a reduced risk of MACE in the long term.

Strongly disagree	Moderately disagree	Neither agree nor disagree	Moderately agree	Strongly agree

   f. The ability to optimise the revascularisation strategy and avoid additional diagnostic tests in patients with multi-vessel disease is expected to lead to less NHS resource use in the long term.

Strongly disagree	Moderately disagree	Neither agree nor disagree	Moderately agree	Strongly agree

   d. The anticipated benefit (reduced risk of MACE and/or less NHS resource use) is likely to be sufficiently large to make CMR cost effective among the patients in whom it would be indicated.

Strongly disagree	Moderately disagree	Neither agree nor disagree	Moderately agree	Strongly agree

For peer review only

For peer review only - http://bmjopen.bmj.com/site/about/guidelines.xhtml

Left ventricular (LV) thrombus is a serious complication of acute myocardial infarction. It increases risk of thromboembolic events, particularly stroke. LV thrombus develops in up to 10% of patients with anterior wall infarction after PPCI. Although echocardiography is most commonly used to detect LV thrombus and to assess its shape and size, between 10-46% of echocardiograms are inconclusive. **[26]** Late gadolinium enhancement (LGE) CMR is considered the gold standard for detecting LV thrombus, because it detects thrombus based on tissue characteristics rather than anatomic appearance. Fewer thrombi are detected by contrast-echocardiography than by LGE CMR. **[27]** CMR has a higher sensitivity (88%) than contrast (61%) and noncontrast (<33%) echocardiography. **[27, 28]**

9. <u>The following statements relate to the ability of CMR to identify patients with post-infarct LV thrombus.</u>

   a. CMR identifies post-infarct LV thrombus better than echocardiography.

Strongly disagree		Moderately disagree		Neither agree nor disagree		Moderately agree		Strongly agree
○	○	○	○	○	○	○	○	○

   b. Better detection of post-infarct LV thrombus in PPCI patients allows more affected patients to be treated with anticoagulation therapy.

Strongly disagree		Moderately disagree		Neither agree nor disagree		Moderately agree		Strongly agree
○	○	○	○	○	○	○	○	○

   c. Treatment with anticoagulation therapy in patients with post-infarct LV thrombus is expected to lead to a reduced risk of MACE in the long term.

Strongly disagree		Moderately disagree		Neither agree nor disagree		Moderately agree		Strongly agree
○	○	○	○	○	○	○	○	○

   d. Treatment with anticoagulation therapy in patients with post-infarct LV thrombus is expected to lead to less NHS resource use in the long term.

Strongly disagree		Moderately disagree		Neither agree nor disagree		Moderately agree		Strongly agree
○	○	○	○	○	○	○	○	○

   e. The anticipated benefit (reduced risk of MACE and/or less NHS resource use) is likely to be sufficiently large to make CMR cost effective among the patients in whom it would be indicated.

Strongly disagree		Moderately disagree		Neither agree nor disagree		Moderately agree		Strongly agree
○	○	○	○	○	○	○	○	○

For peer review only - http://bmjopen.bmj.com/site/about/guidelines.xhtml

Compared with echocardiography, CMR provides versatile imaging planes, superior tissue contrast and advanced tissue characterisation, allowing a comprehensive assessment of cardiac anatomy, function and flow, and imaging of the great vessels (including venous return), the pericardium and suspected cardiac tumours. **[29]** As such, CMR can identify congenital coronary anomalies, cardiac masses, coronary artery aneurysms, valvular heart disease, thoracic aortic disease, etc. CMR can adequately differentiate benign from malignant tumours in the heart. **[30]** Unlike echocardiography, which provides limited imaging of mediastinal and extra-cardiac structures, CMR also images a substantial part of the thorax and abdomen in the field of view, which may potentially contain non-cardiac abnormalities. The prevalence of non-cardiac findings on CMR is up to 80% (with up to 30% of these representing potentially significant findings), depending on the characteristics of the population examined. **[31-33]**

10. The following statements relate to the ability of CMR to detect incidental cardiac and non-cardiac findings if offered routinely to patients who undergo an emergency angiogram.

a. CMR identifies more incidental cardiac / non-cardiac findings than echocardiography.

Strongly disagree	Moderately disagree	Neither agree nor disagree	Moderately agree	Strongly agree

b. Improved detection of potentially significant incidental findings allows affected patients to be investigated further and / or treated.

Strongly disagree	Moderately disagree	Neither agree nor disagree	Moderately agree	Strongly agree

c. Further investigation and treatment is expected to reduce the risk of MACE / increase overall survival in affected patients in the long term.

Strongly disagree	Moderately disagree	Neither agree nor disagree	Moderately agree	Strongly agree

d. Further investigation and treatment is expected to lead to less NHS resource use in the long term.

Strongly disagree	Moderately disagree	Neither agree nor disagree	Moderately agree	Strongly agree

e. The anticipated benefit (reduced risk of MACE and/or less NHS resource use) is likely to be sufficiently large to make CMR cost effective among the patients who undergo an emergency angiogram.

Strongly disagree	Moderately disagree	Neither agree nor disagree	Moderately agree	Strongly agree

For peer review only - http://bmjopen.bmj.com/site/about/guidelines.xhtml

References

1. Dorosz JL, Lezotte DC, Weitzenkamp DA, Allen LA, Salcedo EE: **Performance of 3-dimensional echocardiography in measuring left ventricular volumes and ejection fraction: a systematic review and meta-analysis**. *J Am Coll Cardiol* 2012, **59**(20):1799-1808.
2. Eitel I, Desch S, de WS, Fuernau G, Gutberlet M, Schuler G, Thiele H: **Long-term prognostic value of myocardial salvage assessed by cardiovascular magnetic resonance in acute reperfused myocardial infarction**. *Heart* 2011, **97**(24):2038-2045.
3. Eitel I, Desch S, Fuernau G, Hildebrand L, Gutberlet M, Schuler G, Thiele H: **Prognostic significance and determinants of myocardial salvage assessed by cardiovascular magnetic resonance in acute reperfused myocardial infarction**. *J Am Coll Cardiol* 2010, **55**(22):2470-2479.
4. Klug G, Mayr A, Schenk S, Esterhammer R, Schocke M, Nocker M, Jaschke W, Pachinger O, Metzler B: **Prognostic value at 5 years of microvascular obstruction after acute myocardial infarction assessed by cardiovascular magnetic resonance**. *Journal of Cardiovascular Magnetic Resonance* 2012, **14**:46.
5. Wong TC, Piehler K, Puntil KS, Moguillansky D, Meier CG, Lacomis JM, Kellman P, Cook SC, Schwartzman DS, Simon MA *et al*: **Effectiveness of late gadolinium enhancement to improve outcomes prediction in patients referred for cardiovascular magnetic resonance after echocardiography**. *Journal of cardiovascular magnetic resonance : official journal of the Society for Cardiovascular Magnetic Resonance* 2013, **15**:6.
6. Parsai C, O'Hanlon R, Prasad SK, Mohiaddin RH: **Diagnostic and prognostic value of cardiovascular magnetic resonance in non-ischaemic cardiomyopathies**. *Journal of cardiovascular magnetic resonance : official journal of the Society for Cardiovascular Magnetic Resonance* 2012, **14**:54.
7. Green JJ, Berger JS, Kramer CM, Salerno M: **Prognostic value of late gadolinium enhancement in clinical outcomes for hypertrophic cardiomyopathy**. *JACC Cardiovascular imaging* 2012, **5**(4):370-377.
8. Artis NJ, Thomson J, Plein S, Greenwood JP: **Percutaneous closure of postinfarction ventricular septal defect: cardiac magnetic resonance-guided case selection and postprocedure evaluation**. *The Canadian journal of cardiology* 2011, **27**(6):869 e863-865.
9. Dokainish H, Pillai M, Murphy SA, DiBattiste PM, Schweiger MJ, Lotfi A, Morrow DA, Cannon CP, Braunwald E, Lakkis N: **Prognostic implications of elevated troponin in patients with suspected acute coronary syndrome but no critical epicardial coronary disease: a TACTICS-TIMI-18 substudy**. *J Am Coll Cardiol* 2005, **45**(1):19-24.
10. Assomull RG, Lyne JC, Keenan N, Gulati A, Bunce NH, Davies SW, Pennell DJ, Prasad SK: **The role of cardiovascular magnetic resonance in patients presenting with chest pain, raised troponin, and unobstructed coronary arteries**. *Eur Heart J* 2007, **28**(10):1242-1249.
11. Leurent G, Langella B, Fougerou C, Lentz PA, Larralde A, Bedossa M, Boulmier D, Le Breton H: **Diagnostic contributions of cardiac magnetic resonance imaging in patients presenting with elevated troponin, acute chest pain syndrome and unobstructed coronary arteries**. *Arch Cardiovasc Dis* 2011, **104**(3):161-170.
12. Grun S, Schumm J, Greulich S, Wagner A, Schneider S, Bruder O, Kispert EM, Hill S, Ong P, Klingel K *et al*: **Long-term follow-up of biopsy-proven viral myocarditis: predictors of mortality and incomplete recovery**. *J Am Coll Cardiol* 2012, **59**(18):1604-1615.
13. Moss AJ, Greenberg H, Case RB, Zareba W, Hall WJ, Brown MW, Daubert JP, McNitt S, Andrews ML, Elkin AD *et al*: **Long-term clinical course of patients after termination of ventricular tachyarrhythmia by an implanted defibrillator**. *Circulation* 2004, **110**(25):3760-3765.
14. Joshi SB, Connelly KA, Jimenez-Juan L, Hansen M, Kirpalani A, Dorian P, Mangat I, Al-Hesayen A, Crean AM, Wright GA *et al*: **Potential clinical impact of cardiovascular magnetic**

For peer review only - http://bmjopen.bmj.com/site/about/guidelines.xhtml

resonance assessment of ejection fraction on eligibility for cardioverter defibrillator implantation. *Journal of cardiovascular magnetic resonance : official journal of the Society for Cardiovascular Magnetic Resonance* 2012, **14**:69.

15. Alexandre J, Saloux E, Dugue AE, Lebon A, Lemaitre A, Roule V, Labombarda F, Provost N, Gomes S, Scanu P *et al*: **Scar extent evaluated by late gadolinium enhancement CMR: a powerful predictor of long term appropriate ICD therapy in patients with coronary artery disease**. *Journal of cardiovascular magnetic resonance : official journal of the Society for Cardiovascular Magnetic Resonance* 2013, **15**:12.
16. Leyva F, Foley PW, Chalil S, Ratib K, Smith RE, Prinzen F, Auricchio A: **Cardiac resynchronization therapy guided by late gadolinium-enhancement cardiovascular magnetic resonance**. *Journal of cardiovascular magnetic resonance : official journal of the Society for Cardiovascular Magnetic Resonance* 2011, **13**:29.
17. Leclercq C, Kass DA: **Retiming the failing heart: principles and current clinical status of cardiac resynchronization**. *J Am Coll Cardiol* 2002, **39**(2):194-201.
18. Bilchick KC, Dimaano V, Wu KC, Helm RH, Weiss RG, Lima JA, Berger RD, Tomaselli GF, Bluemke DA, Halperin HR *et al*: **Cardiac magnetic resonance assessment of dyssynchrony and myocardial scar predicts function class improvement following cardiac resynchronization therapy**. *JACC Cardiovascular imaging* 2008, **1**(5):561-568.
19. Ellims AH, Pfluger H, Elsik M, Butler MJ, Hare JL, Taylor AJ: **Utility of cardiac magnetic resonance imaging, echocardiography and electrocardiography for the prediction of clinical response and long-term survival following cardiac resynchronisation therapy**. *Int J Cardiovasc Imaging* 2013, **29**(6):1303-1311.
20. Adelstein EC, Saba S: **Scar burden by myocardial perfusion imaging predicts echocardiographic response to cardiac resynchronization therapy in ischemic cardiomyopathy**. *Am Heart J* 2007, **153**(1):105-112.
21. White JA, Yee R, Yuan X, Krahn A, Skanes A, Parker M, Klein G, Drangova M: **Delayed enhancement magnetic resonance imaging predicts response to cardiac resynchronization therapy in patients with intraventricular dyssynchrony**. *J Am Coll Cardiol* 2006, **48**(10):1953-1960.
22. Lipinski MJ, McVey CM, Berger JS, Kramer CM, Salerno M: **Prognostic value of stress cardiac magnetic resonance imaging in patients with known or suspected coronary artery disease: a systematic review and meta-analysis**. *J Am Coll Cardiol* 2013, **62**(9):826-838.
23. Greenwood JP, Maredia N, Younger JF, Brown JM, Nixon J, Everett CC, Bijsterveld P, Ridgway JP, Radjenovic A, Dickinson CJ *et al*: **Cardiovascular magnetic resonance and single-photon emission computed tomography for diagnosis of coronary heart disease (CE-MARC): a prospective trial**. *Lancet* 2012, **379**(9814):453-460.
24. Schwitter J, Wacker CM, Wilke N, Al-Saadi N, Sauer E, Huettle K, Schonberg SO, Debl K, Strohm O, Ahlstrom H *et al*: **Superior diagnostic performance of perfusion-cardiovascular magnetic resonance versus SPECT to detect coronary artery disease: The secondary endpoints of the multicenter multivendor MR-IMPACT II (Magnetic Resonance Imaging for Myocardial Perfusion Assessment in Coronary Artery Disease Trial)**. *Journal of cardiovascular magnetic resonance : official journal of the Society for Cardiovascular Magnetic Resonance* 2012, **14**:61.
25. Nagel E, Lehmkuhl HB, Bocksch W, Klein C, Vogel U, Frantz E, Ellmer A, Dreysse S, Fleck E: **Noninvasive diagnosis of ischemia-induced wall motion abnormalities with the use of high-dose dobutamine stress MRI: comparison with dobutamine stress echocardiography**. *Circulation* 1999, **99**(6):763-770.
26. Delewi R, Zijlstra F, Piek JJ: **Left ventricular thrombus formation after acute myocardial infarction**. *Heart* 2012, **98**(23):1743-1749.
27. Weinsaft JW, Kim RJ, Ross M, Krauser D, Manoushagian S, LaBounty TM, Cham MD, Min JK, Healy K, Wang Y *et al*: **Contrast-enhanced anatomic imaging as compared to contrast-**

For peer review only - http://bmjopen.bmj.com/site/about/guidelines.xhtml

enhanced tissue characterization for detection of left ventricular thrombus. *JACC Cardiovascular imaging* 2009, **2**(8):969-979.

28. Srichai MB, Junor C, Rodriguez LL, Stillman AE, Grimm RA, Lieber ML, Weaver JA, Smedira NG, White RD: **Clinical, imaging, and pathological characteristics of left ventricular thrombus: a comparison of contrast-enhanced magnetic resonance imaging, transthoracic echocardiography, and transesophageal echocardiography with surgical or pathological validation**. *Am Heart J* 2006, **152**(1):75-84.
29. American College of Cardiology Foundation Task Force on Expert Consensus D, Hundley WG, Bluemke DA, Finn JP, Flamm SD, Fogel MA, Friedrich MG, Ho VB, Jerosch-Herold M, Kramer CM *et al*: **ACCF/ACR/AHA/NASCI/SCMR 2010 expert consensus document on cardiovascular magnetic resonance: a report of the American College of Cardiology Foundation Task Force on Expert Consensus Documents**. *Circulation* 2010, **121**(22):2462-2508.
30. Hoffmann U, Globits S, Schima W, Loewe C, Puig S, Oberhuber G, Frank H: **Usefulness of magnetic resonance imaging of cardiac and paracardiac masses**. *The American journal of cardiology* 2003, **92**(7):890-895.
31. Chan PG, Smith MP, Hauser TH, Yeon SB, Appelbaum E, Rofsky NM, Manning WJ: **Noncardiac pathology on clinical cardiac magnetic resonance imaging**. *JACC Cardiovascular imaging* 2009, **2**(8):980-986.
32. McKenna DA, Laxpati M, Colletti PM: **The prevalence of incidental findings at cardiac MRI**. *The open cardiovascular medicine journal* 2008, **2**:20-25.
33. Wyttenbach R, Medioni N, Santini P, Vock P, Szucs-Farkas Z: **Extracardiac findings detected by cardiac magnetic resonance imaging**. *Eur Radiol* 2012, **22**(6):1295-1302.

For peer review only

For peer review only - http://bmjopen.bmj.com/site/about/guidelines.xhtml

Appendix 4

Median and interquartile (IQ) range for the 12 statements in the first survey (n=7). Boxes represent the median and IQ range and the whiskers represent the range. Dots represent extreme values. Statement 3, 5 and 9 were considered to be in consensus (median score of ≥7and IQ range of 6-9).

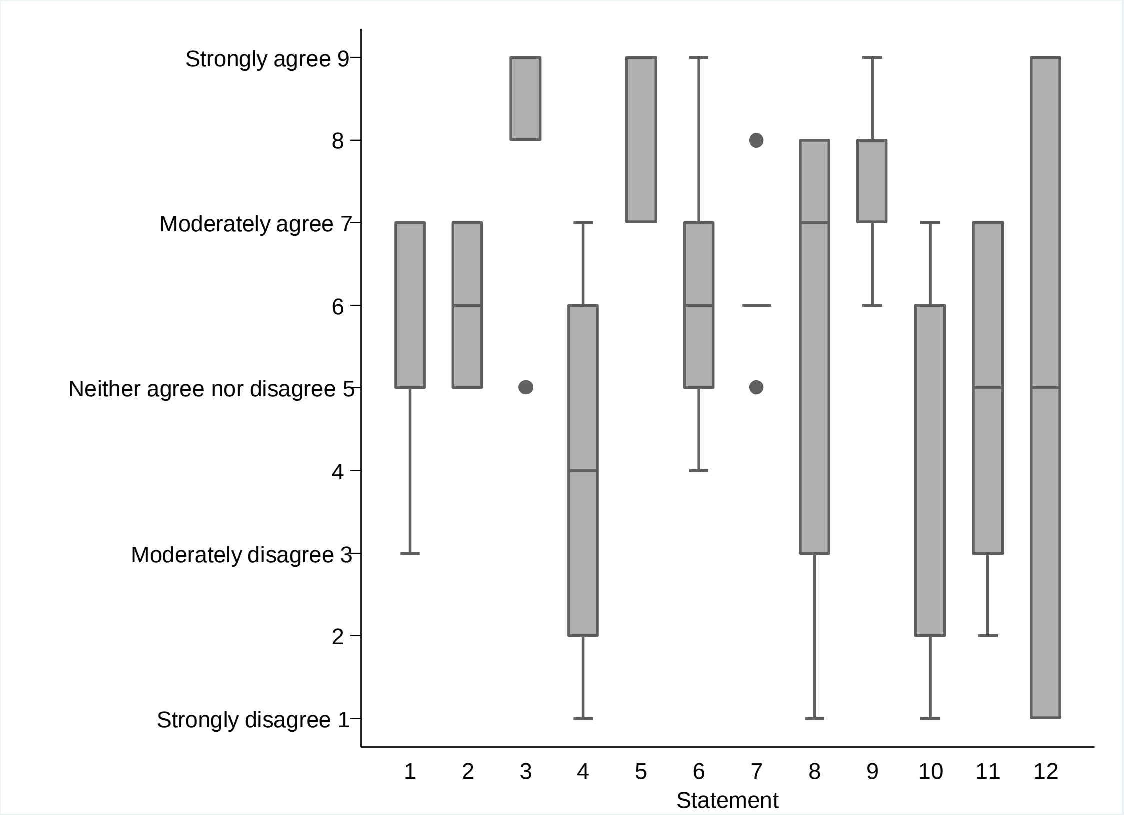

Supplement: Author's manuscript [file bmjopen-2016-014627.draft_revisions.pdf]
